# Identification of Low-Complexity Domains by Compositional Signatures Reveals Class-Specific Frequencies and Functions Across the Domains of Life

**DOI:** 10.1371/journal.pcbi.1011372

**Published:** 2024-05-15

**Authors:** Sean M. Cascarina, Eric D. Ross

**Affiliations:** Department of Biochemistry and Molecular Biology, Colorado State University, Fort Collins, Colorado, United States of America; CPERI, GREECE

## Abstract

Low-complexity domains (LCDs) in proteins are typically enriched in one or two predominant amino acids. As a result, LCDs often exhibit unusual structural/biophysical tendencies and can occupy functional niches. However, for each organism, protein sequences must be compatible with intracellular biomolecules and physicochemical environment, both of which vary from organism to organism. This raises the possibility that LCDs may occupy sequence spaces in select organisms that are otherwise prohibited in most organisms. Here, we report a comprehensive survey and functional analysis of LCDs in all known reference proteomes (>21k organisms), with added focus on rare and unusual types of LCDs. LCDs were classified according to both the primary amino acid and secondary amino acid in each LCD sequence, facilitating detailed comparisons of LCD class frequencies across organisms. Examination of LCD classes at different depths (i.e., domain of life, organism, protein, and per-residue levels) reveals unique facets of LCD frequencies and functions. To our surprise, all 400 LCD classes occur in nature, although some are exceptionally rare. A number of rare classes can be defined for each domain of life, with many LCD classes appearing to be eukaryote-specific. Certain LCD classes were consistently associated with identical functions across many organisms, particularly in eukaryotes. Our analysis methods enable simultaneous, direct comparison of all LCD classes between individual organisms, resulting in a proteome-scale view of differences in LCD frequencies and functions. Together, these results highlight the remarkable diversity and functional specificity of LCDs across all known life forms.

## Author summary

Many protein sequences contain “low-complexity domains” (LCDs), which are regions in the sequence mostly comprised of only one or two different types of amino acids. Since there are 20 main types of amino acids found in natural proteins, many “flavors” of LCDs are possible, with each flavor having unique structural and functional properties. However, the functions and prevalence of each type of LCD across organisms has not been explored extensively. In this study, we divided LCDs into 400 categories based on the one or two amino acids that were most common in each LCD sequence. Then, using a representative set of all known organisms, we examined how prevalent each type of LCD was and which functions were most often linked to each type of LCD. We uncovered LCD functions common to many organisms as well as LCD functions restricted to certain organisms or LCD types. Some organisms had unusually high levels of specific types of LCDs, suggesting that adaptations in these organisms or special conditions in their environment have aided in the tolerance–or even usage–of those LCD types. Our results give both broad and in-depth views of LCDs, their functions, and their frequencies in nature.

## Introduction

In known organisms, the majority of proteome space is devoted to “high-complexity” regions comprised of a diverse mixture of amino acids. Nearly all organisms also contain “low-complexity” domains (LCDs), which have skewed amino acid compositions often favoring one or two amino acids [1]. As such, LCDs are a heterogeneous collection of domains with highly divergent biochemical and biophysical properties that depend upon which specific amino acid(s) are enriched in each domain. For example, LCDs enriched in polar and charged amino acids are often intrinsically disordered [2–6], whereas LCDs enriched in other amino acids exhibit a greater tendency to adopt ordered conformations [7,8]. Classification of LCDs based on their primary and secondary amino acids (i.e., the most common and second-most common amino acids, respectively, within the LCD) results in a hierarchy of LCD categories that correlate with specific molecular functions [9,10]. Consequently, such classification schemes are vital for properly interpreting statistical associations between LCDs and molecular functions. Statistics performed on LCDs as a single category will be heavily skewed by the predominant LCD classes in a given dataset, so conflation of distinct LCD categories can lead to overgeneralized and imprecise conclusions.

LCDs from each class differ in their abundance within and across organisms [9,11,12]. One plausible explanation for these differences is that particular classes of LCDs are tolerated or beneficial to varying degrees in different organisms. A variety of LCDs from multiple LCD classes undergo liquid-liquid phase separation *in vitro* and enable recruitment to biomolecular condensates *in vivo* in response to changes in their surrounding environment (for review, see [13–18]). These changes include shifts in simple physicochemical properties such as temperature, pH, salt concentration, osmolarity, and pressure, as well as large-scale alterations in the abundance of free biomolecules such as RNA, ATP, polyphosphate, polyamines, and polyADP-ribose. In some cases, LCDs act as direct sensors of physicochemical environment in various contexts and in a manner specific to both the LCD type and the change in environment. For example, S/T-rich LCDs were recently linked to CO_2_ sensing in *Candida albicans* [19], M-rich LCDs have been associated with redox sensing in *Saccharomyces cerevisiae* [20,21], Q/H-rich LCDs have been associated with pH sensing in *Drosophila melanogaster* [22,23], and Q-rich LCDs have been linked to temperature sensing in *Arabidopsis thaliana* [24].

While some environmental conditions are buffered by non-equilibrium, physiological regulatory systems, the full complement of known species spans a broad range of ecological niches. Such a diverse collection of niches likely necessitates proteome-scale adaptations in the organisms that occupy them. Given the remarkable responsiveness and environmental sensitivity of LCDs, we reasoned that at least one of the proteome-scale adaptations could be the overall LCD content profile for each organism, including both the abundance of LCDs from each LCD class and the features of those LCDs. Certain LCDs that might be maladaptive to a particular organism could be benign or even functional to a different organism in a different context. Additionally, specific organisms may develop concomitant proteome adaptations that influence LCD tolerability. For instance, specific adaptations in the proteostasis machinery of the model slime mold, *Dictyostelium discoideum*, and of the malarial parasite, *Plasmodium falciparum*, suppress the *in vivo* aggregation of N-rich LCDs [25–28], which are notoriously aggregation-prone and are remarkably abundant in their proteomes. Similarly, K/Y-rich and Y/W/G-rich LCDs in secreted mussel adhesion proteins depend upon post-translational modification of Y residues for their adhesive functions [29,30]. This process is sensitive to disruption by oxidative damage, but this damage is buffered by co-secreted proteins [31]. Broadly, these examples highlight: 1) LCDs that would likely be deleterious in many organisms can have beneficial functions in specific organisms and contexts, and 2) co-adaptations in the proteomes of these organisms enable the functions of these LCDs and/or mitigate their potential negative consequences.

Many unresolved questions arise from this line of reasoning. Are all types of LCDs observed in nature? What are the limits of LCD content that are compatible with known life forms? Are rare LCDs endowed with specialized functions in particular sets of organisms? To begin to address these questions, we performed a comprehensive survey of LCDs in a complete set of reference proteomes. Our method of categorizing LCDs enables detailed quantification of LCD abundance for each LCD class and direct comparisons of whole-proteome LCD content across organisms for each LCD class. Deconvolution of LCD classes facilitates direct functional analysis of each LCD class within and across the domains of life, providing the first global view of LCD functions in a manner specific to each LCD class among known organisms. Finally, we explore rare and unusual LCDs at multiple levels (i.e., per-organism, per-protein, and per-residue levels) and discuss the unique insights offered by each layer, including functions associated with specific classes of LCDs.

## Results

### Measures of LCD Categorization and LCD Rarity

LCDs can be categorized based on the most-enriched amino acid in each LCD sequence, resulting in 20 “primary” LCD classes (one for each of the 20 canonical amino acids). Primary LCD classes differ in their frequencies and abundance across organisms [9], which is likely due to factors including (but not limited to) whole-proteome amino acid frequencies and/or specialized LCD functions. Additionally, primary LCD classes can be further decomposed into “secondary” LCD classes defined by a second amino acid that is co-enriched in the LCD sequence, resulting in an LCD classification hierarchy that correlates with functional specificity [9]. In order to identify instances of primary and secondary LCD classes, a set of UniProt reference proteomes was evaluated using the *LCD Compo*sition *S*cann*er* (LCD-Composer [9,32]; see Methods). Primary LCD classes are here defined as regions ≥20 amino acids in length with ≥40% of the composition corresponding to a single type of amino acid. LCDs can be further classified into “secondary” LCD classes, here defined as regions with ≥40% of the composition corresponding to a single “primary” amino acid and ≥20% of the composition corresponding to a “secondary” amino acid (e.g., S-rich LCDs with a secondary bias for R would be instances of the “SR” secondary LCD class). We have previously demonstrated that these window sizes and compositional thresholds are effective at classifying and functionally categorizing LCDs with high specificity [9]. Using this classification scheme, LCD “rarity” was then examined on a variety of related levels (discussed in separate sections below), each with unique implications and insights. Complete datasets of primary and secondary LCDs for all UniProt reference proteomes are available in a publicly accessible repository [33].

### Organism-level LCD Class Frequencies

**Fig 1:**
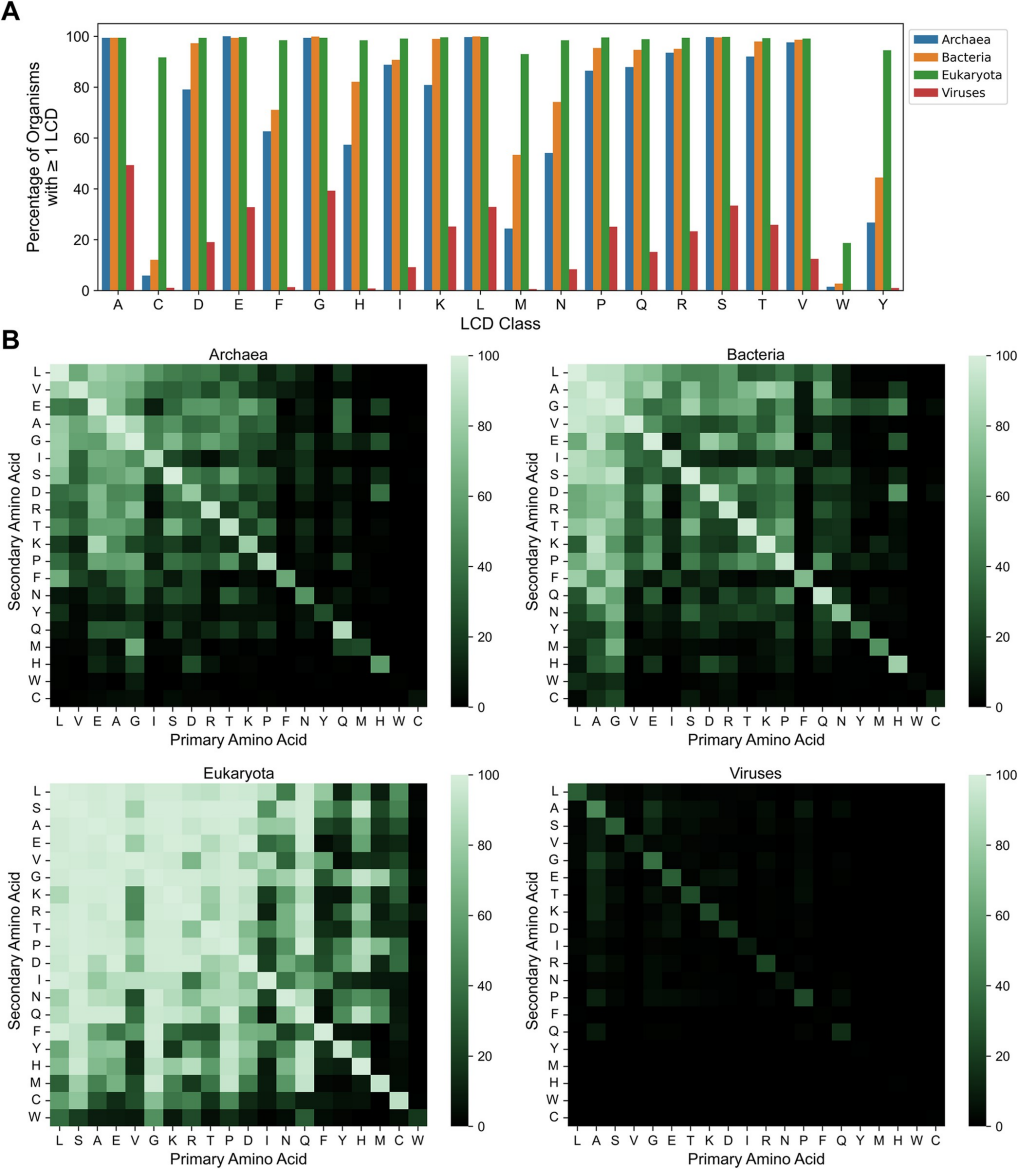
Organism-level LCD frequencies for primary and secondary LCD classes among each domain of life. (A) Percentage of organisms with ≥1 LCD instance for each of the 20 classes of primary LCDs across the four major domains of life. (B) Heatmaps indicating the percentage of organisms from each domain of life with ≥1 instance of each secondary LCD class. In each plot, the *x*-axis represents the primary amino acid enriched in the LCD (≥40% composition) and the *y*-axis represents the secondary amino acid enriched in the LCD (≥20% composition). Values for primary LCD classes are indicated in the diagonals to facilitate relative comparisons. Amino acids in panel B were sorted by average whole-proteome frequency rank.

We first examined what percentage of organisms contain at least one instance of each class of LCDs, which we term “organism-level LCD frequency”. For most primary LCD classes, a moderate to high percentage of organisms contain at least one protein with an LCD of that class (Figs 1 and S1, and S1–S5 Tables). Consistent with previous observations [9,12], organism-level LCD frequency tends to increase in the order of Viruses➔Archaea➔Bacteria➔Eukaryota, with many viruses often lacking an LCD, most likely due to small proteomes. Interestingly, ≥90% of all eukaryotes contain at least one instance for each primary LCD class except W-rich LCDs, which were rare across all 4 domains of life (Fig 1A). W is typically rare in proteomes, which likely contributes to the observed rarity. However, other LCD classes represented by rare amino acids (e.g. C, M, and H) are much more common among eukaryotes and occur in other domains of life, potentially suggesting that W-rich LCDs are uniquely limited by selection or lack beneficial activities. Remarkably, none of the 380 secondary LCD classes are completely absent in nature (Figs 1B and S1), though some are exceedingly rare (e.g., only 6 total WM LCDs were identified) and likely require experimental validation.

Each of the major domains of life exhibits similar trends with respect to the percentage of organisms containing at least one instance of each two-residue LCD class, a measure that we will refer to as LCD class representation (Fig 1B and S2–S5 Tables). Viruses exhibit universally low LCD class representation (Fig 1B, *lower right*). For the remaining domains of life, the most prevalent LCD classes tend to be composed of amino acids with high whole-proteome frequencies. However, there are noteworthy exceptions. First, some amino acids with moderate to high whole-proteome frequencies (e.g., V or I) exhibit relatively low LCD class representation (see Archaea, Bacteria, and Eukaryota in Fig 1B). Second, some amino acids that have low-whole-proteome frequencies (e.g., Q and H in eukaryotes) exhibit relatively high LCD class representation. Third, some rare primary LCD classes exhibit specifically higher representation among subclasses of secondary amino acids. For both Y-rich and M-rich primary LCD classes, G is the most common secondary residue in eukaryotes; these YG and MG classes are also detected to a lesser extent in archaea and bacteria (Fig 1B). It is worth noting that our method intentionally does not impose an upper-bound restriction on the degree of enrichment of the secondary amino acid, only minimum requirements for both the primary and secondary amino acids. This may result in some degree of overlap in reciprocal secondary LCD classes (e.g., GM and MG classes). Therefore, while these LCD classes may include LCDs with ≥40% Y or M and still higher G content, these rare amino acids are nevertheless enriched to an unusual degree, and this is very specific to certain subcategories of LCDs.

To explore whether the comparisons of LCD frequencies between domains of life depend on the composition thresholds and window size, we re-evaluated the proteomes of a randomly chosen subset of organisms from each domain (S6 Table) while varying these two parameters independently during the LCD searches. As expected, more stringent search parameters (higher percent composition or window size thresholds) resulted in fewer LCDs across all domains of life, but the same general trend of organism-level LCD frequency is apparent: the percentage of organisms containing at least one LCD increases in the order of Viruses➔Archaea➔Bacteria➔Eukaryota for nearly all LCD classes regardless of window size (S2 Fig) or composition threshold (S3 and S4 Figs).

It is also worth noting that broad comparisons between the domains of life are influenced by the uneven distribution of species across taxonomic groups, resulting in some groups more heavily weighting LCD frequency statistics. As a simple illustration, we evaluated the organism-level LCD frequencies for primary LCD classes as a function of the first term (i.e., a clade) following the domain of life in the taxonomic lineages of each reference proteome. For some primary LCD classes, organism-level LCD frequencies differ between clades within a single domain of life (S5–S8 Figs). Therefore, LCD frequency statistics will be affected both by imbalances in real-world taxonomic distributions as well as research bias focusing on specific taxonomic groups [34,35]. Future studies may further parse LCD frequencies with increasing resolution (finer clade levels), and LCD frequency comparisons may need to be updated periodically as our collective proteomic data (i.e., reference proteomes) further expand into understudied taxonomic groups.

### Proteins Containing Specific Types of LCDs are Significantly Associated with Identical Functions Across Organisms

Prior functional characterization of LCDs has been predominantly limited to individual instances of LCDs or exploration of a subset of LCD classes or model organisms. Each level of granularity is important for developing a holistic understanding of LCD frequencies and functions. To gain a broader view of LCD functions within and across the domains of life, we performed gene ontology (GO)-term analysis separately for each LCD class in all organisms with available ontologies (*n* = 294 archaea, *n* = 7533 bacteria, *n* = 2060 eukaryotes, and *n* = 6134 viruses). For any GO term that was significantly enriched for an LCD class in at least one organism (herein referred to as an “LCD class/GO term pair”), the percentage of organisms in the same domain of life that also exhibit significant enrichment for that GO term and LCD class was then calculated, effectively indicating how often that LCD class is associated with the same function across organisms in the same domain of life (see S7 Table for a summary of all statistically significant LCD class/GO term pairs within each domain of life).

**Fig 2:**
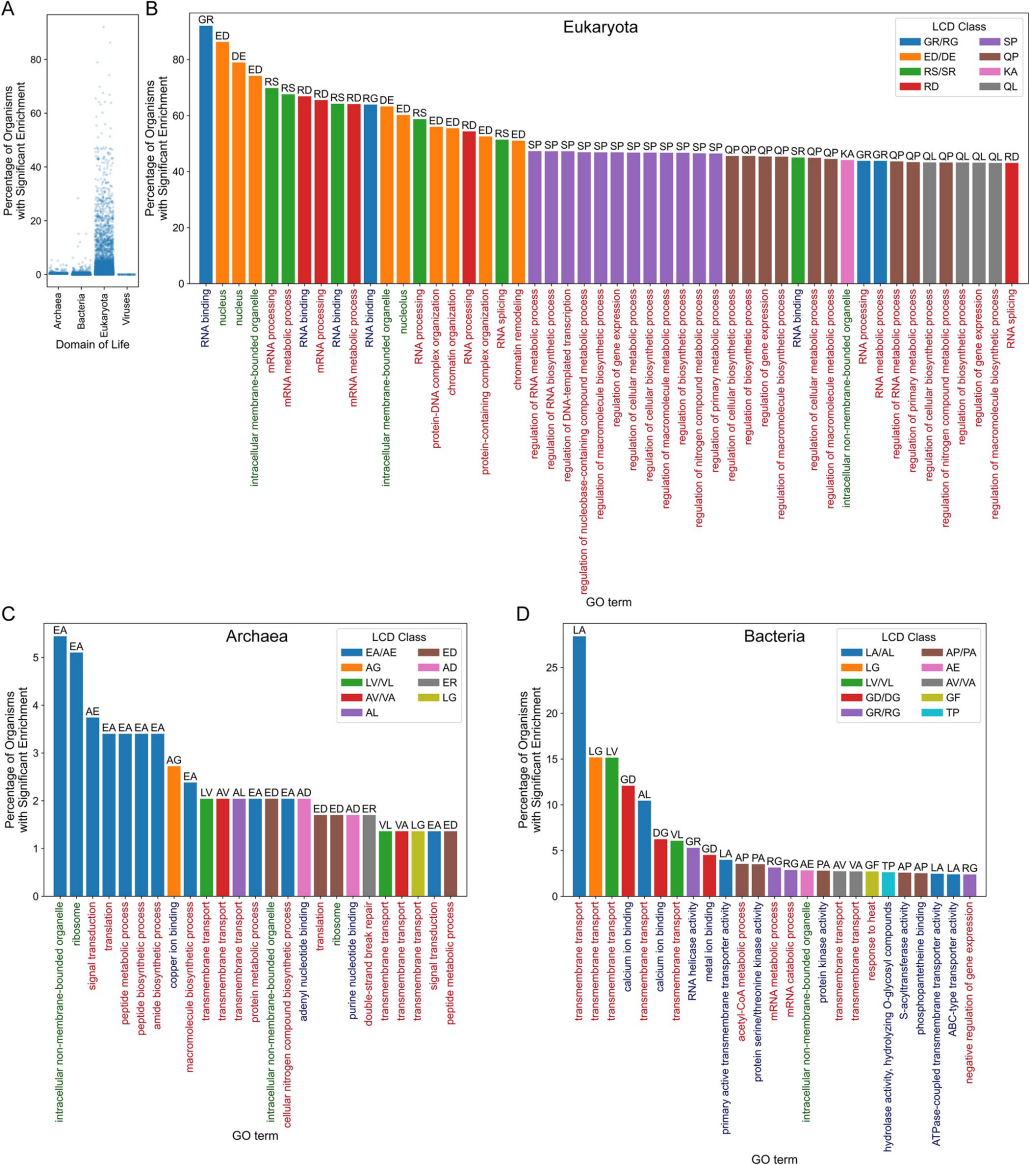
LCD classes most frequently associated with the same function(s) across organisms for each domain of life. (A) For all GO terms significantly associated with an LCD class in at least one organism, the percentage of organisms sharing significant enrichment for that LCD class/GO term pair was calculated separately for each domain of life. Each dot represents a single LCD class/GO term pair. (B) Top 50 LCD class/GO term pairs with respect to the percentage of organisms sharing significant enrichment for that pair in eukaryotes. (C,D) Top 25 LCD class/GO term pairs with respect to the percentage of organisms sharing significant enrichment for that pair in archaea and bacteria, respectively. For panels B-D, bar color corresponds to LCD class, with reciprocal classes (e.g., GR and RG LCDs) assigned the same color for simplicity. GO terms on the *x*-axis are colored according to the GO-term category with Biological Process (BP) in red, Cellular Component (CC) in green, and Molecular Function (MF) in blue. Top-ranking LCD class/GO term pairs for viruses are shown in S9 Fig. For simplicity, only GO terms that were significantly enriched (Šidák-corrected *p* < 0.05) and had a minimum depth of 4 in the gene ontology are shown.

Among eukaryotes, a number of significant LCD class/GO term pairs were shared across a high percentage of organisms (Fig 2A and 2B). In contrast, fewer LCD class/GO term pairs were shared within archaea (Fig 2A and 2C), bacteria (Fig 2A and 2D), or viruses (Figs 2A and S9), likely due to the overall lower frequencies of LCDs in these organisms resulting in lower statistical power in the GO-term analyses. When ranked according to the percentage of organisms sharing the GO term, GR LCDs emerge as the LCD class most consistently linked (in >90% of eukaryotes) to the same function, RNA binding (Fig 2B), which is a well-known function of classically defined RGG domains [36,37]. RG LCDs are also linked to RNA binding in 63.9% of eukaryotes, reflecting the higher prevalence of G in classical RGG domains, but also indicating that reciprocal classes (as defined here) sometimes share the same function (Fig 2B). The RS/SR and RD LCD classes were also significantly associated with functions related to RNA binding and RNA splicing across a high percentage of eukaryotes (Fig 2B), consistent with previous observations [38,39]. Given that R occurs in all of the LCD classes strongly associated with RNA binding and RNA processing, it may play a unique role in RNA-protein interactions that cannot be perfectly compensated for by the other positively charged residues [40]. The DE/ED LCD classes were strongly associated with a variety of terms pertaining to the nucleus, nucleolus, and chromatin organization (Fig 2B). SP LCDs were frequently associated with a variety of gene expression- and transcription-related functions (Fig 2B). Finally, QP and QL LCDs are frequently associated with gene expression and metabolic processes (presumably transcription or translation; Fig 2B).

Although fewer LCD class/GO term pairs were shared within archaea or bacteria, there are discernible trends among those most shared. For example, in archaea, EA/AE and ED LCDs were significantly linked to a variety of terms related to translation, as well as signal transduction for EA/AE LCDs (Fig 2C). In bacteria, a variety of hydrophobic LCD classes, especially those with L as the primary or secondary amino acid, were associated with transmembrane transport in many organisms (Fig 2D). Interestingly, RG/GR LCDs are also linked to RNA helicase activity, RNA metabolism, and gene expression: terms related to those for eukaryotes, but without the strong association observed specifically for the “RNA binding” GO term. Other associations between LCD classes and GO terms in bacteria include GD/DG LCDs linked to calcium binding, AP/PA LCDs linked to acetyl-CoA metabolism and kinase activity, GF LCDs linked to response to heat, and TP LCDs linked to hydrolysis of O-glycosyl compounds (Fig 2D). Statistically significant functional associations with LCD classes were exceptionally rare in viruses (S9 Fig) due both to the paucity of LCDs in viruses and the limited statistical power afforded by small proteome sizes.

These data suggest that LCDs can, at least in some cases, specialize in particular cellular and molecular processes, likely through unique biophysical or structural characteristics. For many of the LCD classes highlighted here, direct involvement of the LCDs in the detected functions has already been demonstrated experimentally, the LCD classes generally have biophysical properties consistent with the functional annotations, and the strong association between LCD classes and the same functions across many organisms is suggestive of a direct role in the indicated functional process. However, an important caveat is that GO-term analyses are based only on statistical associations between the LCD-containing proteins and their corresponding functional categories: they do not necessarily indicate direct involvement of each LCD in the process indicated by functional annotation. Therefore, experimental evidence directly linking the LCDs themselves to the functional process is likely still required for many LCD class/GO term pairs.

### Rare LCDs Have Specific Secondary-Class Preferences and Can Be Linked to Known and New Functional Classes in Well-Studied Organisms

**Fig 3:**
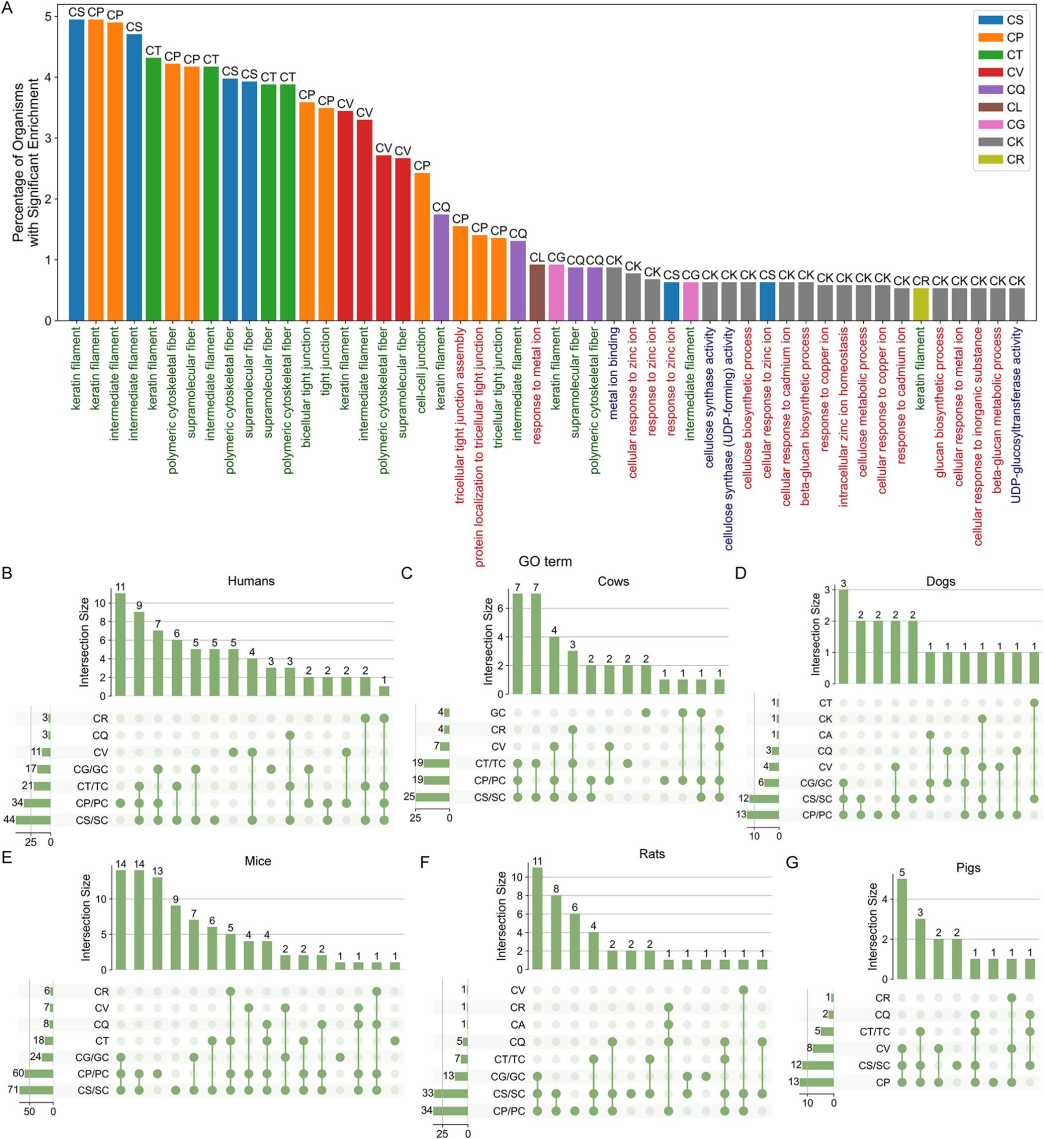
Protein set overlap among keratin-associated proteins identified within C-rich LCD classes in model eukaryotes. (A) Top-ranking enriched GO terms associated with CX LCD classes, sorted by the percentage of eukaryotic organisms with significant GO-term enrichment for the LCD class/GO term pair. Bar color corresponds to LCD class. GO terms on the *x*-axis are colored according to the GO-term category with Biological Process (BP) in red, Cellular Component (CC) in green, and Molecular Function (MF) in blue. Only GO terms that were significantly enriched (Šidák-corrected *p* < 0.05) and had a minimum depth of 4 in the gene ontology are shown. (B-G) UpSet plots (analogous to a Venn diagram) depict the co-occurrence of CX LCDs among keratin-associated proteins for humans (B), cows (C), dogs (D), mice (E), rats (F), and pigs (G). Keratin-associated proteins with C-rich LCDs were parsed into secondary LCD classes and evaluated for co-occurrence (i.e., two LCD types appearing in the same protein) across LCD classes. Each pair of reciprocal LCD classes (e.g., CS and SC) was grouped into a single representative category. The graphs on the left of each panel indicate the number of keratin proteins containing each secondary class of C-rich LCDs. The bar graph at the top of each panel indicates the number of proteins with each combination of C-rich secondary LCD classes (which are indicated by green dots and connecting lines below the bar graph). For example, in humans, three keratin proteins contain CR LCDs: two of these proteins also contain CT/TC and CS/SC LCDs, while one contains CT/TC, CP/PC, and CS/SC LCDs.

The primary class of C-rich LCDs was relatively rare among archaea, bacteria, and viruses but was quite common among eukaryotes (Fig 1A). Further examination of secondary LCD classes among the C-rich LCDs indicates disproportionate sorting of C-rich primary LCDs into specific secondary LCD classes (Fig 1B), potentially reflecting functional specialization among certain C-rich secondary LCD classes. When GO-term results for LCDs in eukaryotes (S7 Table) are filtered to include only those corresponding to CX LCDs (i.e., secondary LCD classes with C as the primary residue), a variety of CX classes including CS, CP, CT, CV, CQ, CG and CR are associated with keratin and keratin-related functions across ~0.5%-5% of eukaryotes (Fig 3A). Furthermore, multiple XC LCD classes (reciprocal LCD classes with C as the secondary residue) are also associated with keratin-related functions (S10A Fig), as well as some classes associated with non-keratin-related functions (discussed below). This is both consistent with the known compositional bias of keratin proteins and partially explains why C-rich LCDs are more frequently observed in eukaryotes compared to other domains of life. Indeed, when only mammals are considered, the percentage of organisms sharing keratin/keratin-related functions associated with CX LCDs rises to ~15%-80% depending on the class (S10B Fig), providing both an example of highly specific LCD functions in niche organisms and an illustration of how specific taxonomic groups can contribute disproportionately to the global domain statistics.

Coarse-grained analysis of whole-protein amino acid composition suggested that keratin proteins with distinct biological purposes and keratin proteins from different organisms exhibit unique compositional profiles [41]. The hierarchical classification of LCDs afforded by LCD-Composer enables finer resolution of the LCD regions predominantly responsible for the C bias and enables spatial resolution of distinct C-rich secondary LCD classes within single proteins. To explore C-rich LCDs in greater depth, we selected 13 model eukaryotes for further evaluation, including both mammalian and non-mammalian eukaryotes (see Methods).

To determine the degree to which the C-rich keratin-related proteins overlapped across LCD classes within each model organism, proteins that were annotated with the terms “keratin filament” or “keratinization” were collected for all of the C-rich LCD classes, including both CX and XC classes (S8 Table). As expected, C-rich keratins appear only in a subset of the model organisms. LCD types and frequencies among keratin-associated proteins differed substantially across organisms (Fig 3B-3G). For example, human keratin-associated proteins contained the most diversified set of LCDs (Fig 3B), while mouse and rat keratin-associated proteins were more strongly skewed toward CS/SC or CP/PC LCDs (Fig 3E and 3F), and cow keratin-associated proteins tended to contain a mixture of CS/SC, CP/PC, and CT LCDs (Fig 3C). Additionally, the types of LCDs occurring in the same proteins (“co-occurring” LCDs) varied across organisms. For example, the human CG/GC and CV classes exclusively co-occurred with the CS/SC and CP/PC classes, whereas the human CT/TC class co-occurred with the CQ and CR classes in addition to the CS/SC and CP/PC classes (Fig 3B).

Given the constraints used to define secondary LCD classes (≥40% of a primary amino acid and ≥20% of a secondary amino acid), it is possible that the same LCD sequence could simply be assigned to multiple LCD classes. However, barring a few exceptions, the LCDs of different classes occupied spatially distinct regions in each keratin-associated protein for all organisms (S11–S13 Figs). Therefore, not only are C-rich LCDs a largely eukaryote-specific class with corresponding eukaryotic functions, but these C-rich LCDs can be spatially resolved within individual proteins by defining compositional signatures.

While the keratin proteins serve as a robust, prototypical model of C-rich LCDs, a variety of non-keratin functions are also detected for specific secondary LCD classes in particular organisms. For example, CK LCDs preferentially occur among metallothionein proteins and are significantly associated with functions related to metal ion homeostasis in humans, mice, and dogs. These metallothioneins predominantly bind divalent metals such as zinc, copper, and cadmium via their C residues [42], though their K residues may also play an auxiliary role in metal binding [43,44], structural stability [45], subcellular localization [46], and regulation of steady state metallothionein levels [47]. Intriguingly, the reciprocal class, KC LCDs, were strongly associated with an entirely distinct set of functions related to synaptic vesicle regulation and neurotransmitter release across a variety of organisms (S7 Table), highlighting the functional specificity that can be observed even among highly similar LCD classes. Indeed, when reciprocal XC LCD classes are included in the analysis of shared functions across eukaryotes, the links between KC LCDs and synaptic functions are shared by a much higher percentage of eukaryotic organisms (>20%) compared to the links between CX LCDs and keratin associated functions (S10A Fig and S7 Table).

Although the CG and CP classes were associated with keratins in mammals, these classes (as well as the reciprocal GC LCDs) were instead associated with spermatogenesis related functions in fruit flies (S7 Table). The QC LCD class in roundworms (*C*. *elegans*) was associated with functions related to the unfolded protein response and general stress responses in the ER, as well as pharynx development (S7 Table). Indeed, all of these proteins are members of the *abu* family of proteins, which were identified as ER-localized transmembrane proteins that are upregulated in response to ER stress and improved viability during stress [48]. Based on their homology with the CED-1 mammalian ER receptor protein, it was proposed that the *abu* proteins likely act as receptors for damaged macromolecules, including oxidized lipoproteins: it is tempting to speculate that these domains could play a role in sensing or responding to reactive oxygen species–which are generated during protein folding in the ER–or oxidized proteins. Additionally, these *abu* proteins are upregulated during pharyngeal cuticle development and are secreted into the pharyngeal cuticle, where they have been proposed to play a role in cuticle formation and maintenance via phase separation mediated, at least in-part, by C residues in the QC domains (along with other C-rich cuticle proteins) [49]. Other notable, non-keratin-related functions of CX/XC classes include EC LCDs associated with metallopeptidase activity and “sperm head plasma membrane” in cows; IC LCDs associated with natural killer cell receptor binding in rats; LC LCDs associated with hemidesmosomes and cell-substrate junctions in roundworms; PC LCDs associated with response to estradiol in mice; and CS LCDs also linked to metal ion binding, regulation, and signaling in various organisms (S7 Table).

Gene duplication has been proposed to play a major role in some protein families, including keratin proteins [50], which might contribute both to the prevalence of certain types of LCDs as well as the functions associated with the LCD-containing proteins. Indeed, when Pfam clan annotations are mapped to LCD-containing proteins from each class, a high percentage of proteins from C-rich LCD classes are associated with a single Pfam clan specifically in eukaryotes (S14 Fig). However, low percentages of proteins from nearly all other LCD classes are observed across organisms, suggesting that, in general, the prevalence of most types of LCDs is not due predominantly to gene duplication among specific protein families.

In summary, examination of organism-level LCD class frequencies can be used to identify rare LCD classes, reveal specific preferences for secondary amino acids among rare LCD classes, define eukaryote-specific LCD classes (and potentially LCD classes specific to other domains of life), infer functions of multiple rare LCD classes, and spatially resolve similar types of secondary LCDs within families of related proteins.

### Organisms Containing a Large Number of Proteins with Rare and Eukaryote-Specific LCDs Highlight Widespread LCD-Associated Functions

**Fig 4:**
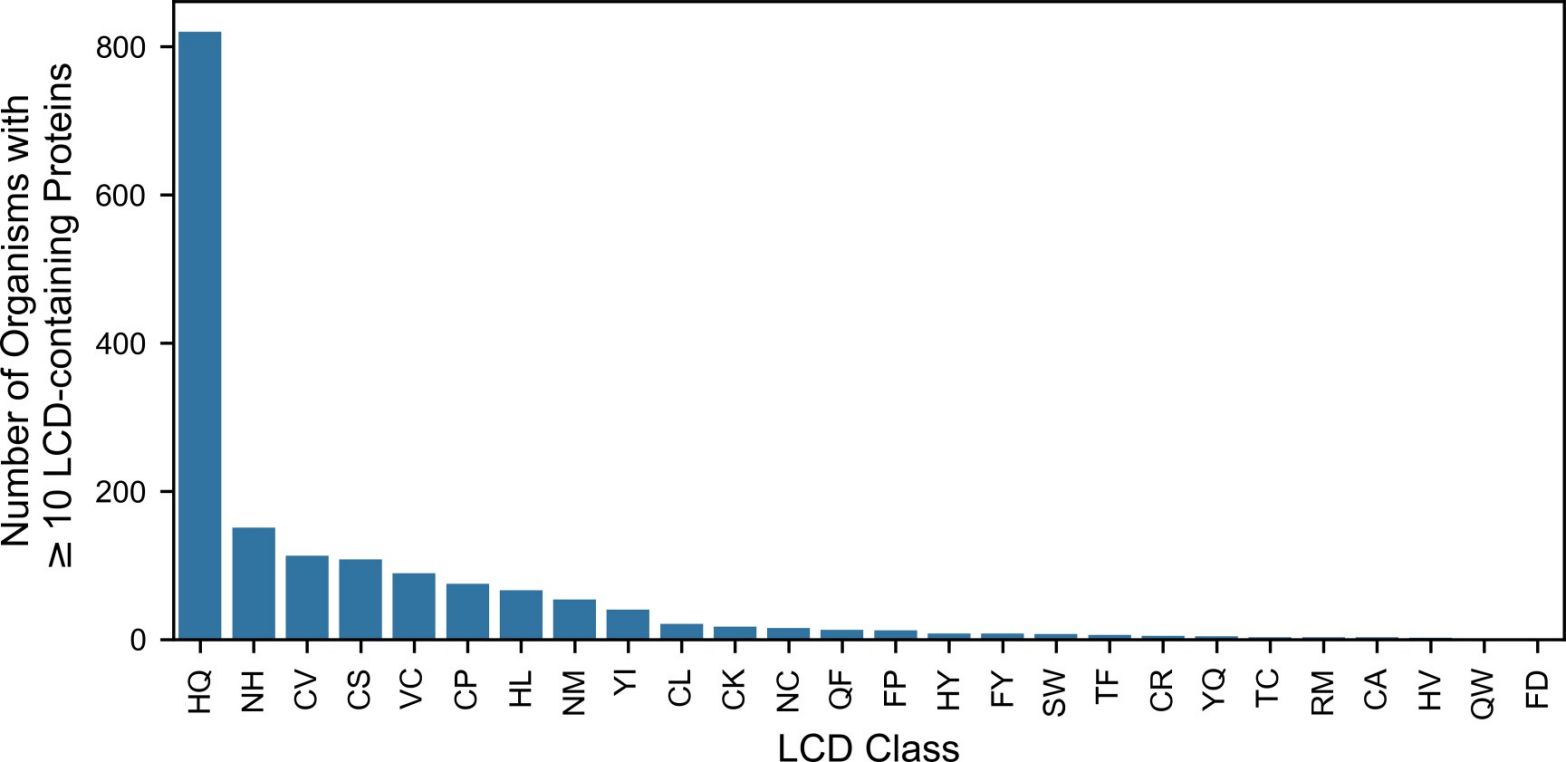
Frequencies of top eukaryote-specific LCD classes. Bar plot indicating the number of organisms with ≥10 LCD-containing proteins for each eukaryote-specific LCD class.

In light of the functional specificity observed among C-rich LCDs in eukaryotes, we next explored whether other eukaryote-specific LCD classes were represented in high numbers across a large number of organisms. Eukaryote-specific LCD classes were arbitrarily defined as those found in >15% of eukaryotic proteomes but <2% of the proteomes from archaea, bacteria, and viruses (each evaluated independently). Among these classes, HQ LCDs were the most common in eukaryotes, with >800 organisms containing ≥10 proteins with HQ LCDs (Fig 4 and S1 Table). Importantly, HQ LCDs also occupy a larger percentage of the proteome, on average, in eukaryotes (0.0048%) compared to archaea, bacteria, and viruses (0.000034%, 0.000036%, and 0.00040%, respectively), indicating that HQ LCDs are not more common in eukaryotes due simply to their larger proteomes. NH LCDs are the next most common with ~150 organisms having 10 or more LCD-containing proteins, followed closely by the previously examined CV, CS, VC, and CP LCDs (Fig 4). Finally, >50 eukaryotic organisms also had 10 or more proteins from the HL and NM LCD classes.

**Fig 5:**
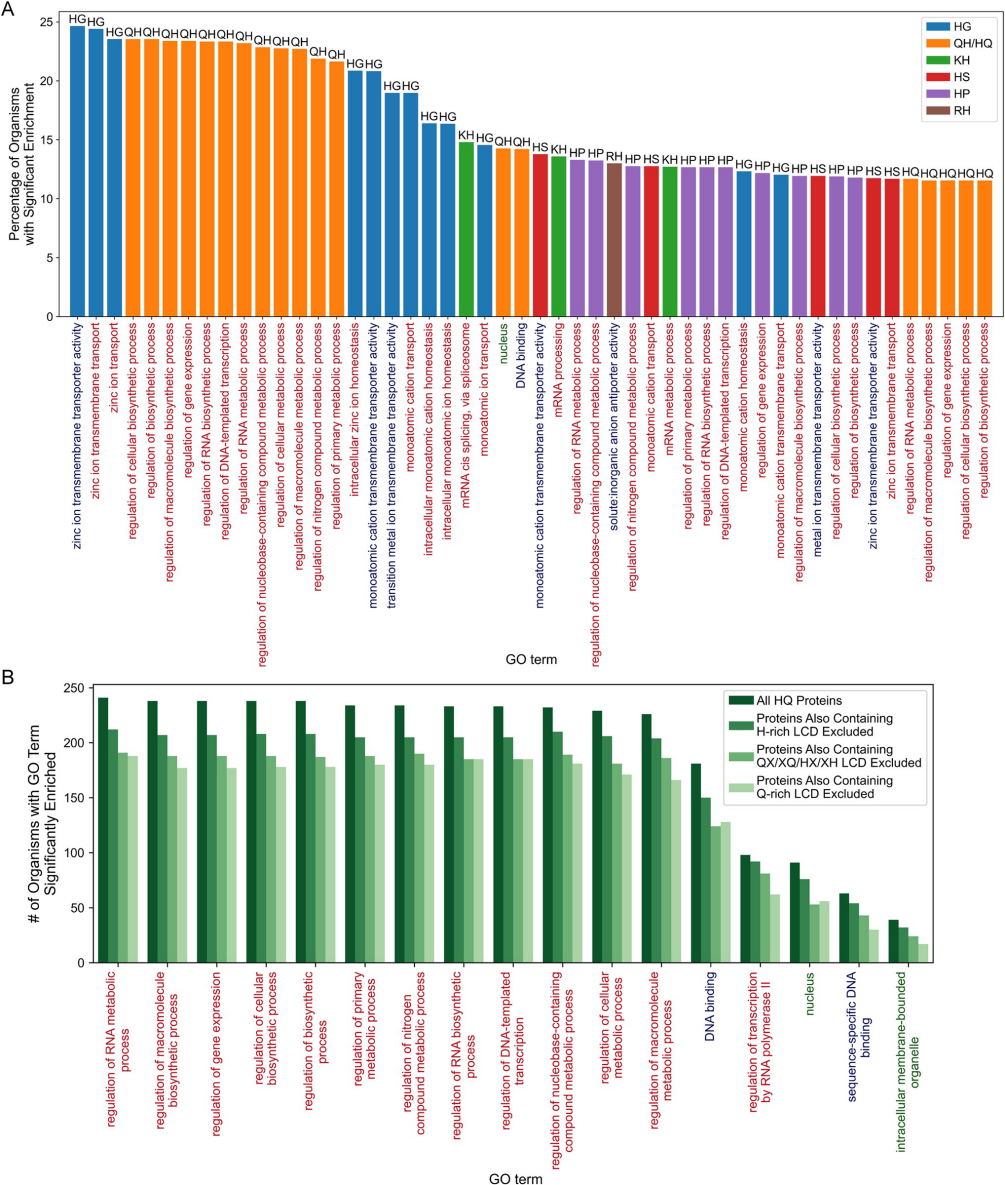
Functions consistently enriched for proteins containing H-rich LCDs in eukaryotes. (A) For all HX and XH LCD classes (where X represents any amino acid except histidine), the percentage of eukaryotic organisms with significant enrichment for each LCD class/GO term pair was calculated. The bar plot indicates the top 50 LCD class/GO term pairs with the highest percentage of eukaryotes exhibiting significant enrichment among the HX or XH LCD classes. Bars are colored according to LCD class (with the reciprocal classes QH and HQ assigned the same color), whereas GO terms are colored according to the GO-term category with Biological Process (BP) in red, Cellular Component (CC) in green, and Molecular Function (MF) in blue. Only GO terms that were significantly enriched (Šidák-corrected *p* < 0.05) and had a minimum depth of 4 in the gene ontology are shown. (B) Frequency of significant enrichment across organisms for each GO term associated with proteins containing HQ LCDs. GO-term analyses were also performed on the same set of proteins but with those that also contained a spatially distinct H-rich LCD (primary class), Q-rich LCD (primary class), or QX, XQ, HX, and XH LCD (where X is any residue other than Q or H) removed prior to analysis. For simplicity, only GO terms that were significantly enriched for ≥30 organisms and had a minimum depth of 4 in the gene ontology are shown.

Excluding the C-rich LCD classes (which were examined in the previous section), H serves as the primary or secondary residue in 3 of the 4 well-represented, eukaryote-specific LCD classes. To identify functions associated with H-rich LCDs across eukaryotes, we extracted GO term results specifically associated with HX or XH LCDs and sorted the LCD class/GO term pairs based on the percentage of eukaryotes sharing significant enrichment of that pair (Fig 5A). HG LCDs were strongly linked to transmembrane transport of zinc (and potentially other cations), though this was not classified as a eukaryote-specific LCD class by our criteria. Further supporting this notion, transmembrane transport of zinc/cations associated with HG LCDs (as well as HD LCDs) were overwhelmingly the top LCD class/GO term pairs among bacterial H-rich LCDs as well (S15 Fig), albeit with a much smaller percentage of bacterial organisms sharing these functional links. Both QH and HQ LCDs are among the next highest-ranking LCD classes with shared functions in eukaryotes and are linked to gene expression and transcription (Fig 5A and S7 Table). This is consistent with a previous study of 13 metazoan proteomes, where Q/H-rich LCDs were found to be significantly associated with transcription-related functions in a small subset of representative organisms [11]. These functions remain significantly enriched in most organisms even when proteins with spatially distinct Q-rich primary LCDs, H-rich primary LCDs, or QX, XQ, HX, and HX secondary LCDs are removed prior to GO-term analysis (Fig 5B and S9 Table), suggesting at least some level of specificity for HQ LCDs. Furthermore, consistent with the classification of HQ LCDs as eukaryote-specific LCDs, HX and XH classes (as well as the H primary LCD class) are not associated with gene expression or transcription-related terms in any archaeal, bacterial, or viral organism (S7 Table). These data indicate that the association between QH/HQ LCDs and transcription extends to a large percentage of eukaryotes and is eukaryote-specific. Finally, additional H-rich LCD classes with functions shared by a high percentage of eukaryotes include HP LCDs associated with transcription; HS LCDs associated with transmembrane transport of zinc/cations; and KH LCDs associated with mRNA splicing (Fig 5A).

When examined individually, NH, HL, and NM LCDs–which are the next highest-ranking, eukaryote-specific LCD classes in Fig 4 –are also associated with transcription related functions but to a much smaller extent compared to QH/HQ LCDs (<1% of eukaryotes sharing the same functions; S16 Fig and S7 Table). In addition, these classes were sometimes associated with non-transcription-related functions in specific organisms. For example, proteins with NM LCDs are significantly associated with acetyltransferase activity in only two organisms (*Periconia macrospinosa* and *Phytophthora infestans*), whereas the same class is associated with modulating host organism defense pathways in the plant pathogen, *Albugo candida* (S7 Table). A broader diversity of functions for proteins with NH LCDs are observed and appear to be organism-specific. For example, proteins with NH LCDs are significantly associated with ubiquitin-protein transferase activity in *Tetranychus urticae*, spermine/spermidine biosynthesis in *Aedes albopictus*, response to nutrient deprivation in *D*. *discoideum*, and histone H3 methyltransferase activity in *Meloidogyne enterolobii* (S7 Table).

Together, these examples highlight both functional overlap and functional specificity that can be observed across secondary LCD classes, as well as identify eukaryote-specific LCD classes.

### Protein-level LCD Class Frequencies: LCDs Are More Common than Expected from Whole-Proteome Amino Acid Frequencies and Exhibit Domain-Specific Enrichment of LCD Classes

Organism-level LCD frequencies indicate which organisms contain at least one instance of an LCD but do not indicate how prevalent the LCDs are within each proteome. At the protein level, LCD frequency can be defined as the number or percentage of proteins containing an LCD within a single organism. However, key factors influencing protein-level LCD frequencies are whole-proteome amino acid frequencies and proteome size, both of which can vary substantially between organisms. Common amino acids might be expected to result in higher LCD frequencies, since encountering sufficient clusters of these amino acids is statistically more probable. A prototypical example is the *P*. *falciparum* proteome [51], which is remarkably N-rich and has a correspondingly high frequency of N-rich LCDs. As noted above, an abundance of particular types of LCDs may necessitate specific adaptations to cope with (or even leverage) those LCDs regardless of their underlying amino acid frequencies. Additionally, LCDs contribute to whole-proteome amino acid abundance, which could lead to underestimates of LCD enrichment since, in the absence of LCDs, the amino acid may be less abundant in the proteome. Nevertheless, examination of LCD frequencies in the context of whole-proteome amino acid frequencies provides a complementary view by indicating LCD classes that are more frequent than expected given the abundance of their constituent amino acids and the total number of proteins in the proteome.

To determine how often protein-level LCD frequencies exceed expectations based on background amino acid frequencies and proteome size, each proteome was scrambled and re-evaluated for all LCD classes using LCD-Composer (see Methods). For each LCD class, LCD frequencies for the scrambled proteomes were then compared to corresponding LCD frequencies in the original proteome. Finally, for each LCD class, the percentage of organisms for which that LCD class was overrepresented relative to its scrambled frequency was calculated separately for each domain of life.

**Fig 6:**
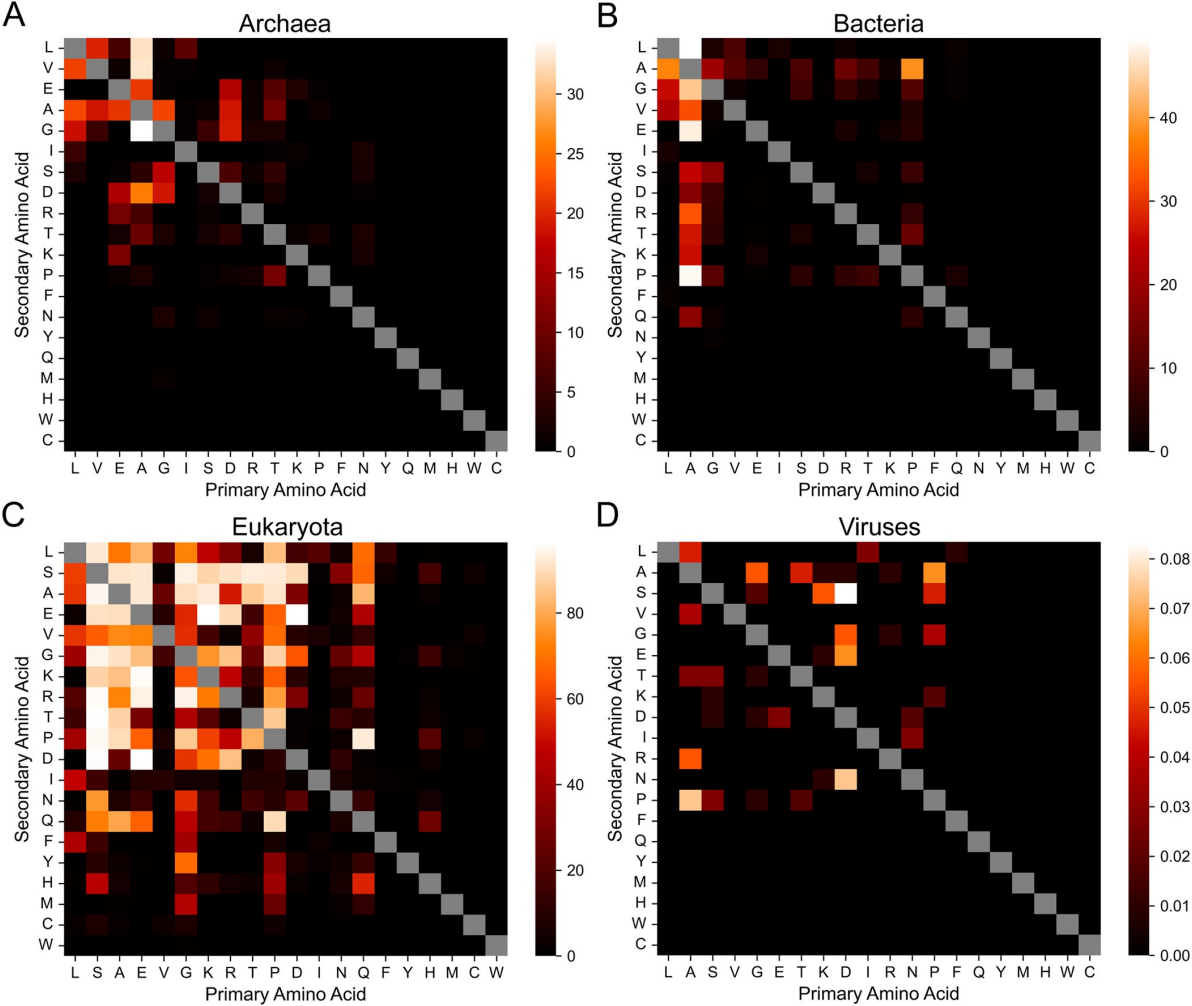
Percentage of organisms with significantly enriched LCDs after accounting for amino acid frequencies. The percentage of organisms with significantly enriched LCD-containing proteins (relative to a scrambled version of each proteome) is depicted for each LCD class in archaea (A), bacteria (B), eukaryota (C), and viruses (D). LCD frequencies in the original and scrambled proteomes were compared using Fisher’s exact test for each LCD class in each organism. Within each organism, *p*-values for all represented LCD classes (i.e., those with at least one LCD instance in the original or scrambled proteomes) were corrected using the Holm–Šidák correction method to account for multiple hypothesis testing. Significant enrichment is defined as *p* < 0.05 after multiple-test correction.

Relatively few LCD classes were significantly enriched across a high percentage of archaea or bacteria (Fig 6A and 6B; supplementary table available at [33]), which is due in part to the rarity of many LCD classes in these domains of life. Multiple LCD classes with A as the primary or secondary amino acid were significantly enriched in a reasonably large percentage of both archaea and bacteria (Fig 6A and 6B). In addition to these classes, the LA, LG, LV, GA, and GS classes are enriched in a large percentage of organisms in both archaea and bacteria (Fig 6A and 6B). Differences between archaea and bacteria are also apparent though: multiple D-rich LCD classes are enriched in a high percentage of archaea but not bacteria, and multiple P-rich LCD classes are enriched in many bacteria but not archaea (Fig 6A and 6B). While overall percentages of viruses with LCDs are low for all LCD classes (Fig 6D), the highest values are achieved for multiple D-rich, A-rich, and P-rich LCD classes, along with an assortment of unrelated secondary LCD classes. However, it is worth noting that a large percentage of archaeal and bacterial organisms exhibit at least some degree of LCD enrichment (regardless of statistical significance) for a variety of LCD classes (S17 Fig). In contrast, for all domains of life, relatively few organisms exhibit LCD depletion (i.e., LCDs that are less common in the original proteome compared to the scrambled proteome; S17 Fig), and statistically significant LCD depletion was extremely rare (S18 Fig).

In contrast to the other domains of life, eukaryotes exhibit both the highest percentages of organisms with significant LCD enrichment and the greatest variety of LCD classes achieving a high percentage (Fig 6C). High percentages tend to cluster among the 11 most common amino acids in eukaryotes, indicating that significant LCD enrichment typically occurs in spite of high background amino acid frequencies. However, amino acid frequency does not always determine LCD enrichment: L and V are ranked 1^st^ and 5^th^, respectively, in terms of amino acid abundance but their corresponding LCD classes are rarely enriched in eukaryotic organisms (Fig 6C). Among the remaining 9 most abundant amino acids, specific secondary classes are enriched. For example, GR and RG LCD classes are both significantly enriched across a high percentage of organisms (94.1% and 85.0%, respectively; Fig 6C) while GK and KG LCD classes are significantly enriched in fewer organisms (63.8% and 76.5%, respectively; Fig 6C), consistent with the functional importance and specificity of RGG domains [36]. However, enrichment of reciprocal classes is not always strong: GF and GY LCDs are significantly enriched in 40.8% and 68.4% of eukaryotes, respectively, yet significant enrichment of FG or YG is extremely rare (0.2% and 1.7%, respectively; Fig 6C). This asymmetry aligns with the sequence features of “F/YGG-motifs”–which tend to have more G than F or Y–and the known role of F/YGG-motifs in RNA binding [39,52]. Similarly, QL LCDs are significantly enriched in 68.9% of eukaryotes, but LQ LCDs are enriched in only 9.1% of eukaryotes despite L being the most abundant amino acid, on average, in eukaryotic proteomes (Fig 6C).

LCD classes composed of physicochemically similar amino acids are also not always similarly enriched across organisms and exhibit high context dependence. For example, RE and KE LCDs are frequently enriched in many eukaryotes (89.4% and 96.3%, respectively; Fig 6C), whereas RD and especially KD LCDs are less-frequently enriched (84.6% and 69.0%, respectively; Fig 6C). This difference may be due in part to the apparent compositional and structural preferences of charged single α-helices, where D may not be an effective substitute for E in this specific subclass of LCDs [53,54]. However, ED and DE LCDs are among the most enriched LCD classes (96.5% and 96.2%, respectively; Fig 6C) and can play roles in regulating gene expression via nucleic acid mimicry [55,56], suggesting that these residues may be largely interchangeable, at least in some highly anionic LCDs. Conversely, KR and RK LCDs are less frequently enriched and exhibit a large difference in frequency of enrichment (71.5% and 47.8%, respectively; Fig 6C), suggesting both that highly cationic LCDs are less common compared to highly anionic LCDs and that K and R are not interchangeable in this context.

Among the 9 least abundant amino acids in eukaryotes, significant enrichment of LCD classes across a high percentage of organisms is less common. Nevertheless, multiple QX LCD classes (e.g., QP, QA, QL, and QS) are significantly enriched in relatively high percentages of eukaryotes. Among H-rich LCDs, HQ LCDs are enriched in the highest percentage of eukaryotes (28.1%; Fig 6C) despite the low overall proteome abundance of H, further supporting a functional role for this LCD class (Fig 5). Additional HX LCDs significantly enriched in certain eukaryotes (Fig 6C) include HP LCDs (22.0%), HS LCDs (17.8%), and HG LCDs (15.9%), all of which are among the top-ranking HX LCDs with shared functions across eukaryotes (Fig 5A), corroborating their functional importance as well. Finally, NS LCDs (33.6%), NG LCDs (25.9%), and–to a lesser extent–NT LCDs (14.6%) and ND LCDs (12.2%) are also enriched in a large fraction of eukaryotes.

**Fig 7:**
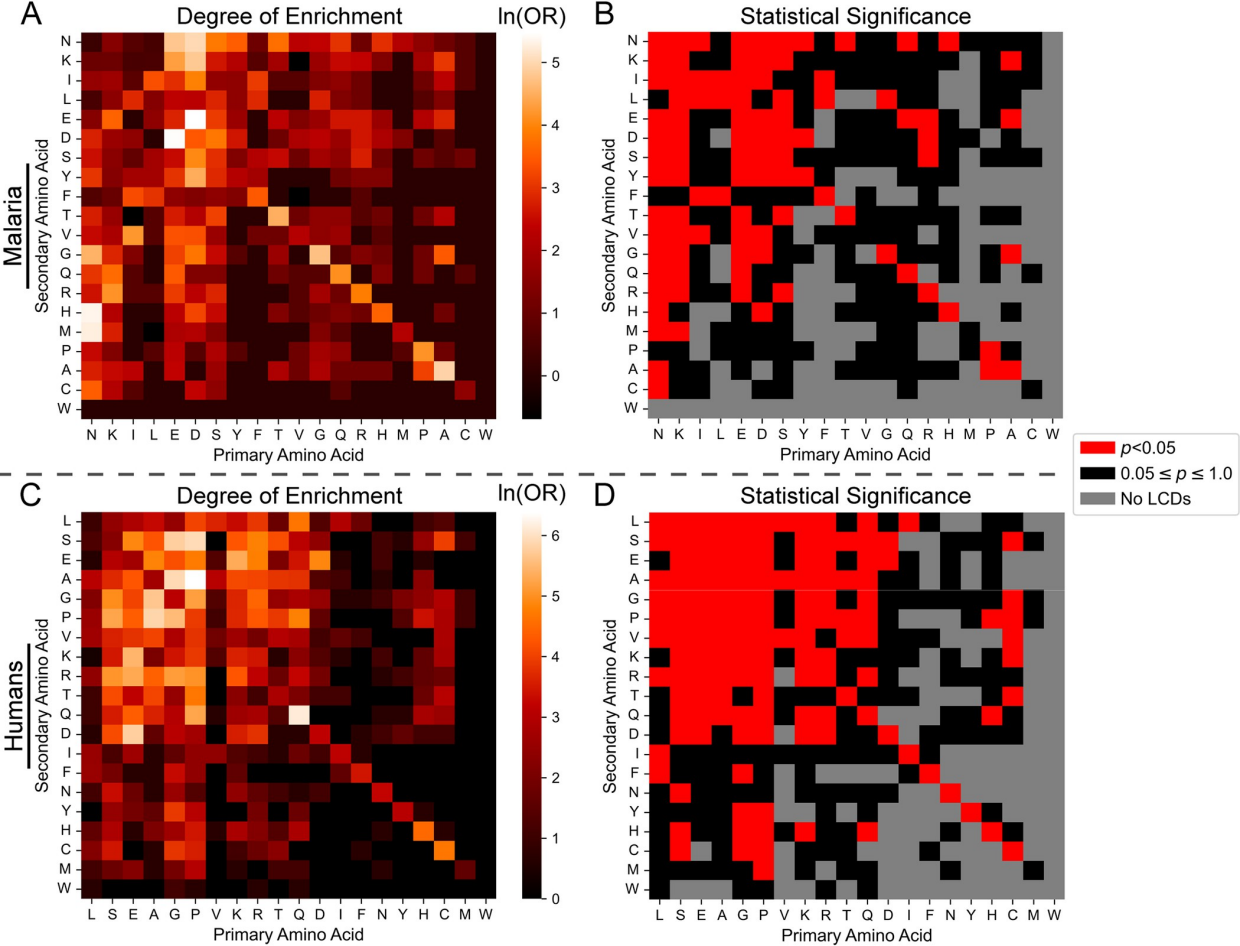
Statistical LCD enrichment by LCD class in the malarial and human proteomes. (A) Heatmap depicting the degrees of LCD enrichment (expressed as the lnOR) for each LCD class among the *P*. *falciparum* proteome (UniProt ID: UP000001450_36329). For LCD classes in which the number of LCDs in either the original or scrambled proteomes were 0, a value of 1 was added to all cells in the contingency table to calculate a biased lnOR (see Methods). (B) Binary classification for LCD categories for which enrichment was statistically significant (red squares) or statistically non-significant (black squares) after multiple-test correction. Grey squares indicate LCD categories that were excluded from statistical analysis since no LCDs were found in both the original and scrambled proteome. (C) Degrees of LCD enrichment in the human proteome (UniProt ID: UP000005640_9606). (D) Statistical significance for LCD enrichment in the human proteome. For all panels, the diagonals represent corresponding values for each primary LCD class. The data underlying these heatmaps can be found in the supplementary data available at [33].

It is important to note that statistical power in these experiments is limited both by LCD sample sizes (particularly for rare LCD classes, which have relatively large confidence intervals and high *p*-values) and by multiple-hypothesis test correction (since up to 400 tests are run for each organism). To examine whether the observed differences in LCD class enrichment were driven predominantly by underlying differences in statistical power, the typical degree of enrichment for each LCD class was estimated as the median natural logarithm of the odds ratio (lnOR) across organisms (see Methods). Importantly, the median lnORs for the LCD classes composed of common amino acids are also higher compared to the median lnORs for LCD classes composed of less-common amino acids (S19 Fig; supplementary data available at [33]), suggesting that differences in the percentage of organisms with significant enrichment across LCD classes is not primarily driven by differences in statistical power (though it is clearly still a contributor). This method can also be applied to individual organisms: the malarial parasite proteome–a prototypical proteome with highly skewed amino acid frequencies–exhibits significant enrichment across a variety of N-rich LCD categories despite the unusually high N frequency within its proteome (Fig 7A and 7B). Similarly, E and D are the 5^th^ and 6^th^ most common amino acids, respectively, yet a variety of E-rich and D-rich LCD classes are among the most strongly enriched in the malarial proteome (Fig 7A and 7B)–classes that are understudied in *P*. *falciparum* compared to N-rich LCDs. For example, the native malarial proteome contains 228 proteins with at least one DE LCD (4.2% of the proteome), but none are retained after scrambling. In humans, with the exception of L and V, the LCD categories corresponding to the most frequent amino acids exhibit significant enrichment across a variety of secondary LCD classes (Fig 7C and 7D). Additionally, although whole proteome frequencies for H and C are relatively low in humans and power is generally lower for these LCD classes, multiple LCD classes are significantly enriched in the proteome (Fig 7C and 7D). Thus, LCD instances are not solely attributable to amino acid frequencies, even in organisms with highly biased background amino acid frequencies.

In summary, domains of life exhibit both shared and unique enrichment for specific LCD classes. Eukaryotes exhibit the greatest breadth and most consistent enrichment for a variety of LCD classes, with many enriched classes corresponding to those with known (e.g., GF, YG, GR/RG, DE/ED) or new (e.g., HQ, HG, KH) functional significance. Importantly, LCD classes almost universally exhibit degrees of enrichment that are greater than or equal to those expected based on whole-proteome amino acid frequencies, suggesting that they are often not simply byproducts of amino acid frequencies.

### Per-residue-level LCD Class Frequencies: Select Organisms Achieve the Highest Per-Residue Occupancy for Multiple LCD Classes

The measure of total proteins with LCDs for each LCD class in each organism identifies proteomes with an unusually large number of LCD-containing proteins and aids in determining putative LCD class functions. While this statistic provides an enlightening and useful view of extreme LCD content, it does not account for multiple LCDs within a single protein or the lengths of LCDs within each protein, and organisms with large proteomes are often selectively enriched among the top-ranking organisms. A complementary measure of LCD content is the per-residue occupancy of LCDs, defined as the percentage of all residues in each proteome that are located within LCDs for each class. This statistic offers a slightly different view of whole-proteome LCD content by effectively estimating the percentage of proteome space devoted to each LCD class within each organism.

**Fig 8:**
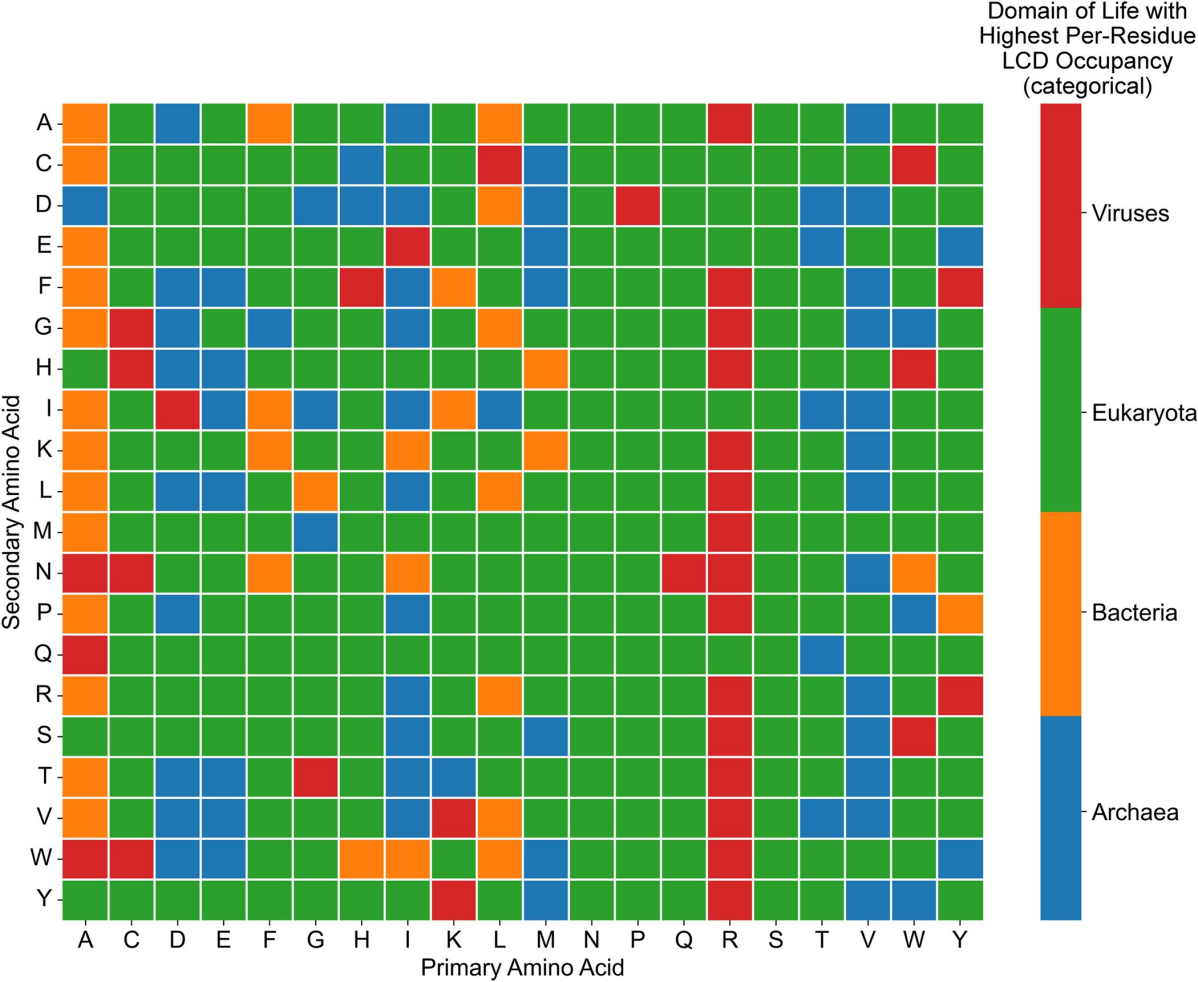
Domains of life with the highest mean per-residue occupancy for each LCD class. Mean per-residue LCD occupancy was calculated for each LCD class within each domain of life. For each LCD class, mean per-residue LCD occupancy values were compared across the four domains of life to determine the domain with the highest per-residue occupancy. The color of each square in the heatmap indicates the domain of life with the highest mean per-residue LCD occupancy. LCD classes on the diagonal represent the primary LCD classes.

Although eukaryotes exhibit the highest organism-level LCD frequencies for the vast majority of LCD classes (Figs 1 and S2–S4), it was not immediately clear if this would be true after accounting for proteome size. Per-residue occupancy values were calculated for all 400 LCD classes (20 primary classes and 380 secondary classes). For each class, the per-residue occupancies were averaged within each domain of life. Mean per-residue occupancies were then compared across the domains of life to determine the domain of life with the highest per-residue occupancy for each LCD class (Fig 8 and S10 Table). Eukaryotes still dominate the majority of LCD classes, suggesting that higher organism-level LCD frequencies are not simply a function of larger proteomes. However, notable exceptions are also apparent. For example, bacteria have the highest per-residue occupancy for 13 of the 20 AX LCD classes (Fig 8), and this is relatively insensitive to window size and composition thresholds in our randomly selected subset of organisms (S20 Fig). Archaea also have the highest per-residue occupancy for many of the DX, EX, IX, and VX LCD classes (Fig 8). Curiously, even viruses can dominate a class, exhibiting the highest per-residue occupancy for the majority of RX LCD classes (Fig 8), consistent with the prevalence of arginine-rich “R-arms” in nucleic acid-binding and capsid proteins in a number of distinct virus families [57]. However, the mean per-residue occupancy for these LCD classes in archaea and viruses may depend more strongly on an uneven distribution of LCD content within the domain of life as these patterns were less robust to changes in LCD search parameters in our randomly sampled subset of organisms (S20 Fig).

**Fig 9:**
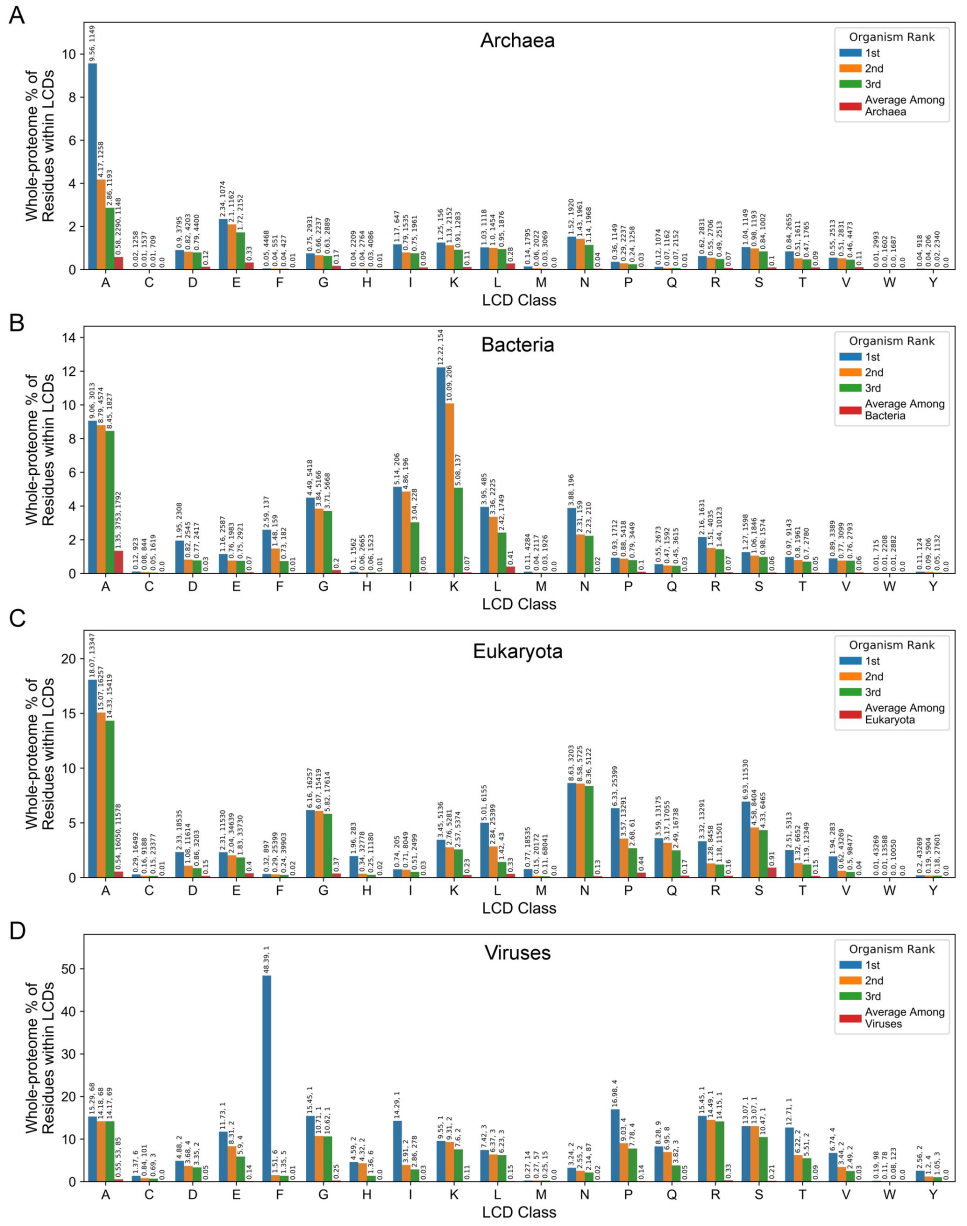
Per-residue occupancy for the top-ranking organisms from each domain of life for the primary LCD classes. Per-residue occupancy was calculated separately for each organism as the percentage of total residues in the proteome that were occupied by LCDs from each primary LCD class for archaea (A), bacteria (B), eukaryota (C), and viruses (D). Values above each bar represent the per-residue occupancy value (as a percentage), followed by the total number of proteins in the corresponding organism. For the average among each domain of life (red bars), the mean number of proteins per organism and the standard deviation in the number of proteins per organism is expressed above the bar for the “A” LCD class only since these values are independent of LCD class.

Next, we identified organisms with the most extreme per-residue occupancy for each LCD class. For many primary LCD classes, per-residue occupancy often reached single-digit or even double-digit percentages for the top three organisms from all domains of life and were sometimes orders of magnitude higher than the mean per-residue occupancy across all proteomes within the corresponding domain of life (Fig 9, S11 Table). Some LCD classes have high per-residue occupancy values among the top-scoring organisms from all four domains of life, while certain domains of life exhibit uniquely high maximum per-residue occupancies for specific LCD classes. For example, the top-ranking organisms achieve relatively high per-residue occupancies for A-rich LCDs across all domains (Fig 9). The highest per-residue occupancy for G-rich LCDs is similar for bacteria and eukaryotes (Fig 9B and 9C) but low among archaea (Fig 9A). Eukaryotes exhibit uniquely high per-residue occupancies for N-rich, P-rich, Q-rich, and S-rich LCDs (Fig 9C) compared to archaea and bacteria (Fig 9A and 9B). For most of these classes, higher per-residue occupancy is achieved despite larger proteomes than are typical for archaea and bacteria (3203 proteins, 25399 proteins, 7635 proteins, and 11530 proteins for the top-ranking eukaryotic proteome in the N-rich, P-rich, Q-rich, and S-rich categories, respectively).

Viruses tended to exhibit some of the highest per-residue occupancy values across many LCD classes (Fig 9D), but this often occurs in exceptionally small proteomes. For example, the two extremely high values– 48.4% occupancy for F-rich LCDs and 14.3% occupancy for I-rich LCDs–occur in the Cotton leaf curl Multan betasatellite and Bhendi yellow vein mosaic betasatellite, respectively. Both betasatellites depend on helper viruses for their replication and encode a single protein, making their per-residue occupancy values unusually large outliers even among this set of maximum values. Similarly, some of the highest per-residue occupancy values among other domains (especially bacteria), correspond to extremely small–and potentially incomplete–proteomes. Furthermore, although the top-ranking eukaryotic proteome for F-rich LCDs is reasonably large (>50k proteins), corresponding to the pharaoh cuttlefish, *Sepia pharaonis*, its per-residue occupancy of F-rich LCDs is so high (18.5% of the proteome) that we consider it unlikely to be a legitimate proteome and we excluded it from Fig 9 and all subsequent analyses. Therefore, while the study of organisms with the highest LCD content is informative and interesting, we emphasize caution in interpreting values for small proteomes and extremely unusual outliers.

**Fig 10:**
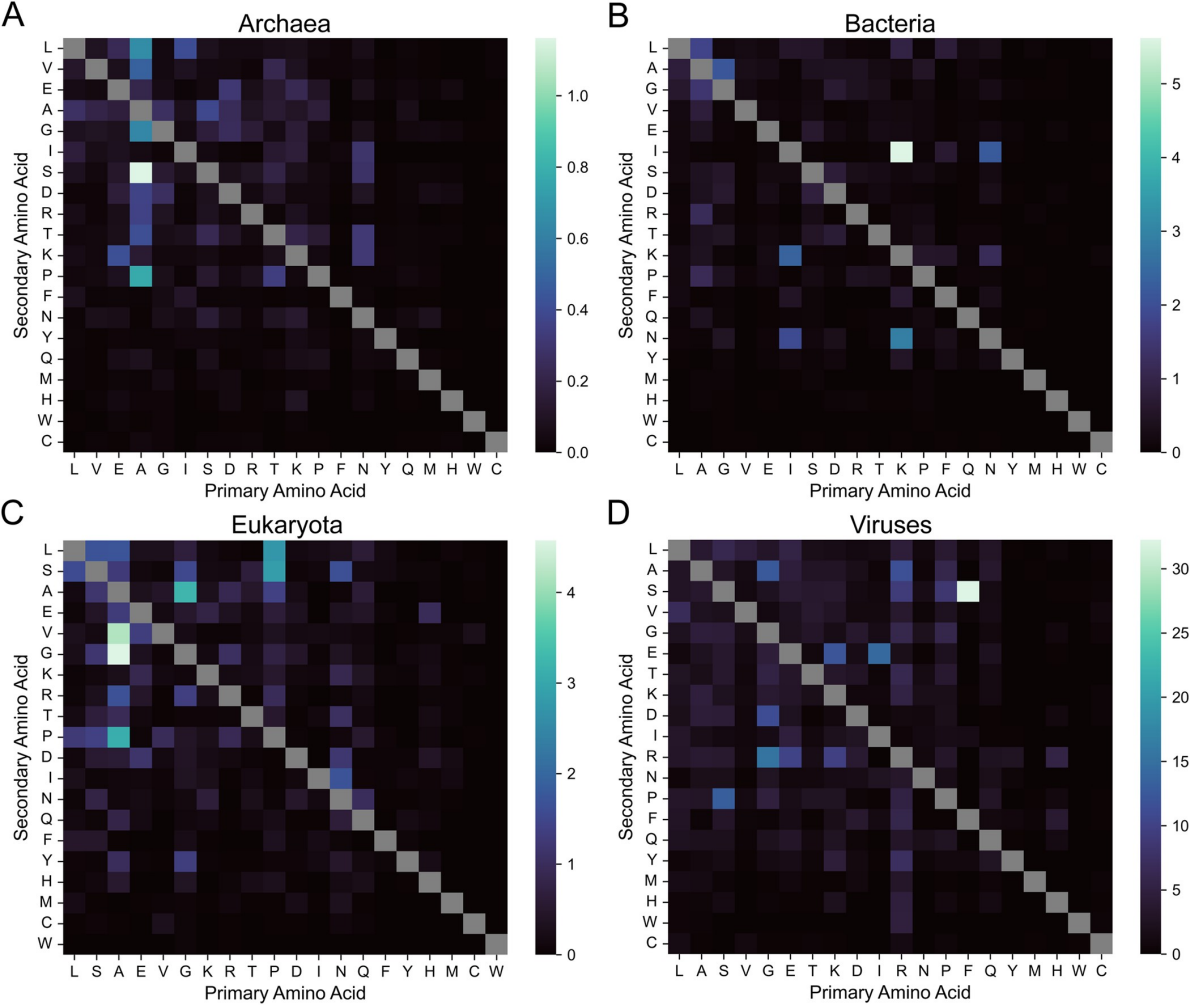
Maximum per-residue occupancy for each LCD class by domain of life. Per-residue occupancy was calculated for each LCD class and each organism. Maximum per-residue occupancy is depicted separately for each LCD class in archaea (A), bacteria (B), eukaryotes (C), and viruses (D).

We next calculated the per-residue occupancy of each secondary LCD class for each organism and focused on those with the highest values for each LCD class. Differences between specific groups of LCD classes become more prominent when per-residue occupancy is considered (Fig 10 and S11 Table). For instance, only select NX classes (i.e., those with N as the primary residue)–including the NI, NS, ND, NT, and NK classes–have relatively high maximum per-residue occupancies in eukaryotes (Fig 10C). High per-residue occupancies for NX classes are limited to the NI, NS, NT, and NK classes in archaea (Fig 10A), and high values in bacteria are further limited to the NI and NK classes only (Fig 10B). As another example, high per-residue occupancies are achieved in eukaryotes for PL, PS, PA, PG, and PR classes (all being types of PX classes; Fig 10C), whereas PX classes do not reach high per-residue occupancies in archaea or bacteria, and the highest PX values are observed for different PX classes (Fig 10A and 10B).

**Fig 11:**
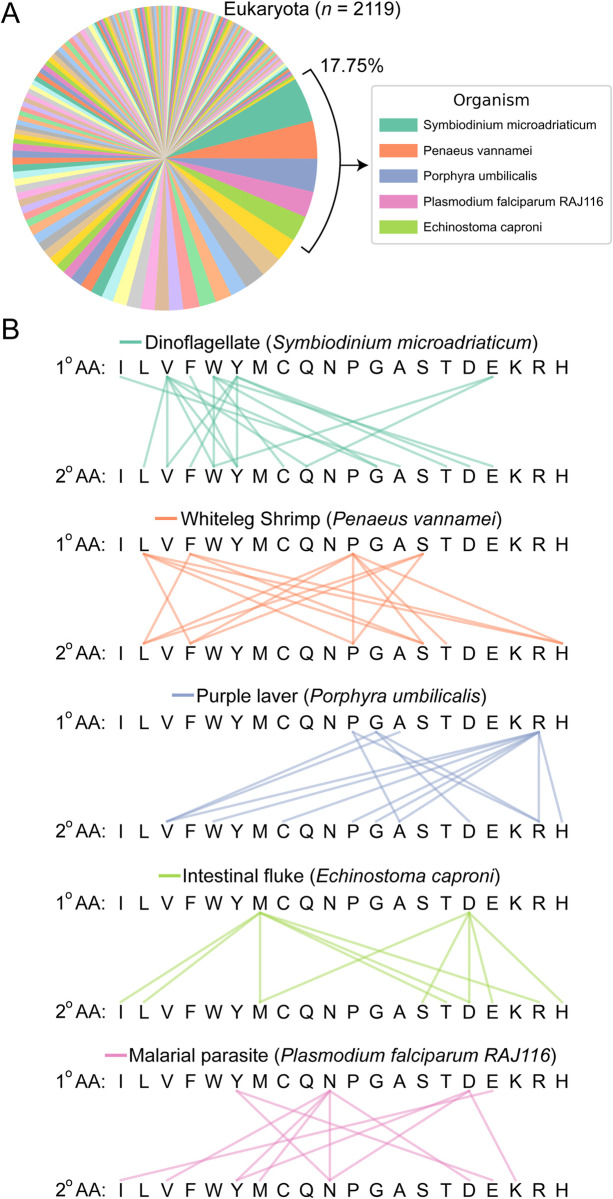
Number of LCD classes assigned to each eukaryotic organism contributing a maximum per-residue occupancy for at least one LCD class. (A) Pie chart indicating the assignment of LCD classes (400 total) to the eukaryotic organism achieving the highest per-residue LCD occupancy. Each wedge represents a single organism associated with the overall highest per-residue occupancy observed among eukaryotes. Wedge size indicates the number of LCD classes for which the single organism corresponding to that wedge achieved the highest per-residue occupancy. The top five eukaryotic organisms are indicated in the legend. Out of necessity, the color palette was repeated in the pie chart, though each color cycle represents a different set of organisms. (B) Linkage maps indicate the types of LCD classes for which the organism contributed the maximum per-residue occupancy value for eukaryotes. The first row of amino acids in each linkage map indicates the primary amino acid comprising the LCD class, and lines connected to the second row of amino acids indicate the secondary amino acid comprising the LCD class. Lines connecting identical amino acids (e.g., W connected to W) indicate that the organism contributed the maximum per-residue occupancy value for the primary LCD class as a whole (e.g., the W-rich primary LCD class). LCD classes without connecting lines are those for which the organism did not contribute the maximum per-residue occupancy value. Similar analysis for archaea, bacteria, and viruses can be found in S21 Fig. Although it achieved high per-residue occupancies for multiple LCD classes, the *Spodoptera litura* proteome was manually identified as an exceptionally incomplete reference proteome and excluded from analyses.

Certain organisms have the absolute highest per residue occupancy for multiple LCD classes, suggesting that these proteomes are extremely unusual. For example, in eukaryotes, ~18% of the 400 LCD classes are attributed to only five organisms (Fig 11A). A closer examination of these organisms reveals organism-specific clusters of LCD classes with maximum per-residue occupancy values (Fig 11B). For example, *S*. *microadriaticum*–a dinoflagellate important in coral reef ecosystems [58,59]–contributed the maximum per-residue occupancy for 19 separate LCD classes (the most from any single organism), and these classes tended to correspond predominantly to LCDs with hydrophobic or aromatic amino acids as the primary residue (Fig 11B). The whiteleg shrimp, *Penaeus vannamei*, had the highest per-residue occupancy for multiple LCD classes with L, F, P, or S as the primary amino acids (Fig 11B). A species of seaweed (purple laver; *Porphyra umbilicalis*) exhibits per-residue occupancies that are specifically high for LCD classes with R as the primary or secondary amino acid, along with a small number of P-rich or G-rich LCDs (Fig 11B). The intestinal fluke, *Echinostoma caproni*, achieves the highest per-residue occupancy for multiple LCD classes with M or D as the primary amino acid (Fig 11B). Finally, a species of malaria (*Plasmodium falciparum RAJ116*) exhibits the expected highest per residue LCD occupancy for multiple classes with N as the primary residue, along with a few additional classes containing D or Y as the primary residue (Fig 11B). Similar LCD-class specificity is also observed in the top-ranking archaeal, bacterial, and even viral organisms (S21 Fig).

Collectively, these observations highlight the extreme percentage of proteome space devoted to each class of LCD in specific organisms, uncover organism-specific preferences for certain LCDs, and identify organisms with exceptionally unusual proteomes.

### LCD-Centric Comparison of Whole Proteomes via Analysis of LCD Class Distributions and Per-Residue LCD Occupancy

We have shown previously that primary LCD frequencies vary across organisms, resulting in unique LCD frequency profiles [9]. By extension, we expected secondary LCD frequencies to also vary across organisms. In order to perform a direct cross-organism comparison of the distributions of secondary LCDs within each primary LCD class, all instances of secondary LCDs were collected for each organism. For each secondary LCD class, the “share” of secondary LCDs was calculated as the number of LCDs in that class divided by the total number of secondary LCDs with the same primary amino acid, expressed as a percentage (e.g., the share of HQ LCDs is the percentage of HQ LCDs out of all HX LCDs).

**Fig 12:**
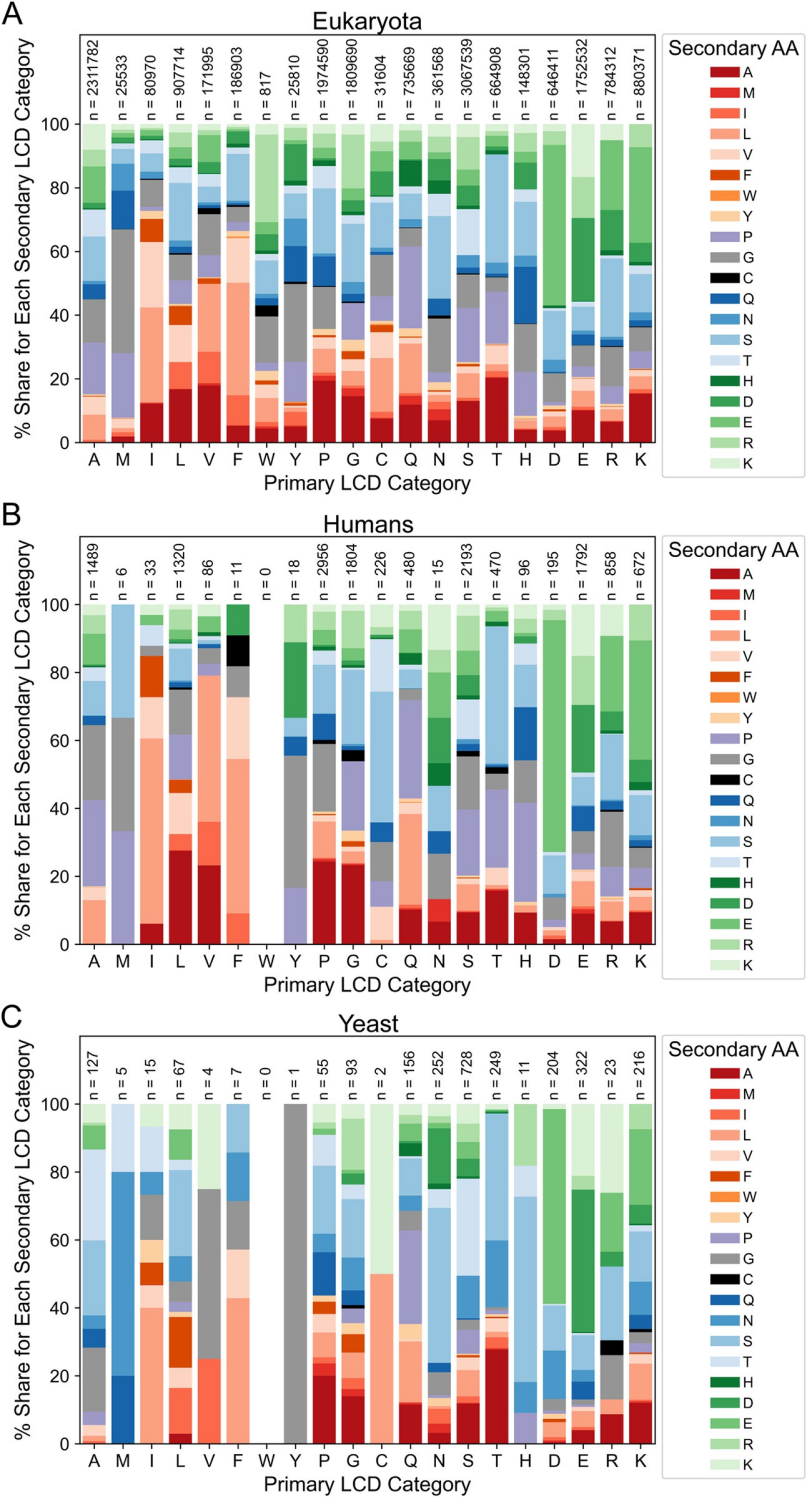
Comparison of secondary LCD distributions among eukaryotes. (A) Distributions of secondary LCDs within primary LCD categories for all secondary LCDs identified in eukaryotic organisms. (B) Distributions of human secondary LCDs among primary LCD categories. (C) Distributions of yeast secondary LCDs among primary LCD categories. For all panels, secondary LCDs were grouped into a primary LCD category based on the predominant amino acid used in the LCD search. Then, within each primary LCD category, the percentage of total LCDs in that category was calculated for all possible secondary LCD categories and depicted as a stacked bar plot. For all secondary amino acid classes, the primary amino acid is represented on the *x*-axis, the bar color specifies the secondary amino acid, and the size of the bar indicates the percentage. Secondary amino acids are loosely grouped and colored according to physicochemical properties and appear in the same order (from bottom to top) as in the figure legends. Total LCD sample sizes are indicated above each bar.

Differences are apparent both when comparing two individual organisms and when comparing a single organism to all organisms in the same domain of life (Fig 12). For example, human A-rich secondary LCDs exhibit expanded shares devoted to AP and AG classes compared to eukaryotes in general (Fig 12A and 12B), with a corresponding contraction of other A-rich classes in humans relative to eukaryotes. Additional differences are observed among other classes: for example, VL, HP, and DE classes are all expanded within their respective primary LCD categories in humans relative to eukaryotes, necessitating compensatory contraction of other secondary classes in the same primary class (Fig 12A and 12B). Similar types of comparisons can be made between the LCD profiles for individual organisms. For example, in humans, P very frequently serves as the secondary amino acid across a variety of primary LCD classes, whereas P is substantially less common as the secondary amino acid in yeast (Fig 12B and 12C). Conversely, N frequently serves as the secondary amino acid in yeast but not in humans. While other differences are also readily apparent, these representative examples highlight the variation in LCD content profiles across organisms.

**Fig 13:**
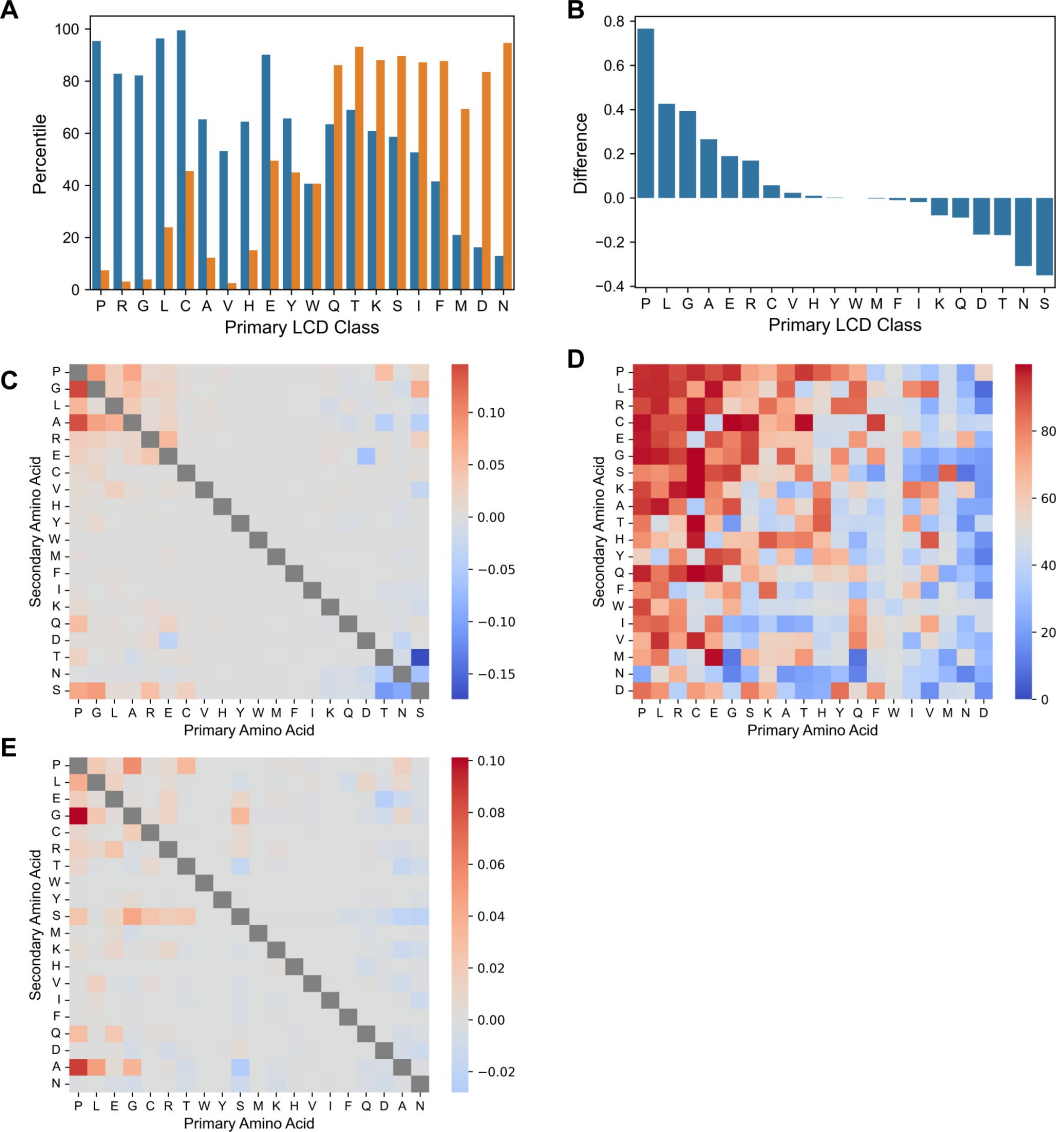
Comparison of organisms based on whole-proteome per-residue occupancy of LCDs. (A) Percentile rank for each of the primary LCD classes in humans (blue) and yeast (orange) relative to all eukaryotes. The primary LCD classes (*x*-axis) are sorted by difference in percentile from largest to smallest. (B) Raw difference in per-residue occupancy for each of the primary LCD classes in humans compared to yeast. (C) Raw difference in per-residue occupancy between humans and yeast for each of the secondary LCD classes. (D) Percentile rank for each of the secondary LCD classes in humans relative to all eukaryotes. (E) Raw difference in per-residue occupancy between humans and the corresponding average value among eukaryotes for each of the secondary LCD classes.

Quantification of LCD content as per-residue occupancy also facilitates equitable cross-proteome comparisons since it normalizes for proteome size and accounts for differences in LCD lengths. LCD content for each proteome can be represented by a 400-attribute array of per-residue occupancy values corresponding to the 20 primary LCD classes and 380 secondary LCD classes. Data in this work as well as prior work [9,11,12,60–65] suggests that organisms exhibit characteristic “signatures” of LCD content. With this classification scheme, LCD-content signatures could be used to compare organisms. As a representative example, the LCD-content signature–expressed as the percentile rank for every LCD class compared to all eukaryotes–of the human proteome was compared to the LCD-content signature of *Saccharomyces cerevisiae* (budding yeast). Humans exhibit high percentile ranks for the P, R, G, L, C, and E primary LCD classes, indicating that these classes occupy a larger percentage of the human proteome compared to most eukaryotes (Fig 13A). In striking contrast, yeast exhibits high percentile ranks for the Q, T, K, S, I, F, M, D, and N primary LCD classes. Other LCD classes exhibit little or no difference in per-residue occupancy, likely because many of them are not common in both yeast and humans.

Raw per-residue occupancy values can also be directly compared, illuminating the actual magnitude of differences in LCD content between organisms. For example, by percentile rank, yeast and humans differ substantially with respect to N-rich LCD content but only modestly with respect to S-rich LCD content (Fig 13B). However, by raw difference in per-residue occupancy, the difference between humans and yeast is more extreme with respect to S-rich LCDs than N-rich LCDs (Fig 13A), likely reflecting, at least in part, the abundance of S-rich LCDs in yeast cell wall proteins [60]. Indeed, among the secondary LCD classes, the most negative raw difference in per-residue occupancy (indicating higher LCD content in yeast relative to humans) occurs specifically for the ST and TS classes common among yeast cell wall proteins, whereas humans actually exhibit a higher per-residue occupancy for the SG, SR, and SP classes (Fig 13C).

In addition to pairwise comparisons between organisms, individual organisms can be compared to the average per-residue occupancy among all other organisms of the corresponding domain of life. Again, both the percentile rank and the raw difference in per-residue occupancy relative to the mean offer complementary comparisons. Humans achieve a very high percentile rank for nearly all PX secondary LCD classes (Fig 13D), indicating that these classes have unusually high per-residue occupancy in humans relative to other eukaryotes. However, PG LCDs (and, to a lesser extent, PA LCDs) clearly have the highest raw difference in per-residue occupancy relative to the mean (Fig 13E), highlighting the LCD classes for which humans differ from other eukaryotes by the greatest magnitude. Consistent with the notion that organisms exhibit LCD content signatures, extension of the pairwise comparisons of LCD content profiles to all organisms examined in this study enables a rudimentary clustering of clades from the same domain of life based solely on LCD content (as indicated by the clustering of like colors representing the domains of life in S22 Fig).

In summary, organisms across the domains of life exhibit unique signatures of LCD content, often correlating with functional specificity associated with the LCD-containing proteins. Resolving LCDs into a hierarchy of LCD classes based on prominent compositional features of the LCDs aids in the detection of differences in LCD content as well as class-specific functions associated with LCDs.

## Discussion

Our study was founded upon four related guiding questions: how much does LCD content vary across organisms, where is each type of LCD found, how common is each type of LCD, and what are the functions associated with LCDs? We find that even the most unusual categories of LCDs are present in nature, sometimes at extremely high levels in particular organisms. More broadly, organisms can differ dramatically with respect to their overall LCD content profiles. In some organisms, biased genome compositions contribute substantially to LCD frequencies. However, we find that LCDs are often significantly enriched despite these biases. Furthermore, organisms must have ways to leverage (or at least tolerate) such unusual features of their proteomes regardless of their origins.

By definition, our inquiry into rare and unusual LCDs is, to some degree, a foray into understudied corners of biology. One of the limitations of this study is the paucity of information and the difficulty in experimentally characterizing non-model organisms. As a result, we have relied heavily on computational tools, predictions, and the limited information currently available. Although the existing proteome resources are extremely well-maintained, some of the proteomes in our dataset await detailed experimental validation, including direct detection of all proteins *in vivo*. Furthermore, some instances of LCDs may be attributed to errors in gene prediction and annotation [11]. It is possible that the same errors even persist to the present day, nearly 20 years later: CV LCDs were previously implicated as one of the LCD classes with potential gene prediction errors, and we noticed a large number of CV LCDs (particularly CV dipeptide repeats) in certain aquatic organisms (S1 Table), including the two pufferfish species originally examined [11]. Therefore, while the proteomes may indicate the presence of LCDs, their existence and abundance in the corresponding organism may not be fully known. Additionally, the extant set of reference proteomes is a skewed representation of taxonomic groups, presumably due both to real-world differences in the prevalence of species from each taxonomic group, as well as research biases favoring the study of specific taxonomic groups [34,35]. As collective proteomic data is updated to more accurately reflect the global distribution of taxonomic groups and species, our corresponding comparisons of LCD frequencies may need to be updated as well. Our intention is for this study to serve as a launch-point for further investigation.

LCDs of some types were found at reasonably high levels in well-studied organisms, enabling their examination at greater depths. For example, HQ LCDs were present in multiple organisms (including two model organisms, *D*. *melanogaster* and *D*. *discoideum*, with high HQ LCD content) and were associated with strongly overlapping sets of transcription and RNA-related functions. Q/H-rich LCDs can serve as pH sensors in multiple organisms through alterations in protein structure and activity [22,23], even when H levels are not particularly abundant within the LCD [66]. It is tempting to speculate that HQ LCDs may be widely used pH-responsive LCDs commonly associated with transcription and gene expression, as observed recently for the yeast transcription factor, Snf5 [66], and the mRNA-binding and translation-regulating Orb2 protein in *Drosophila* [22,23].

Keratin proteins emerged as an exemplar family of eukaryote-specific proteins with distinct subcategories of LCDs. Multiple CX LCD classes were represented in a relatively large number of proteins in a variety of well-studied eukaryotic organisms. Given their purpose as structural proteins in hair, skin, nails, claws, and other related features, these LCDs were sensibly identified as eukaryote-specific LCDs. However, this also suggests that CX LCDs are not frequently utilized for other functions in non-eukaryotic organisms. Additionally, LCD-Composer provided the specificity to distinguish between proteins of the same keratin family with distinct classes of LCDs and LCD profiles, while also detecting overlap between LCD classes among keratins. The high number of proteins with these LCDs in well-studied organisms, along with the specialization of certain categories of LCDs among the keratin family, made keratins a useful model for eukaryote-specific LCDs and even revealed organism-specific utilization of distinct balances of LCDs among keratins in each organism. However, not all C-rich LCDs were associated with keratin-related functions: multiple classes of C-rich LCDs were associated with different functions in different organisms (notably, CK LCDs with functions in metallothioneins).

Functional inferences for LCD classes were made primarily based on GO-term analyses. While this method is widely implemented and is clearly useful (as exemplified by the C-rich classes of LCDs), it is not without limitations. First, enriched GO terms are simply statistical associations between groups of proteins and particular functional annotations. Not all proteins of a group will have the specified function, so caution should be exercised when extrapolating these associations to the same LCD classes in different organisms. Second, functional LCDs may have different levels of compositional enrichment required for biological activity depending on the LCD class: tailoring the composition thresholds for specific classes of LCDs may improve the sensitivity/specificity trade-off and optimize the detection of LCD-associated functions. Third, LCD function can differ between organisms and contexts. As an interesting example, a mammalian LCD normally involved in alternative splicing, protein-protein interaction, and maintaining protein solubility [67] was bioengineered to act as extremely strong and environmentally resilient extracellular adhesive protein in mussels [68]: a function that is only tangentially related to its native functions. Fourth, biases inherent in the gene ontologies may limit the functional associations that can be observed or that reach statistical significance. Finally, it is possible that there are length-dependent relationships between LCD occurrence, the corresponding sets of LCD-containing proteins, and their associated functions. LCDs may be more likely to occur in longer proteins, which may, in-turn, be related to specific functions [9,32]. However, LCDs may also occur in these proteins for functional reasons, making it difficult to disentangle length-to-function relationships. We observe clear specificity in the functions associated with distinct classes of LCDs, suggesting that length-independent relationships between LCDs and associated protein functions are still easily identifiable.

The choice of parameter thresholds will invariably affect LCD frequencies, regardless of LCD identification method. As thresholds become more stringent (e.g., increasing window size and/or composition thresholds), fewer protein regions will tend to be classified as LCDs. While the choice of window size and composition thresholds are somewhat arbitrary, our supplementary analyses suggest that they have little effect on comparisons of LCD frequencies between domains of life (S2–S4 Figs). We have previously demonstrated the utility of these thresholds in identifying functions associated specifically with secondary LCD classes [9]. While it was not computationally feasible for us to comprehensively explore all parameter combinations for all organisms and LCD classes, we encourage further exploration with alternative search parameters.

Additionally, multiple methods of LCD identification have been previously developed [1,9,69–77]. LCD-Composer was utilized in this study because it was specifically designed for this purpose: to identify and classify LCDs based solely on the compositional features of the protein sequences themselves, regardless of background amino acid frequencies, sequence repetitiveness, or sequence entropy/information content (though composition is still related to these features). Some of the cited methods would not be particularly amenable to the LCD classification approach used in this study. For example, some methods that consider sequence entropy/information content often do not relate complexity to actual biochemical features of the sequence (thereby precluding an LCD classification scheme without substantial downstream processing), while methods depending on background amino acid frequencies can result in LCD classes with radically different levels of compositional enrichment for the “defining” amino acid(s). Nevertheless, given that each LCD identification method adopts a slightly different approach, large-scale study of LCDs using alternative methods could lead to unique insights, and their use is encouraged as well.

In summary, our comprehensive survey of LCDs in the reference proteomes of all known organisms provides a powerful resource and classification system for LCDs. These LCDs may serve context-specific, adaptive functions in their native organisms, which may be potentiated by environmental conditions and/or concomitant proteome adaptations. Some organisms exhibit extremely unusual proteomes with hundreds of proteins containing rare types of LCDs, or with the highest LCD content for multiple classes of LCDs. In essence, a realized (i.e., expressed) proteome represents a major subset of the intracellular and extracellular ecosystem of biomolecules in a given organism. Considering the unusual nature of some LCD content profiles, these observations stretch our understanding of biomolecular ecosystems compatible with life.

## Methods

### Data acquisition and processing

UniProt reference proteomes for all available organisms were downloaded from the UniProt FTP site (https://ftp.uniprot.org/pub/databases/uniprot/) on 8/22/22. The gene ontology was downloaded from the Gene Ontology Consortium website (http://geneontology.org/docs/download-ontology/). Pfam annotations (version 35.0) were acquired from the Pfam FTP site (ftp://ftp.ebi.ac.uk/pub/databases/GO/goa/). Gene annotation files for GO-term analyses were downloaded from the European Bioinformatics Institute website (http://ftp.ebi.ac.uk/pub/databases/GO/goa/). GO-term analyses were performed using GOATOOLS [78] with default parameters using the same proteomes analyzed in the initial LCD searches. Subsequent analyses of enriched GO terms focused on GO terms with a minimum depth of 4 in the gene ontology to reduce the number of relatively non-specific functional associations. For in-depth analyses of C-rich LCDs, the following 13 model organisms were arbitrarily selected: *Arabidopsis thaliana* (thale cress plant), *Bos taurus* (cow), *Caenorhabditis elegans* (roundworm), *Canis lupus familiaris* (dog), *Danio rerio* (zebrafish), *Dictyostelium discoideum* (slime mold), *Drosophila melanogaster* (fruit fly), *Gallus gallus* (chicken), *Homo sapiens* (human), *Mus musculus* (mouse), *Saccharomyces cerevisiae* (yeast), *Sus scrofa* (pig), *Rattus norvegicus* (rat).

### LCD Identification and Classification

Primary LCDs were identified using LCD-Composer with default parameters [9] for each reference proteome. Briefly, a 20-amino acid sliding window was used to scan each protein sequence. Windows passing the default minimum 40% composition corresponding to a single amino acid and a minimum linear dispersion of 0.5 were classified as LCDs. All overlapping windows passing these criteria were merged to generate contiguous LCDs. LCD searches were performed separately for each of the 20 canonical amino acids.

Secondary LCDs were identified using a separate LCD-Composer search for all possible combinations of two amino acids except those where the first and second amino acid are the same (380 total combinations). For each search, the minimum composition criteria were ≥40% and ≥20% for the first and second amino acid, respectively, comprising the secondary LCD class. For example, HQ LCDs were defined as those with a minimum of 40% H and a minimum of 20% Q. It should be noted that these criteria represent minimum thresholds only: in rare cases, the percent composition of the secondary amino acid can exceed that of the primary amino acid. Searches were performed with a 20-amino acid window size and a minimum linear dispersion of 0.5 applied separately for each of the amino acids in the search. Secondary LCD searches required use of the Alpine high-performance computing resource at the University of Colorado Boulder [79].

Datasets of all primary and secondary LCDs identified in each domain of life as part of this study are available in a publicly accessible repository [33].

### Pfam domain mapping and quantification

Pfam domain annotations were mapped to LCD-containing proteins for each class across all organisms. Pfam domains were then assigned to “clans” to represent the broadest possible categories linking the more specific domain annotations. For each organism, the maximum number of LCD-containing proteins associated with a single Pfam clan was calculated for each LCD class, only considering LCD classes for which at least one protein was assigned a Pfam annotation. These values were then averaged across organisms within each domain of life.

### Proteome Scrambling and LCD Frequency Estimation

Each proteome in the dataset was scrambled once using the Fisher-Yates shuffle method. To best represent constraints of true proteomes, the starting residue of each protein (typically a methionine) was extracted prior to scrambling. The scrambled proteome was then segmented into proteins of sizes equal to those found in the corresponding non-scrambled proteome, and the initiator residue was added back to the start of each scrambled protein. LCD-Composer was run on each scrambled proteome to identify both primary LCDs and secondary LCDs for every LCD class, exactly as performed for the original proteomes. The number of proteins containing an LCD was tallied for each scrambled proteome and LCD class, then compared to the corresponding value from the original proteomes using Fisher’s exact test. Within each organism, *p*-values were adjusted using the Holm–Šidák correction method to account for multiple-hypothesis testing. The natural logarithm of the odds ratio (lnOR) and its corresponding 95% confidence interval were also calculated. When evaluating individual organisms, *p*-values corresponding to LCD classes with 0 instances in both the original and scrambled proteomes for a given organism were not calculable and were therefore excluded from multiple-test correction. However, when calculating the percentage of organisms with significant enrichment of LCDs from each LCD class, these classes were retained and considered not statistically significant (Fig 6). The median lnOR was used to estimate the typical degree of enrichment for each LCD class across organisms within each domain of life (S19 Fig). For LCD classes in which the number of proteins containing an LCD in either the original or scrambled proteomes was 0, a value of 1 was added to each cell in the contingency table to provide a biased lnOR estimate. Importantly, when a biased estimate was required, it was almost universally due to 0 LCD occurrences in the scrambled proteome: therefore, in the vast majority of cases, this procedure results in conservatively biased lnORs that underestimate the degree of enrichment observed in the original proteomes.

## Supporting information

S1 Fig LCD frequency classification across the domains of life. Each type of LCD was classified as absent (*x* = 0%), very rare (0%<*x*<5%), rare (5%≤*x*<20%), normal (20%≤*x*<50%), common (50%≤*x*<75%) or very common (*x*≥75%) separately for each domain of life, where *x* represents the percentage of organisms containing at least one instance of the LCD class. Squares on the diagonal represent the primary LCD classes, whereas off-diagonal squares represent secondary LCD classes. For each domain of life, amino acids on the axes are ordered from most-common to least-common based on the mean rank of whole-proteome frequency for each amino acid across all proteomes for that domain.
(TIF)

S2 Fig Organism-level LCD frequencies as a function of window size. 50 organisms were randomly selected from each domain of life. For each proteome, LCD-Composer searches were repeated using window sizes from 20 to 60 amino acids (in steps of 10) for all LCD classes with the original composition thresholds (≥40% for the primary amino acid and ≥20% for the secondary amino acid). (A) Heatmap depicting the percentage of organisms containing at least 1 LCD for each combination of domain of life, window size, and LCD class. Within each grid section representing a single LCD class, the *y*-axis represents the domain of life in the order Viruses➔Archaea➔Bacteria➔Eukaryota (from top to bottom), while the *x*-axis represents the window size in the order 20➔30➔40➔50➔60 (from left to right), as shown in the key below panel A. (B) Categorical heatmap depicting the domain of life with the highest percentage of organisms containing at least 1 LCD for each combination of window size and LCD class. The *x*-axis represents the window size in the order 20➔30➔40➔50➔60 (from left to right), as shown in the key above panel B. In addition to the categories corresponding to a domain of life, the “Tied” category indicates parameter combinations for which 2 or more domains of life had an identical percentage of organisms with 1 or more LCD, whereas the “Zero” category indicates parameter combinations for which none of the sampled organisms in all 4 domains of life contain an LCD in the indicated LCD class. LCD content increases for most LCD classes when progressing from Viruses➔Archaea➔Bacteria➔Eukaryota. For both figure panels, grid sections on the diagonal represent the primary LCD classes, whereas all other grid sections represent secondary LCD classes.
(TIF)

S3 Fig Organism-level LCD frequencies as a function of composition thresholds. For each of the 50 randomly selected proteomes from each domain of life (those evaluated in S2 Fig), LCD-Composer searches were repeated with the primary composition threshold ranging from 30 to 60 (in steps of 10) and the secondary composition threshold ranging from 20 to 60 (in steps of 10). In these searches, window size was held constant at 20 amino acids. For each domain of life, the heatmap depicts the percentage of organisms containing at least 1 LCD for each combination of primary composition threshold, secondary composition threshold, and LCD class. Within each grid section representing a single LCD class, the *y*-axis represents the secondary composition threshold in the order 20➔30➔40➔50➔60 (from top to bottom), while the *x*-axis represents the primary composition threshold in the order 30➔40➔50➔60 (from left to right), as shown in the key above the figure. Grey squares in the lower-right corner of each grid square represent invalid composition thresholds where the sum of the primary and secondary thresholds exceeds 100%. As with varying window size, LCD content increases for most LCD classes when progressing from Viruses➔Archaea➔Bacteria➔Eukaryota regardless of the primary and secondary composition thresholds. For each heatmap, grid sections on the diagonal represent the primary LCD classes, whereas all other grid sections represent secondary LCD classes. For primary LCD classes, the composition threshold on the *y*-axis is ignored since only one composition threshold is relevant. Note that, for any given grid section, the composition thresholds (rather than the axis labels) determine which amino acid is considered the “primary” amino acid (larger composition threshold) and which is considered the “secondary” amino acid (smaller composition threshold) comprising the LCD class.
(TIF)

S4 Fig Categorical representation of the domains with the highest percentage of organisms with ≥1 LCD for each combination of primary and secondary composition thresholds. The heatmap is arranged identically to those in S3 Fig but depicts the domain of life with the highest percentage of organisms containing ≥1 LCD for each combination of primary composition threshold, secondary composition threshold, and LCD class. Grid sections on the diagonal represent the primary LCD classes, whereas all other grid sections represent secondary LCD classes. For primary LCD classes, the composition threshold on the *y*-axis is ignored since only one composition threshold is relevant. Note that, for any given grid section, the composition thresholds (rather than the axis labels) determine which amino acid is considered the “primary” amino acid (larger composition threshold) and which is considered the “secondary” amino acid (smaller composition threshold) comprising the LCD class. In addition to the categories corresponding to a domain of life, the “Tied” category indicates parameter combinations for which 2 or more domains of life had an identical percentage of organisms with 1 or more LCD, the “Zero” category indicates parameter combinations for which none of the sampled organisms in all 4 domains of life contain an LCD in the indicated LCD class, and the “Invalid threshold” category indicates composition threshold combinations for which the sum of the primary and secondary thresholds would exceed 100%. In the majority of “Tied” cases, the percentage of organisms containing an LCD was 100% for 2 or more organisms. For nearly all LCD classes, eukaryotes exhibit the highest percentage of organisms with ≥1 LCD regardless of the primary and secondary composition thresholds.
(TIF)

S5 Fig Organism-level primary LCD frequencies by archaeal clade. For each primary LCD class, organism-level LCD frequencies were evaluated separately for each basic archaeal clade. Clades were derived from taxonomic lineages and were the first term following the domain of life. Each bar color corresponds to only one clade (specified on the *x*-axis) throughout all subplots. Numbers in parentheses indicate the number of organisms evaluated for the corresponding clade.
(TIF)

S6 Fig Organism-level primary LCD frequencies by bacterial clade. For each primary LCD class, organism-level LCD frequencies were evaluated separately for each basic bacterial clade. Figure details are as described for S5 Fig, except that only the top 10 bacterial clades (with respect to the number of organisms evaluated) are included for simplicity.
(TIF)

S7 Fig Organism-level primary LCD frequencies by eukaryotic clade. For each primary LCD class, organism-level LCD frequencies were evaluated separately for each basic eukaryotic clade. Figure details are as described for S5 Fig.
(TIF)

S8 Fig Organism-level primary LCD frequencies by viral clade. For each primary LCD class, organism-level LCD frequencies were evaluated separately for each basic viral clade. Figure details are as described for S5 Fig, except that only the top 10 viral clades (with respect to the number of organisms evaluated) are included for simplicity.
(TIF)

S9 Fig Functions associated with LCD classes in viruses. Top 25 GO term and LCD class pairs with respect to the percentage of viruses sharing significant enrichment for that pair. Only GO terms that were significantly enriched (Šidák-corrected *p* < 0.05) and had a minimum depth of 4 in the gene ontology are shown. Bar coloring and text coloring conventions are as described in the Fig 2 legend.
(TIF)

S10 Fig Top-ranking functions of C-rich LCDs in eukaryotes. (A) Top-ranking functions associated with C-rich LCDs as indicated in Fig 3A but with inclusion of XC LCD classes. GO terms are sorted by the percentage of eukaryotic organisms with significant enrichment of the LCD class/GO term pair and are limited to the top 50 GO terms. (B) Top-ranking functions associated with CX LCDs in mammals only, limited to the top 50 GO terms. In both panels, bar color corresponds to LCD class, with reciprocal classes (e.g., CK and KC LCDs) assigned the same color for simplicity. GO terms on the *x*-axis are colored according to the GO-term category with Biological Process (BP) in red, Cellular Component (CC) in green, and Molecular Function (MF) in blue. Only GO terms that were significantly enriched (Šidák-corrected *p* < 0.05) and had a minimum depth of 4 in the gene ontology are shown.
(TIF)

S11 Fig LCD domain layout within human keratin and keratin-associated proteins. Schematic depicting LCD types and their locations within human keratin/keratin-associated proteins. LCD types are indicated by their two-letter abbreviations, and labels are staggered in cases where the LCDs overlap.
(TIF)

S12 Fig LCD domain layout within mouse keratin and keratin-associated proteins. Same as S11 Fig, but for mouse keratin/keratin-associated proteins.
(TIF)

S13 Fig LCD domain layout within rat, cow, pig, and dog keratin and keratin-associated proteins. Same as S11 Fig, but for rat, cow, pig, and dog keratin/keratin-associated proteins.
(TIF)

S14 Fig Analysis of Pfam clan annotations associated with each LCD class. (A) Total number of proteins (blue) and the number of proteins associated with a single Pfam clan (red) were calculated for all LCD classes in the human proteome. (B) The average of maximum percentages of proteins associated with each LCD class was calculated across organisms within each domain. A minimum of 5 proteins were required to be included in the calculation to mitigate skewing of percentages by small sample sizes. Grey boxes indicate primary LCD classes and LCD classes for which none of the organisms had ≥5 associated proteins.
(TIF)

S15 Fig Functions of H-rich LCDs in bacteria and archaea. (A) GO terms associated with H-rich LCDs (including HX and XH LCD classes) in bacteria, sorted by the percentage of bacterial organisms with enrichment of each LCD class/GO term pair and limited to the top 50 pairs. (B) GO terms associated with H-rich LCDs in archaea. All LCD class/GO term pairs with significant enrichment in at least one organism are displayed. In both panels, bar color corresponds to LCD class, with reciprocal classes (e.g., HG and GH LCDs) assigned the same color for simplicity. GO terms on the *x*-axis are colored according to the GO-term category with Biological Process (BP) in red, Cellular Component (CC) in green, and Molecular Function (MF) in blue. Only GO terms that were significantly enriched (Šidák-corrected *p* < 0.05) and had a minimum depth of 4 in the gene ontology are shown.
(TIF)

S16 Fig Functions associated with high-ranking, eukaryote-specific, H-rich LCD classes. Bar plots indicate the percentage of eukaryotic organisms exhibiting significant enrichment for each LCD class/GO term pair for HL LCDs (A), NH LCDs (B), and NM LCDs (C). LCD class/GO term pairs are sorted by the percentage of eukaryotic organisms sharing significant enrichment, limited to the top 25 LCD class/GO term pairs. In all panels, GO terms on the *x*-axis are colored according to the GO-term category with Biological Process (BP) in red, Cellular Component (CC) in green, and Molecular Function (MF) in blue. Only GO terms that were significantly enriched (Šidák-corrected *p* < 0.05) and had a minimum depth of 4 in the gene ontology are shown.
(TIF)

S17 Fig Percentage of organisms with enrichment or depletion of LCDs, regardless of statistical significance. The percentage of organisms with enrichment (positive lnORs) or depletion (negative lnORs) was calculated as described for Fig 6 but without imposing a statistical significance threshold. The panels in the left column (panels A, C, E, and G) indicate the percentage of organisms with LCD enrichment in the original proteome for each LCD class. The panels in the right column (panels B, D, F, and H) indicate the percentage of organisms with LCD depletion in the original proteome for each LCD class. For each pair of heatmaps corresponding to a single domain of life, the heatmap scales are identical to facilitate direct comparison.
(TIF)

S18 Fig Percentage of organisms with statistically significant depletion of LCDs. The percentage of organisms with statistically significant depletion (negative lnORs) was calculated as described for Fig 6. Heatmaps depict percentages for eukaryotes only, as all off-diagonal values for archaea, bacteria, and viruses were exactly 0: however, all data underlying these heatmaps, as well as data corresponding to the other domains of life, can be found in the supplementary data available at [33]. Panels in the left column depict heatmaps with scales set by the minimum and maximum values within each heatmap. Panels in the right column depict heatmaps with scales identical to those in Fig 6 to facilitate direct comparison.
(TIF)

S19 Fig Median degree of LCD enrichment for each LCD class across the domains of life. For each organism, the natural logarithm of the odds ratio (lnOR) for each LCD class was used to quantify the degree of LCD enrichment or depletion in the original proteome relative to a scrambled version of that proteome. Heatmaps depict the median lnOR for each LCD class among archaea (A), bacteria (B), and eukaryotes (C). LCD enrichment data are not shown for viruses since all median lnORs are exactly 0: however, all data underlying these heatmaps, as well as data corresponding to viruses, can be found in the supplementary data available at [33]. No negative median lnOR values were observed for any of the domains of life. The diagonals indicate the median lnOR for each primary LCD class. For LCD classes in which the number of LCDs in either the original or scrambled proteomes were 0, a value of 1 was added to all cells in the contingency table to calculate a biased lnOR (see Methods).
(TIF)

S20 Fig Domain of life with the highest per-residue LCD occupancy in a randomly selected subset of organisms, with varied LCD search parameters. (A) Domain of life with the highest per-residue LCD occupancy for each LCD class while varying the window size parameter during the LCD searches. (B) Domain of life with the highest per-residue LCD occupancy for each LCD class while varying the composition threshold parameters during the LCD searches. In both panels, per-residue LCD occupancies were calculated from the same randomly selected subset of organisms evaluated for S2–S4 Figs. Figure layouts, formatting, and color categories are as described for S2 and S4 Figs, respectively.
(TIF)

S21 Fig Number of LCD classes assigned to each archaeal, bacterial, or viral organism contributing a maximum per-residue occupancy for at least one LCD class. (A) Pie chart indicating the assignment of LCD classes (400 total) to the archaeal organism achieving the highest per-residue LCD occupancy. Each wedge represents a single organism associated with the overall highest per-residue occupancy observed among archaea. Wedge size indicates the number of LCD classes for which the single organism corresponding to that wedge achieved the highest per-residue occupancy. (B) Linkage maps indicating the types of LCD classes for which the organism contributed the maximum per-residue occupancy value for archaea. The first row of amino acids in each linkage map indicates the primary amino acid in the LCD class, and lines connected to the second row of amino acids indicate the secondary amino acid in the LCD class. Lines connecting identical amino acids indicate that the organism contributed the maximum per-residue occupancy value for the primary LCD class as a whole (e.g., the W-rich primary LCD class). LCD classes without connecting lines are those for which the organism did not contribute the maximum per-residue occupancy value. Identical analyses were performed for bacteria (C,D) and viruses (E,F). For all pie charts, the top five organisms are indicated in the legend. Out of necessity, the color palette was repeated in each pie chart, though each color cycle represents a different set of organisms.
(TIF)

S22 Fig Clustering of domains and clades based on per-residue LCD occupancies. Per-residue occupancy values for all 380 secondary classes were calculated from each proteome and compared in a pairwise fashion by calculating the Manhattan distance between per-residue occupancy arrays for all possible pairs of organisms, excluding self-comparisons which evaluate to 0. (A) Heatmap indicating the average pairwise Manhattan distance within and between domains of life. (B) Heatmap indicating the average pairwise Manhattan distance within and between basic clades (i.e., the first term following the domain of life in the taxonomic lineage). Colors to the left of the heatmap indicate the domain of life corresponding to each clade on the *y*-axis. In both panels, clustering was calculated using complete linkage and Manhattan distance. Viruses were excluded from comparisons due to their exceptionally small proteomes, leading both to sparse arrays (the complete absence of LCDs for most classes) and volatile individual values (existing LCDs occupying relatively large percentages of their small proteomes). Note that the dendrogram does not represent evolutionary relationship *per se*: it simply depicts the degree of clustering based on the distances between whole-proteome LCD content.
(TIF)

S1 Table Number of proteins with LCDs for each LCD class and organism. Column headers for columns 7–407 indicate the LCD class, with primary LCD classes indicated by a single amino acid and secondary LCD classes indicated by two amino acids. Values indicate the number of proteins in the corresponding organism containing at least one LCD of the indicated LCD class.
(ZIP)

S2 Table Organism-level LCD frequencies among archaea for all LCD classes. Column headers indicate the primary amino acid in the LCD class, whereas row headers indicate the secondary amino acid in the LCD class. The diagonal (where the primary and secondary amino acid are identical) indicate values for the primary LCD classes. Values indicate the percentage of archaeal organisms containing at least one instance of the indicated LCD class. Columns and rows are sorted by mean amino acid frequency rank across archaeal organisms.
(TSV)

S3 Table Organism-level LCD frequencies among bacteria for all LCD classes. Same as S2 Table but with values corresponding to bacterial organisms.
(TSV)

S4 Table Organism-level LCD frequencies among eukaryotes for all LCD classes. Same as S2 Table but with values corresponding to eukaryotic organisms.
(TSV)

S5 Table Organism-level LCD frequencies among viruses for all LCD classes. Same as S2 Table but with values corresponding to viruses.
(TSV)

S6 Table List of randomly selected proteomes used for testing alternative LCD search parameters. For each domain of life, 50 proteomes were selected at random. For each proteome, LCD-Composer searches were repeated with a range of window sizes (S2 and S20A Figs) and composition thresholds (S3–S4 and S20B Figs).
(TSV)

S7 Table LCD classes significantly associated with at least one functional annotation. For each LCD class, the set of proteins with at least one LCD from the given class was tested for enriched functions by GO-term analysis. GO-term analyses were performed separately for all LCD classes in each organism with an available gene ontology. For any GO term significantly enriched (Šidák-corrected *p* < 0.05) for an LCD class in at least one organism, GO-term results for all other organisms in the same domain of life were searched to determine the number and percentage of organisms for which the same LCD class exhibited significant enrichment for the same GO term. Each row represents a single LCD class/GO term pair in one domain of life, along with the GO-term information, the number and percentage of organisms in that domain of life exhibiting significant enrichment, and Šidák-corrected *p*-values from each individual organism exhibiting significant enrichment.
(ZIP)

S8 Table Keratin-associated proteins with C-rich LCDs in model organisms. For each C-rich LCD class containing at least one protein associated with the GO terms “keratin filament” or “keratinization” in the GO-term analyses, the UniProt IDs of the corresponding proteins for the indicated LCD class are listed for select model organisms.
(TSV)

S9 Table GO-term analysis summary for proteins with HQ LCDs in eukaryotes. For each organism, GO-term analysis was performed on three related sets of proteins: 1) all proteins with an HQ LCD; 2) all proteins with an HQ LCD but excluding those at least 20 residues within a spatially distinct QX, XQ, HX, or XH LCD (where X is any amino acid except H for the QX and XQ classes, and X is any amino acid except Q for the HX and XH classes); 3) all proteins with an HQ LCD but excluding those with at least 20 residues within a spatially distinct Q-rich primary LCD; and 4) all proteins with an HQ LCD but excluding those with at least 20 residues within a spatially distinct H-rich primary LCD. To reduce the number of files and file size, only statistically significant GO terms (Šidák adjusted *p* < 0.05) with a minimum depth of 4 in the gene ontology were collected from individual results files.
(TSV)

S10 Table Mean per-residue LCD occupancy for each LCD class within each domain of life. Per-residue occupancy values were calculated for each LCD class in each organism. Then, for each LCD class, per-residue occupancy values were averaged within each domain of life.
(TSV)

S11 Table Per-residue-level statistics for the 3 highest-scoring organisms across LCD classes for each domain of life. The first two columns indicate the LCD class and the domain of life, respectively. The last column indicates the average per-residue occupancy for the indicated LCD class within the indicated domain of life. The remaining columns indicate the maximum per-residue occupancy, total number of proteins in the proteome, and total number of amino acids in the proteome for the 3 organisms with the highest per-residue LCD occupancy for the given LCD class and domain of life. These values and the corresponding organism names are ordered from highest-to-lowest per-residue occupancy and are separated by semicolons within each cell.
(TSV)
