# Supplementary figures and images for: Identification of Low-Complexity Domains by Compositional Signatures Reveals Class-Specific Frequencies and Functions Across the Domains of Life
Source: PLoS Comput Biol. 2024 May 15;20(5):e1011372. doi: 10.1371/journal.pcbi.1011372 (PMC11132505; doi:10.1371/journal.pcbi.1011372)

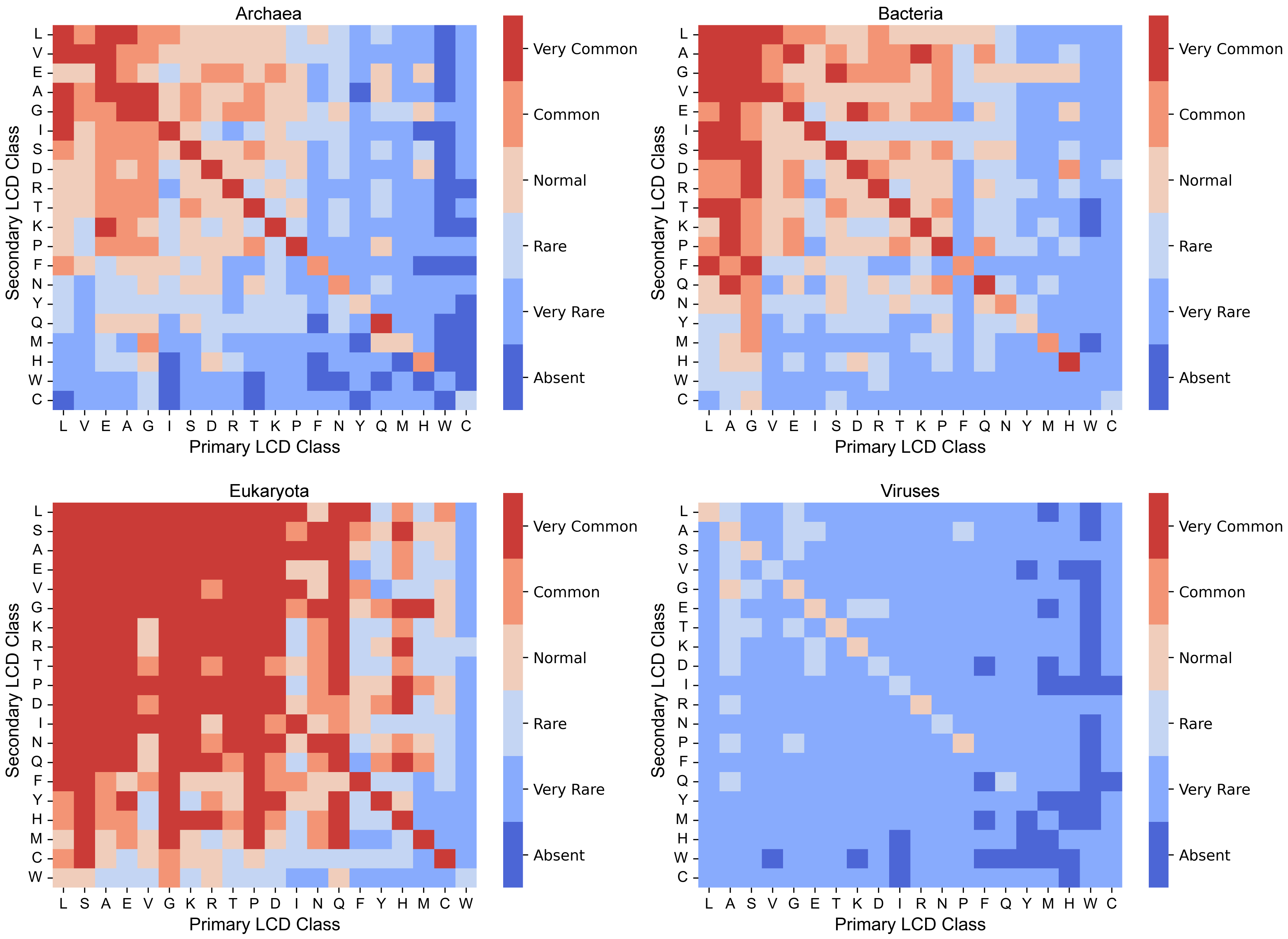

Supplement: S1 Fig — Each type of LCD was classified as absent (x = 0%), very rare (0%<x<5%), rare (5%≤x<20%), normal (20%≤x<50%), common (50%≤x<75%) or very common (x≥75%) separately for each domain of life, where x represents the percentage of organisms containing at least one instance of the LCD class. Squares on the diagonal represent the primary LCD classes, whereas off-diagonal squares represent secondary LCD classes. For each domain of life, amino acids on the axes are ordered from most-common to least-common based on the mean rank of whole-proteome frequency for each amino acid across all proteomes for that domain. (TIF) [file pcbi.1011372.s001.tif]

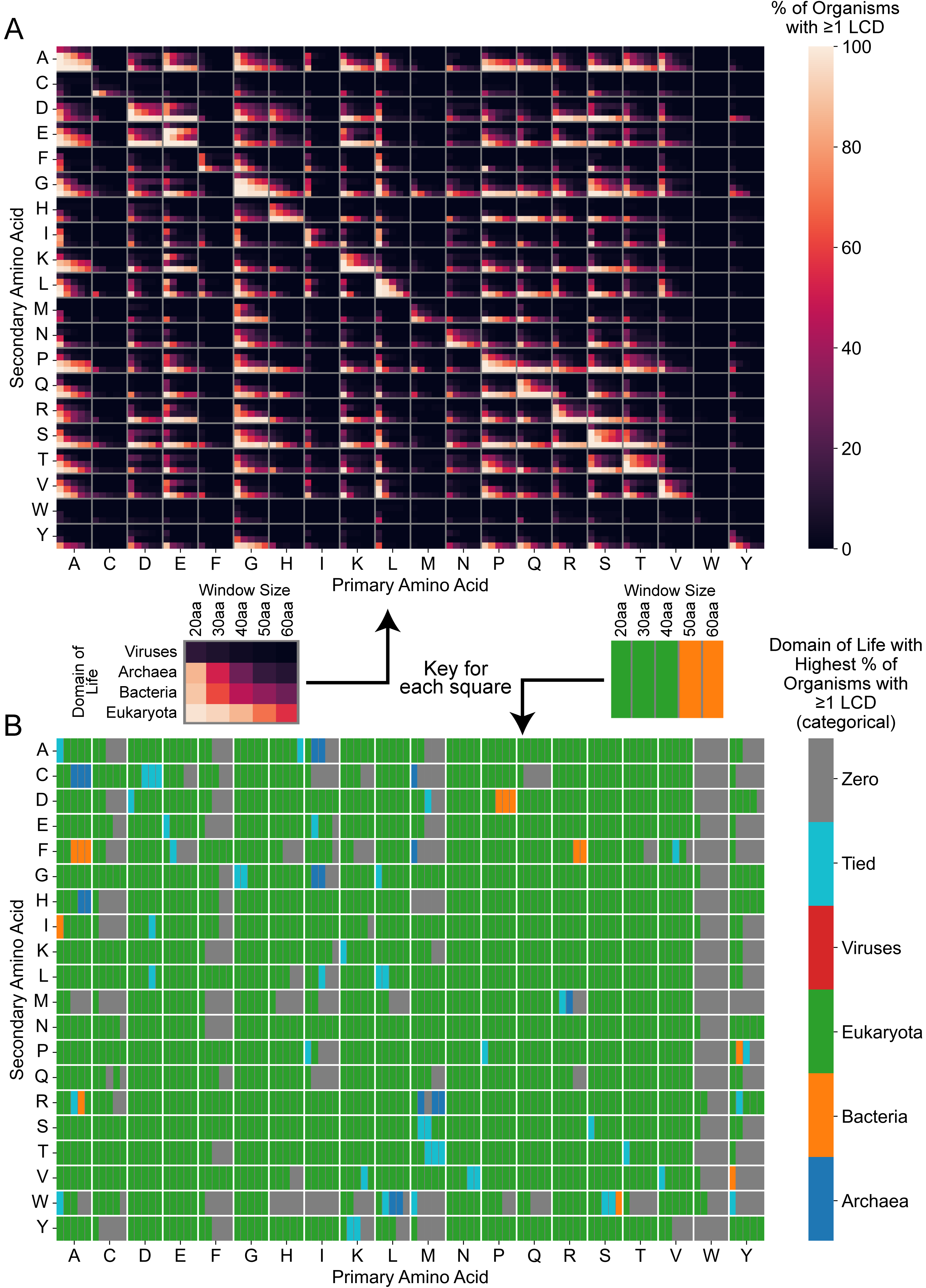

Supplement: S2 Fig — 50 organisms were randomly selected from each domain of life. For each proteome, LCD-Composer searches were repeated using window sizes from 20 to 60 amino acids (in steps of 10) for all LCD classes with the original composition thresholds (≥40% for the primary amino acid and ≥20% for the secondary amino acid). (A) Heatmap depicting the percentage of organisms containing at least 1 LCD for each combination of domain of life, window size, and LCD class. Within each grid section representing a single LCD class, the y-axis represents the domain of life in the order Viruses➔Archaea➔Bacteria➔Eukaryota (from top to bottom), while the x-axis represents the window size in the order 20➔30➔40➔50➔60 (from left to right), as shown in the key below panel A. (B) Categorical heatmap depicting the domain of life with the highest percentage of organisms containing at least 1 LCD for each combination of window size and LCD class. The x-axis represents the window size in the order 20➔30➔40➔50➔60 (from left to right), as shown in the key above panel B. In addition to the categories corresponding to a domain of life, the “Tied” category indicates parameter combinations for which 2 or more domains of life had an identical percentage of organisms with 1 or more LCD, whereas the “Zero” category indicates parameter combinations for which none of the sampled organisms in all 4 domains of life contain an LCD in the indicated LCD class. LCD content increases for most LCD classes when progressing from Viruses➔Archaea➔Bacteria➔Eukaryota. For both figure panels, grid sections on the diagonal represent the primary LCD classes, whereas all other grid sections represent secondary LCD classes. (TIF) [file pcbi.1011372.s002.tif]

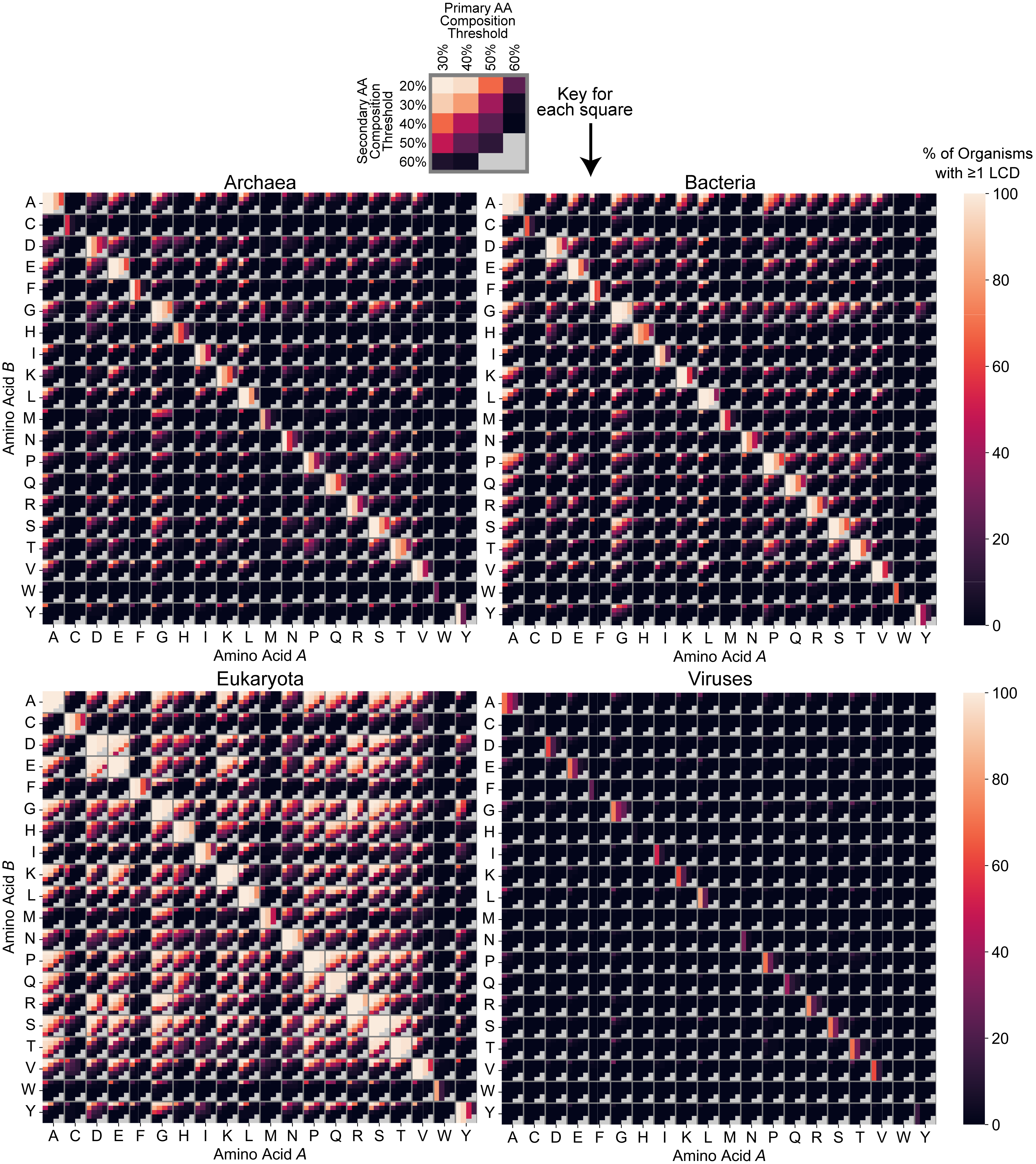

Supplement: S3 Fig — For each of the 50 randomly selected proteomes from each domain of life (those evaluated in S2 Fig), LCD-Composer searches were repeated with the primary composition threshold ranging from 30 to 60 (in steps of 10) and the secondary composition threshold ranging from 20 to 60 (in steps of 10). In these searches, window size was held constant at 20 amino acids. For each domain of life, the heatmap depicts the percentage of organisms containing at least 1 LCD for each combination of primary composition threshold, secondary composition threshold, and LCD class. Within each grid section representing a single LCD class, the y-axis represents the secondary composition threshold in the order 20➔30➔40➔50➔60 (from top to bottom), while the x-axis represents the primary composition threshold in the order 30➔40➔50➔60 (from left to right), as shown in the key above the figure. Grey squares in the lower-right corner of each grid square represent invalid composition thresholds where the sum of the primary and secondary thresholds exceeds 100%. As with varying window size, LCD content increases for most LCD classes when progressing from Viruses➔Archaea➔Bacteria➔Eukaryota regardless of the primary and secondary composition thresholds. For each heatmap, grid sections on the diagonal represent the primary LCD classes, whereas all other grid sections represent secondary LCD classes. For primary LCD classes, the composition threshold on the y-axis is ignored since only one composition threshold is relevant. Note that, for any given grid section, the composition thresholds (rather than the axis labels) determine which amino acid is considered the “primary” amino acid (larger composition threshold) and which is considered the “secondary” amino acid (smaller composition threshold) comprising the LCD class. (TIF) [file pcbi.1011372.s003.tif]

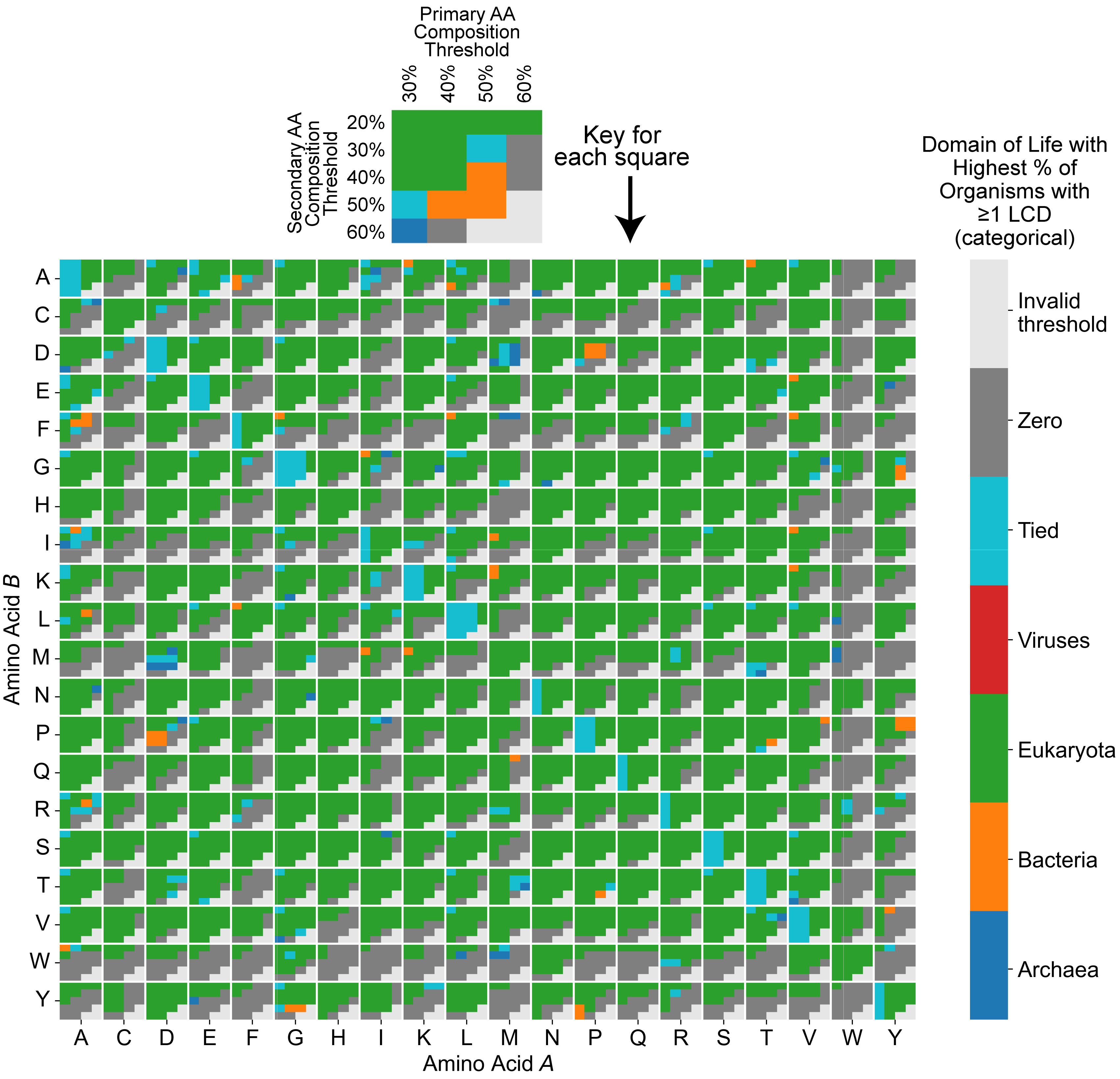

Supplement: S4 Fig — The heatmap is arranged identically to those in S3 Fig but depicts the domain of life with the highest percentage of organisms containing ≥1 LCD for each combination of primary composition threshold, secondary composition threshold, and LCD class. Grid sections on the diagonal represent the primary LCD classes, whereas all other grid sections represent secondary LCD classes. For primary LCD classes, the composition threshold on the y-axis is ignored since only one composition threshold is relevant. Note that, for any given grid section, the composition thresholds (rather than the axis labels) determine which amino acid is considered the “primary” amino acid (larger composition threshold) and which is considered the “secondary” amino acid (smaller composition threshold) comprising the LCD class. In addition to the categories corresponding to a domain of life, the “Tied” category indicates parameter combinations for which 2 or more domains of life had an identical percentage of organisms with 1 or more LCD, the “Zero” category indicates parameter combinations for which none of the sampled organisms in all 4 domains of life contain an LCD in the indicated LCD class, and the “Invalid threshold” category indicates composition threshold combinations for which the sum of the primary and secondary thresholds would exceed 100%. In the majority of “Tied” cases, the percentage of organisms containing an LCD was 100% for 2 or more organisms. For nearly all LCD classes, eukaryotes exhibit the highest percentage of organisms with ≥1 LCD regardless of the primary and secondary composition thresholds. (TIF) [file pcbi.1011372.s004.tif]

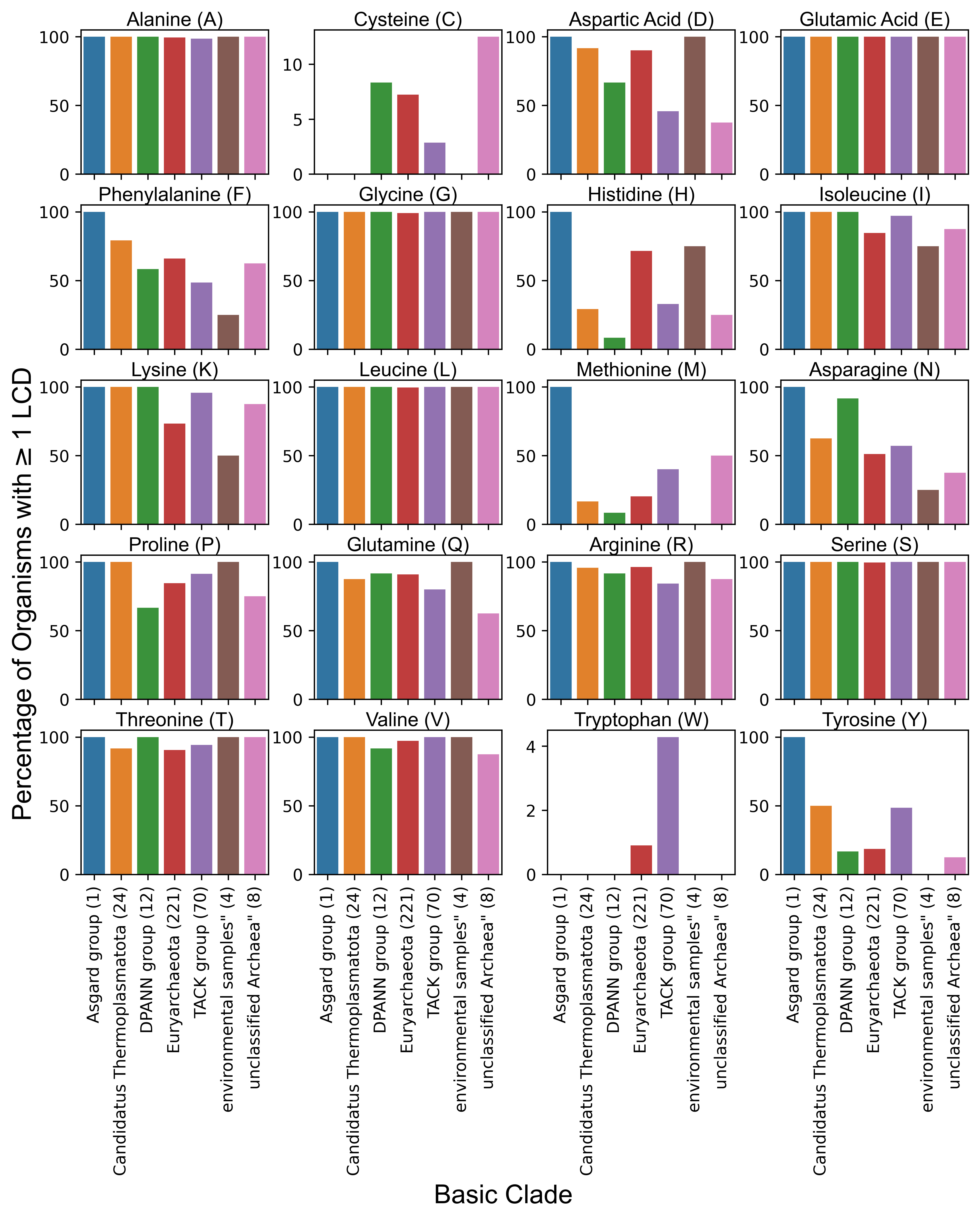

Supplement: S5 Fig — For each primary LCD class, organism-level LCD frequencies were evaluated separately for each basic archaeal clade. Clades were derived from taxonomic lineages and were the first term following the domain of life. Each bar color corresponds to only one clade (specified on the x-axis) throughout all subplots. Numbers in parentheses indicate the number of organisms evaluated for the corresponding clade. (TIF) [file pcbi.1011372.s005.tif]

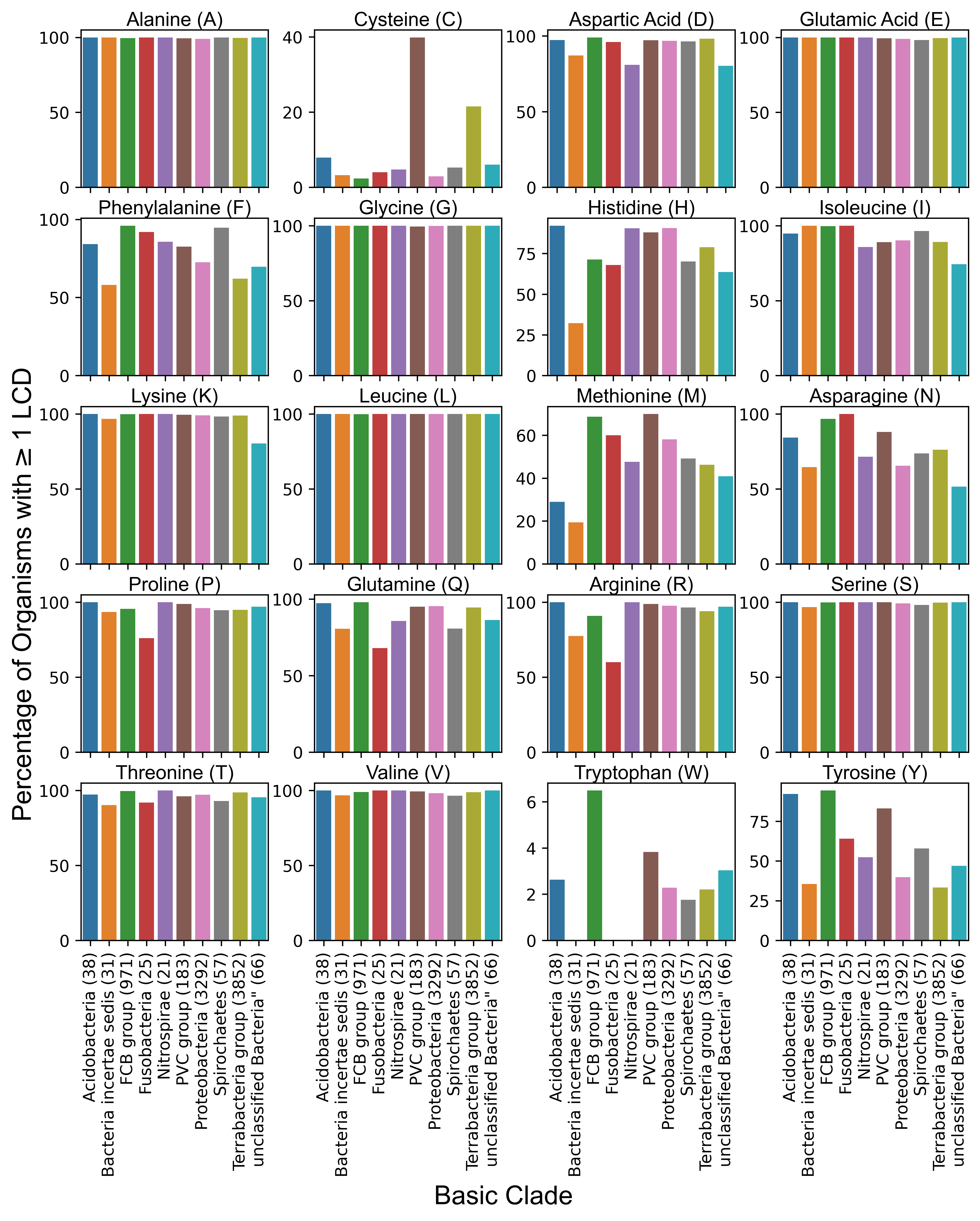

Supplement: S6 Fig — For each primary LCD class, organism-level LCD frequencies were evaluated separately for each basic bacterial clade. Figure details are as described for S5 Fig, except that only the top 10 bacterial clades (with respect to the number of organisms evaluated) are included for simplicity. (TIF) [file pcbi.1011372.s006.tif]

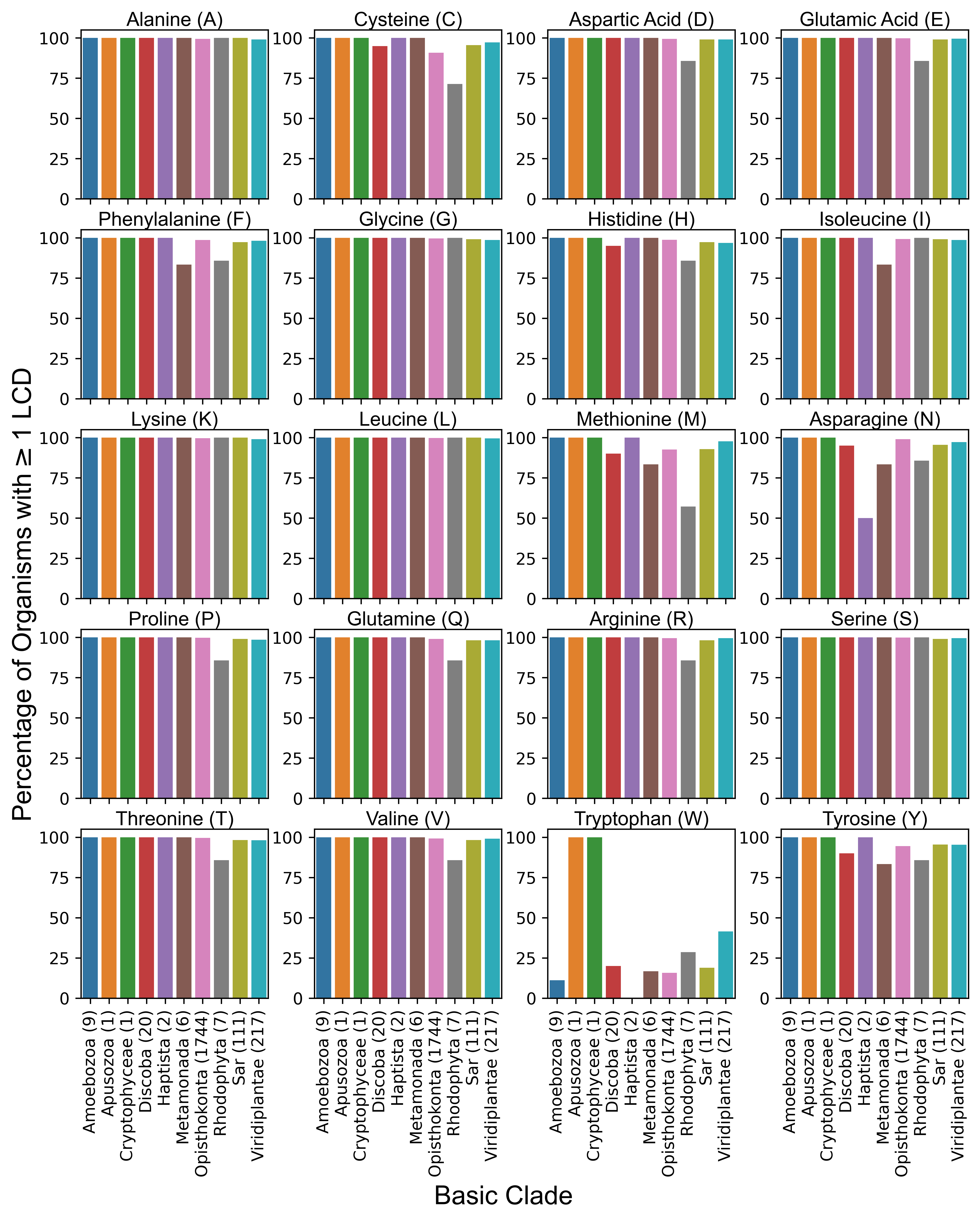

Supplement: S7 Fig — For each primary LCD class, organism-level LCD frequencies were evaluated separately for each basic eukaryotic clade. Figure details are as described for S5 Fig. (TIF) [file pcbi.1011372.s007.tif]

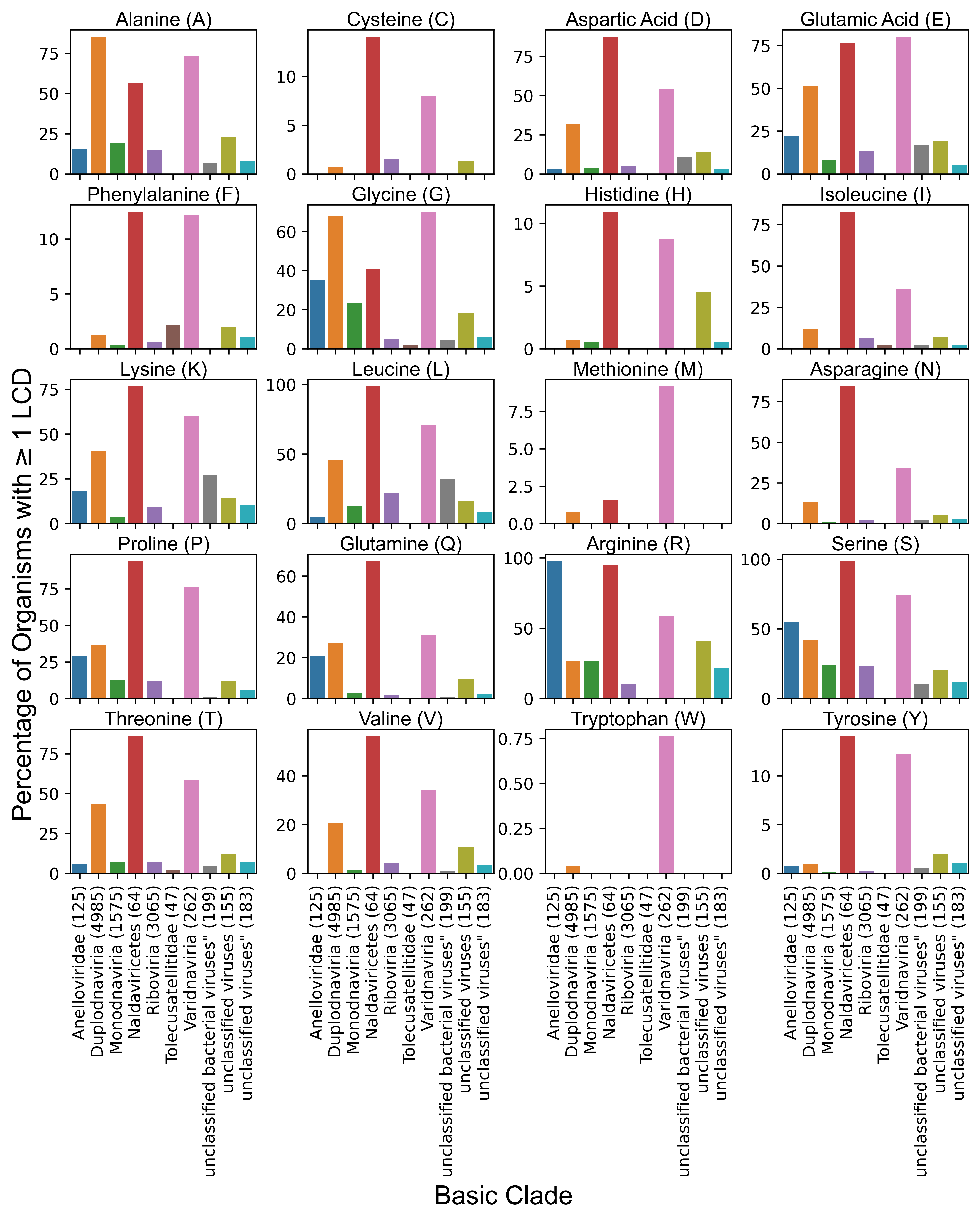

Supplement: S8 Fig — For each primary LCD class, organism-level LCD frequencies were evaluated separately for each basic viral clade. Figure details are as described for S5 Fig, except that only the top 10 viral clades (with respect to the number of organisms evaluated) are included for simplicity. (TIF) [file pcbi.1011372.s008.tif]

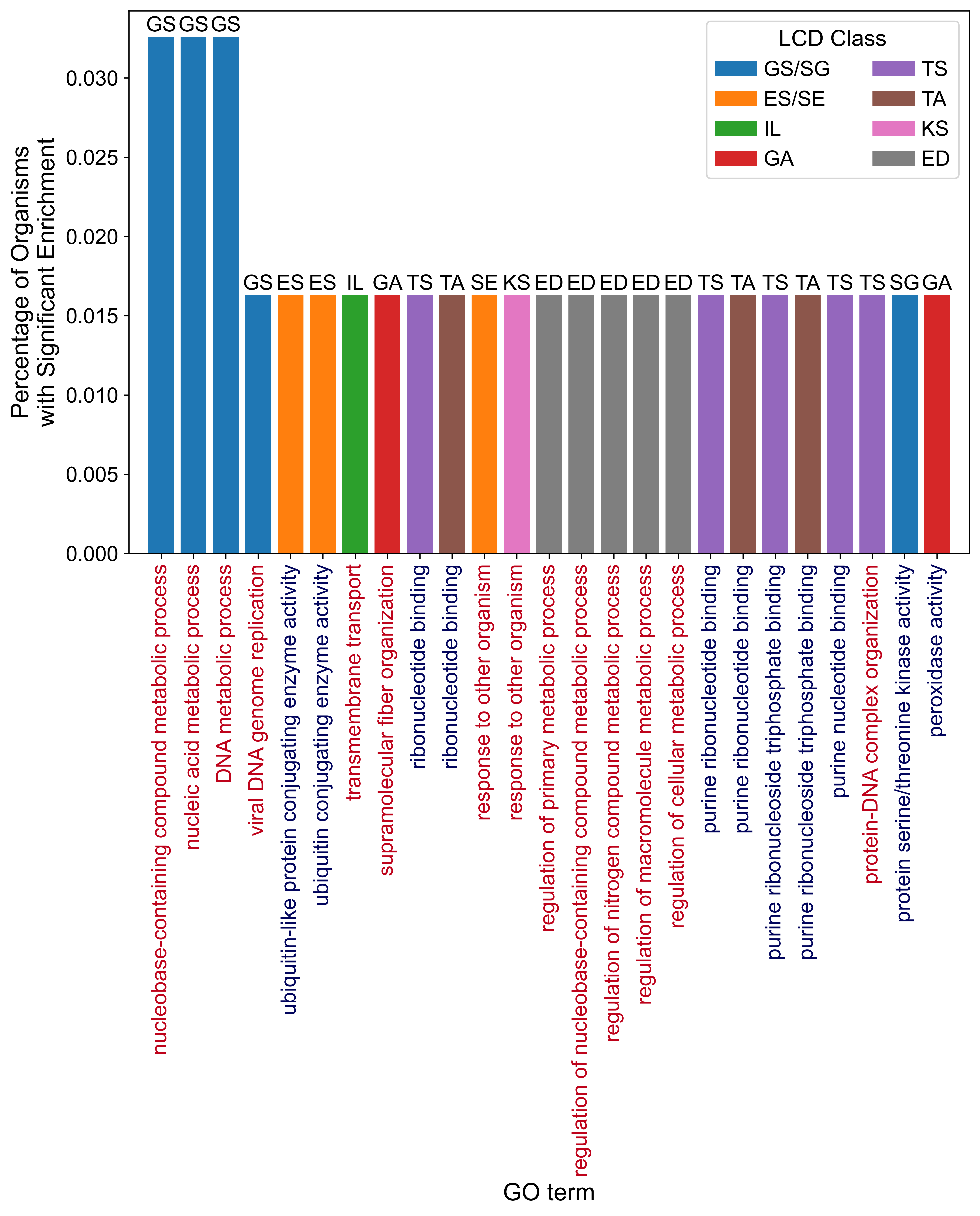

Supplement: S9 Fig — Top 25 GO term and LCD class pairs with respect to the percentage of viruses sharing significant enrichment for that pair. Only GO terms that were significantly enriched (Šidák-corrected p < 0.05) and had a minimum depth of 4 in the gene ontology are shown. Bar coloring and text coloring conventions are as described in the Fig 2 legend. (TIF) [file pcbi.1011372.s009.tif]

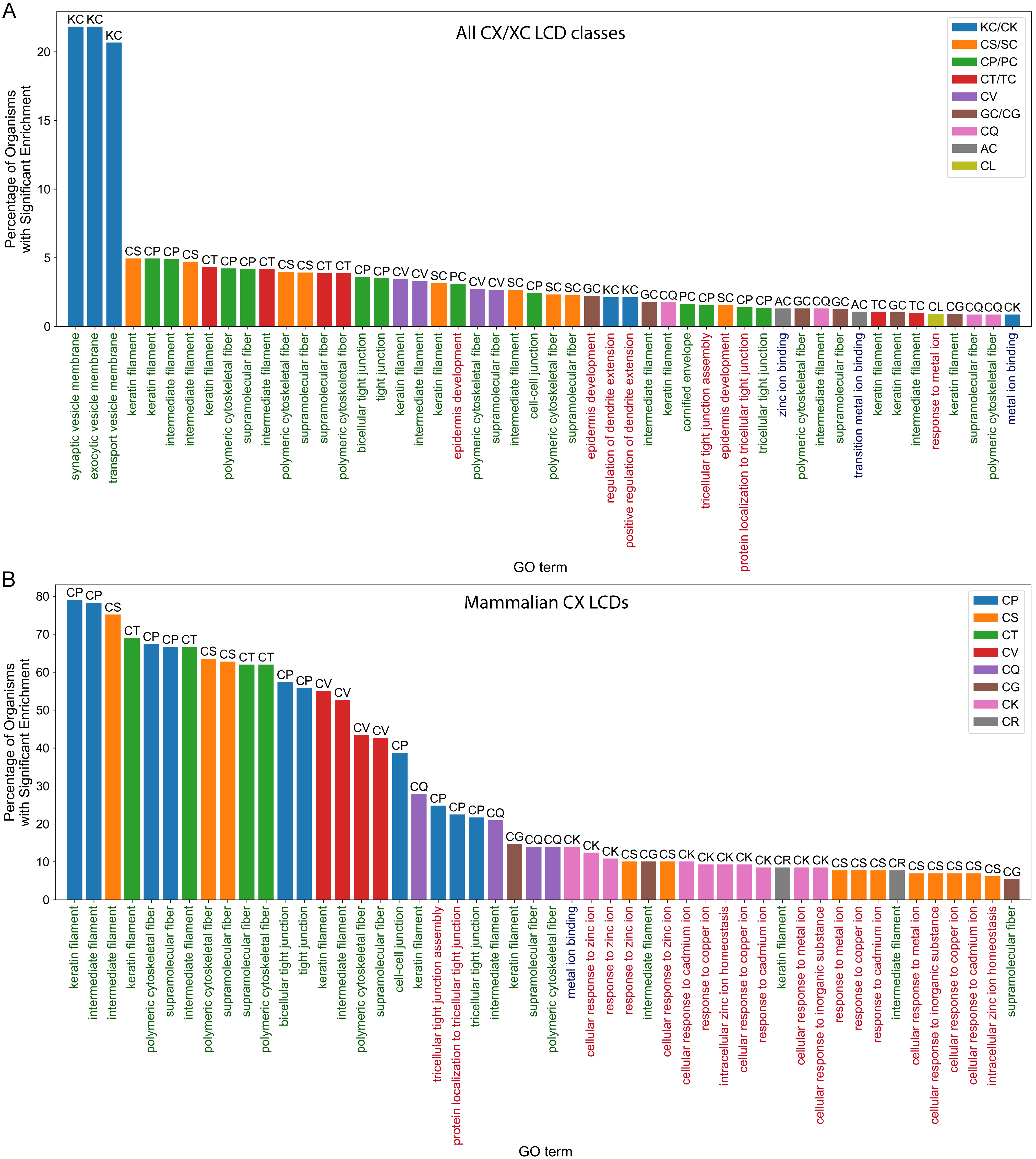

Supplement: S10 Fig — (A) Top-ranking functions associated with C-rich LCDs as indicated in Fig 3A but with inclusion of XC LCD classes. GO terms are sorted by the percentage of eukaryotic organisms with significant enrichment of the LCD class/GO term pair and are limited to the top 50 GO terms. (B) Top-ranking functions associated with CX LCDs in mammals only, limited to the top 50 GO terms. In both panels, bar color corresponds to LCD class, with reciprocal classes (e.g., CK and KC LCDs) assigned the same color for simplicity. GO terms on the x-axis are colored according to the GO-term category with Biological Process (BP) in red, Cellular Component (CC) in green, and Molecular Function (MF) in blue. Only GO terms that were significantly enriched (Šidák-corrected p < 0.05) and had a minimum depth of 4 in the gene ontology are shown. (TIF) [file pcbi.1011372.s010.tif]

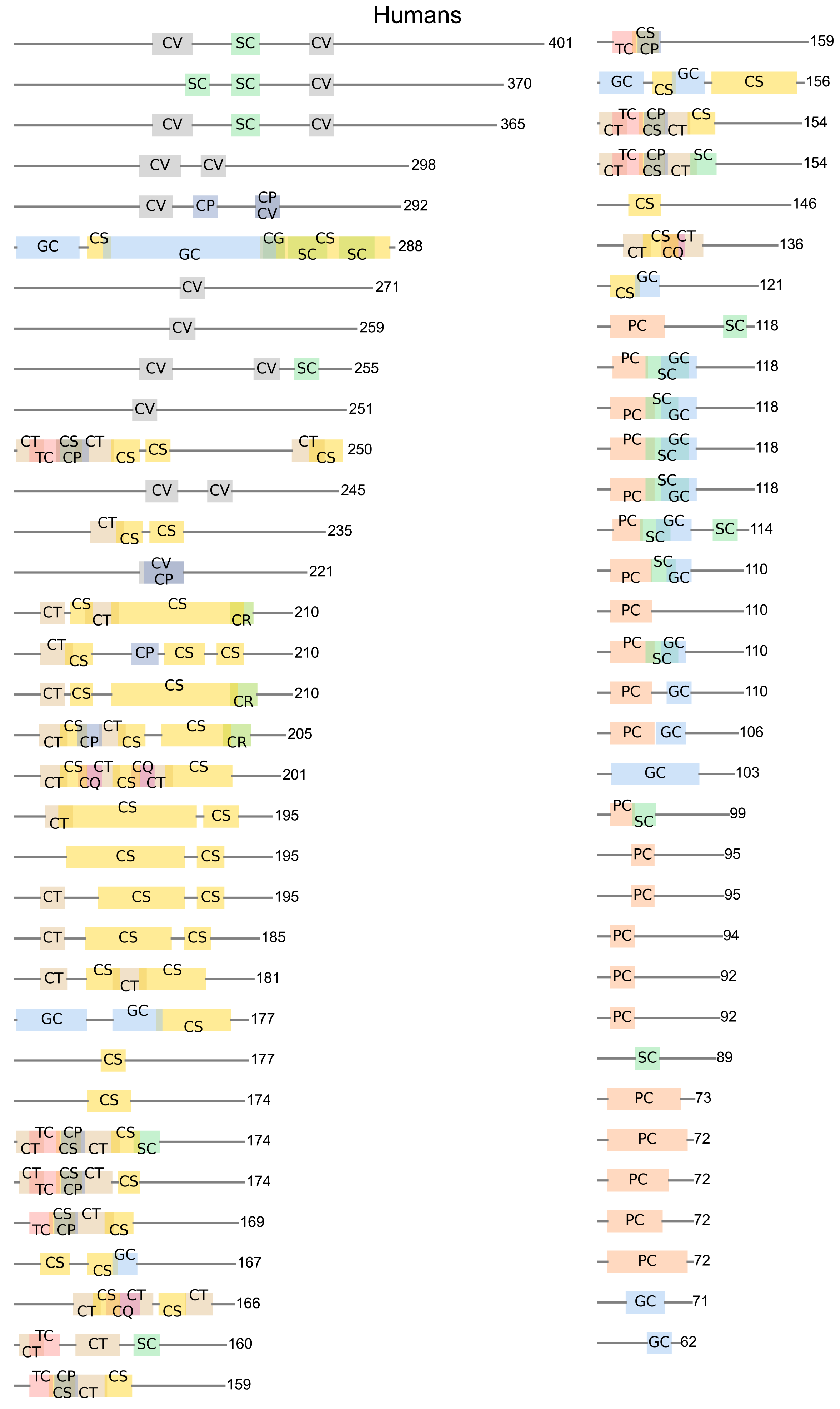

Supplement: S11 Fig — Schematic depicting LCD types and their locations within human keratin/keratin-associated proteins. LCD types are indicated by their two-letter abbreviations, and labels are staggered in cases where the LCDs overlap. (TIF) [file pcbi.1011372.s011.tif]

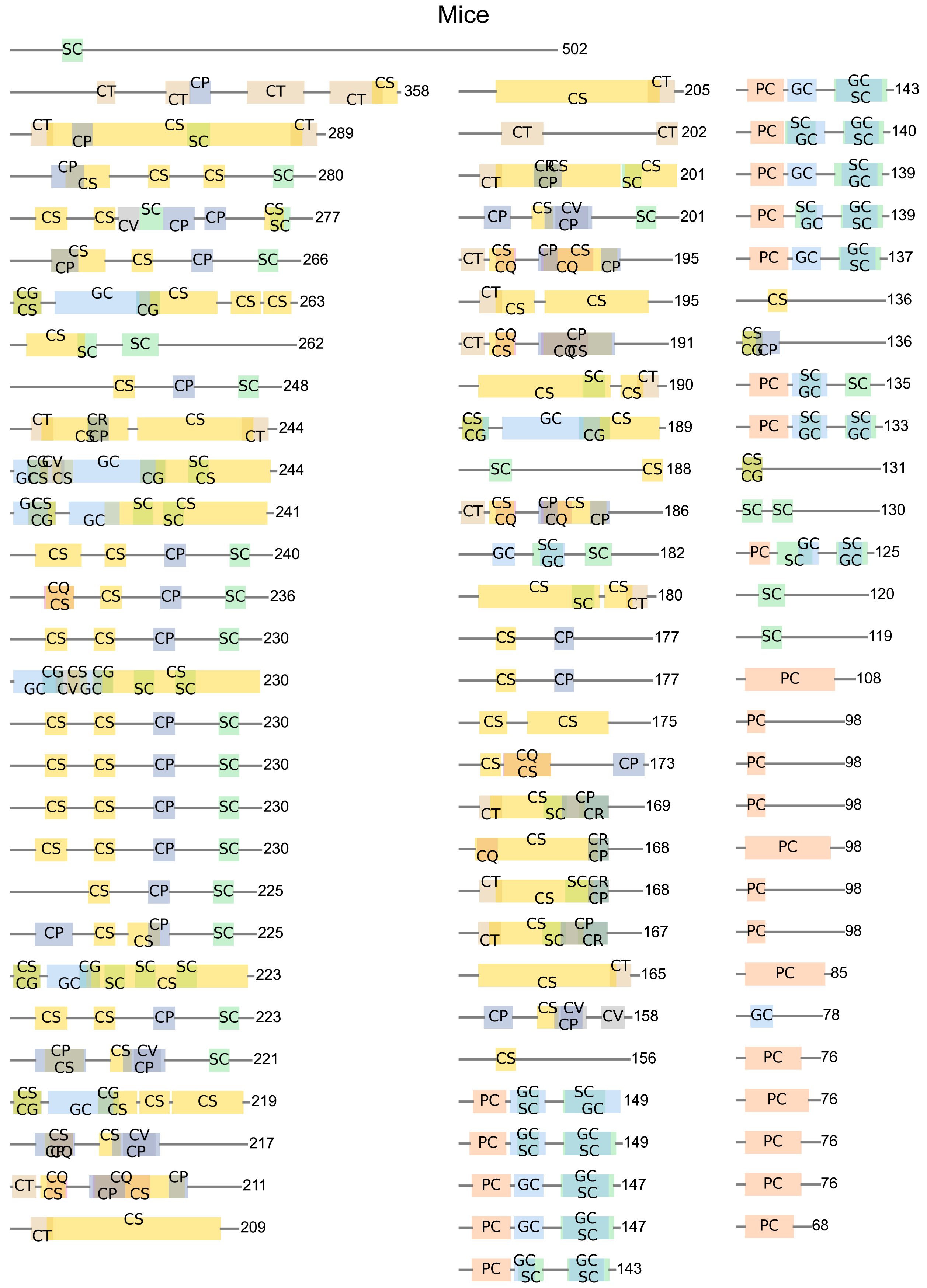

Supplement: S12 Fig — Same as S11 Fig, but for mouse keratin/keratin-associated proteins. (TIF) [file pcbi.1011372.s012.tif]

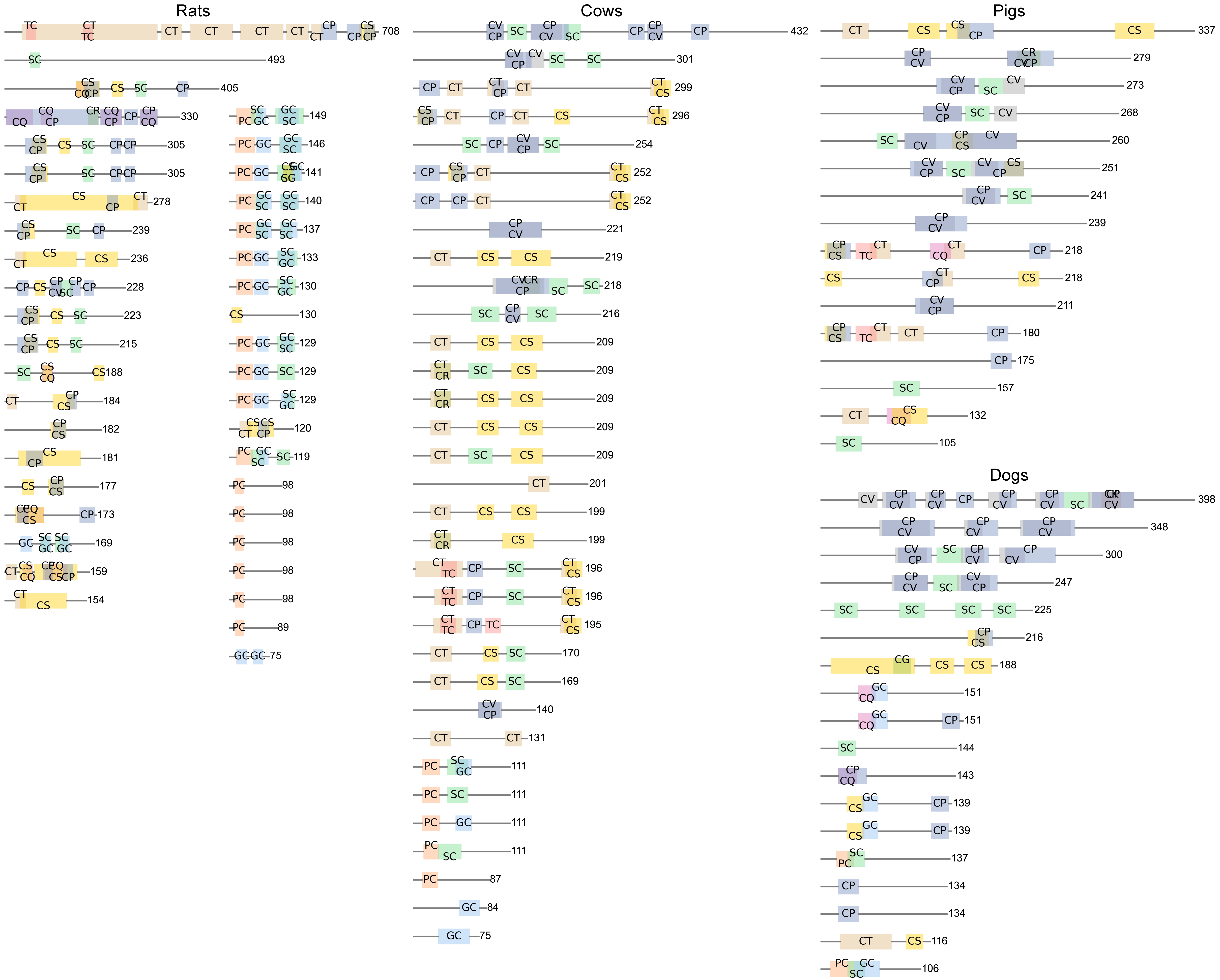

Supplement: S13 Fig — Same as S11 Fig, but for rat, cow, pig, and dog keratin/keratin-associated proteins. (TIF) [file pcbi.1011372.s013.tif]

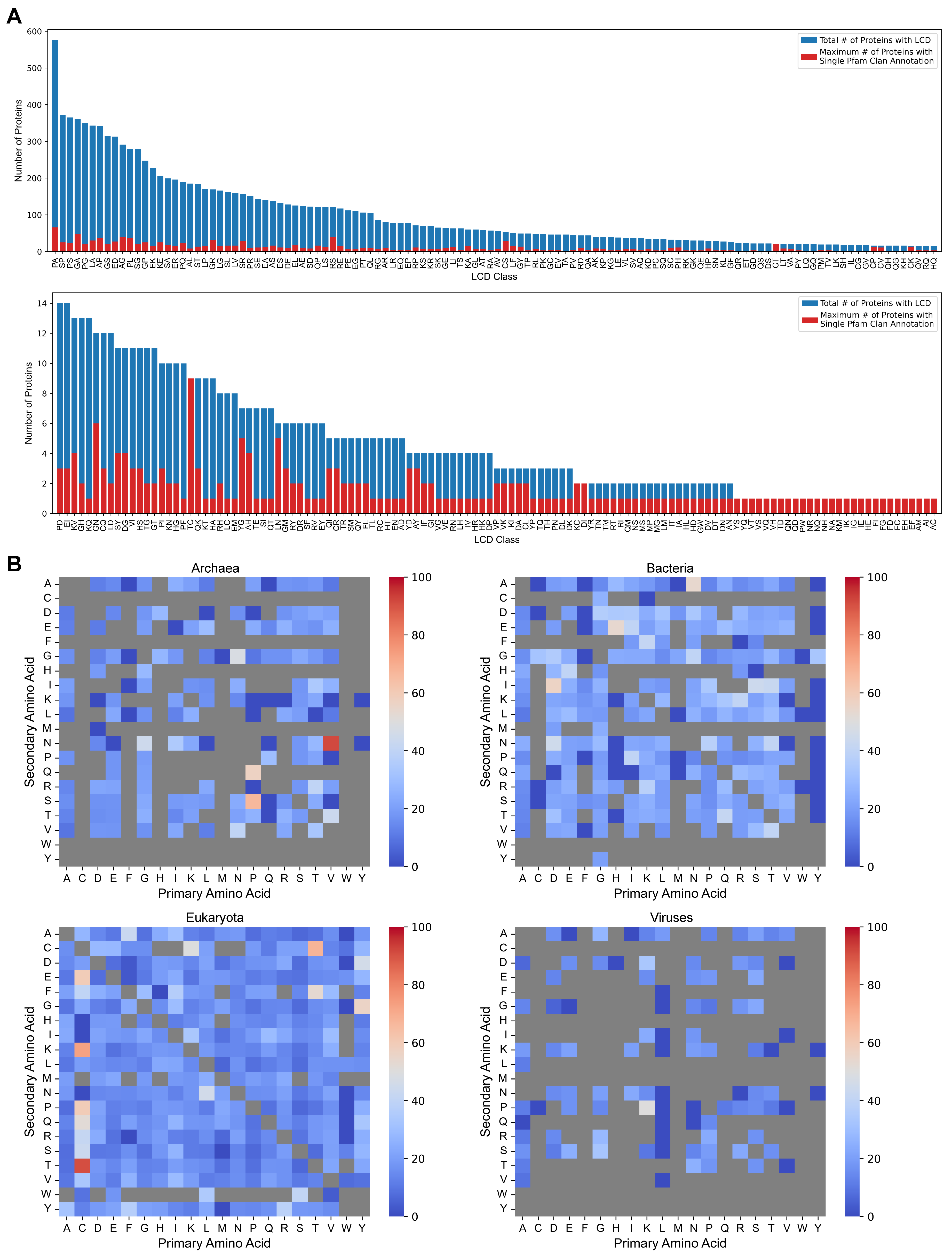

Supplement: S14 Fig — (A) Total number of proteins (blue) and the number of proteins associated with a single Pfam clan (red) were calculated for all LCD classes in the human proteome. (B) The average of maximum percentages of proteins associated with each LCD class was calculated across organisms within each domain. A minimum of 5 proteins were required to be included in the calculation to mitigate skewing of percentages by small sample sizes. Grey boxes indicate primary LCD classes and LCD classes for which none of the organisms had ≥5 associated proteins. (TIF) [file pcbi.1011372.s014.tif]

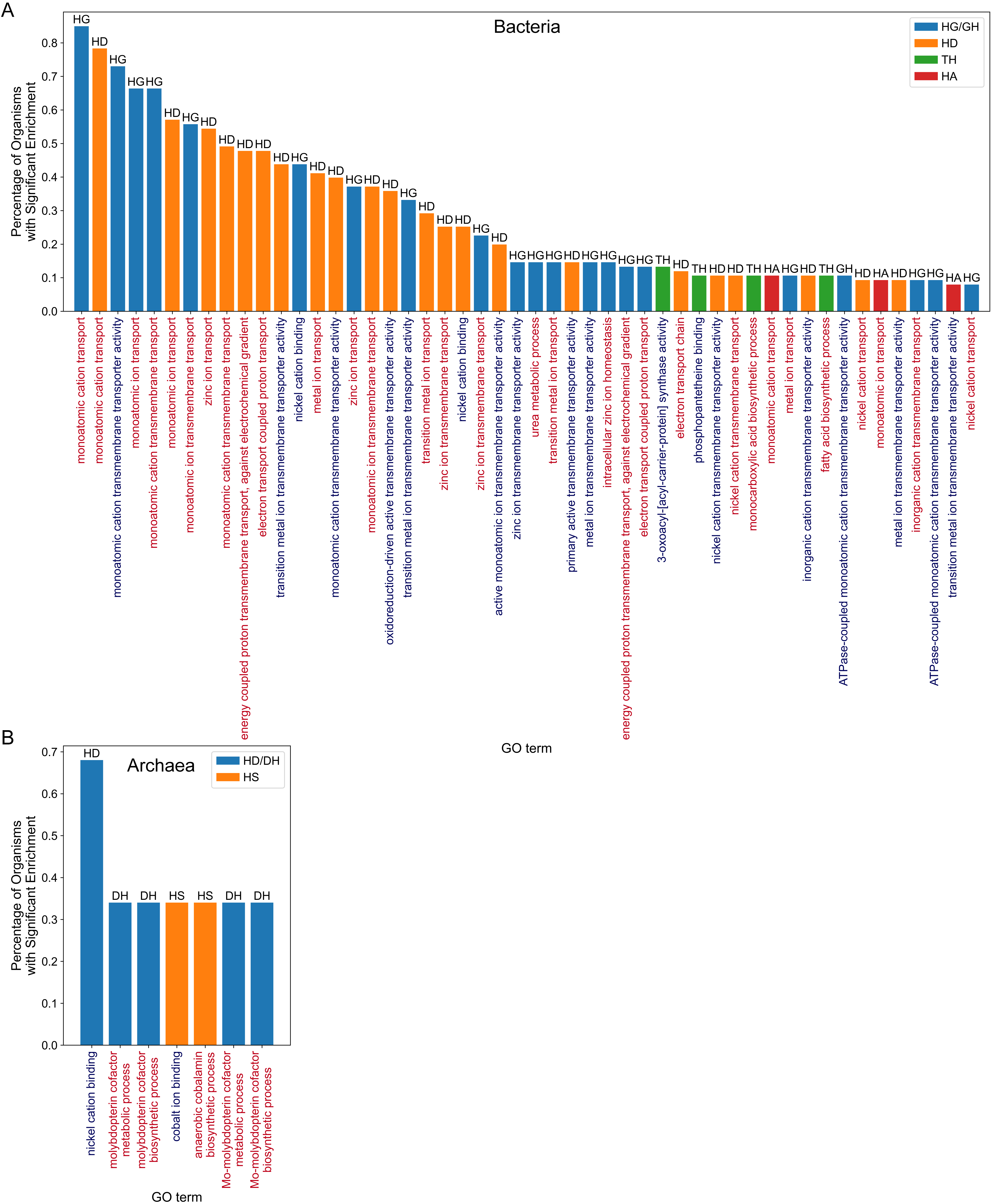

Supplement: S15 Fig — (A) GO terms associated with H-rich LCDs (including HX and XH LCD classes) in bacteria, sorted by the percentage of bacterial organisms with enrichment of each LCD class/GO term pair and limited to the top 50 pairs. (B) GO terms associated with H-rich LCDs in archaea. All LCD class/GO term pairs with significant enrichment in at least one organism are displayed. In both panels, bar color corresponds to LCD class, with reciprocal classes (e.g., HG and GH LCDs) assigned the same color for simplicity. GO terms on the x-axis are colored according to the GO-term category with Biological Process (BP) in red, Cellular Component (CC) in green, and Molecular Function (MF) in blue. Only GO terms that were significantly enriched (Šidák-corrected p < 0.05) and had a minimum depth of 4 in the gene ontology are shown. (TIF) [file pcbi.1011372.s015.tif]

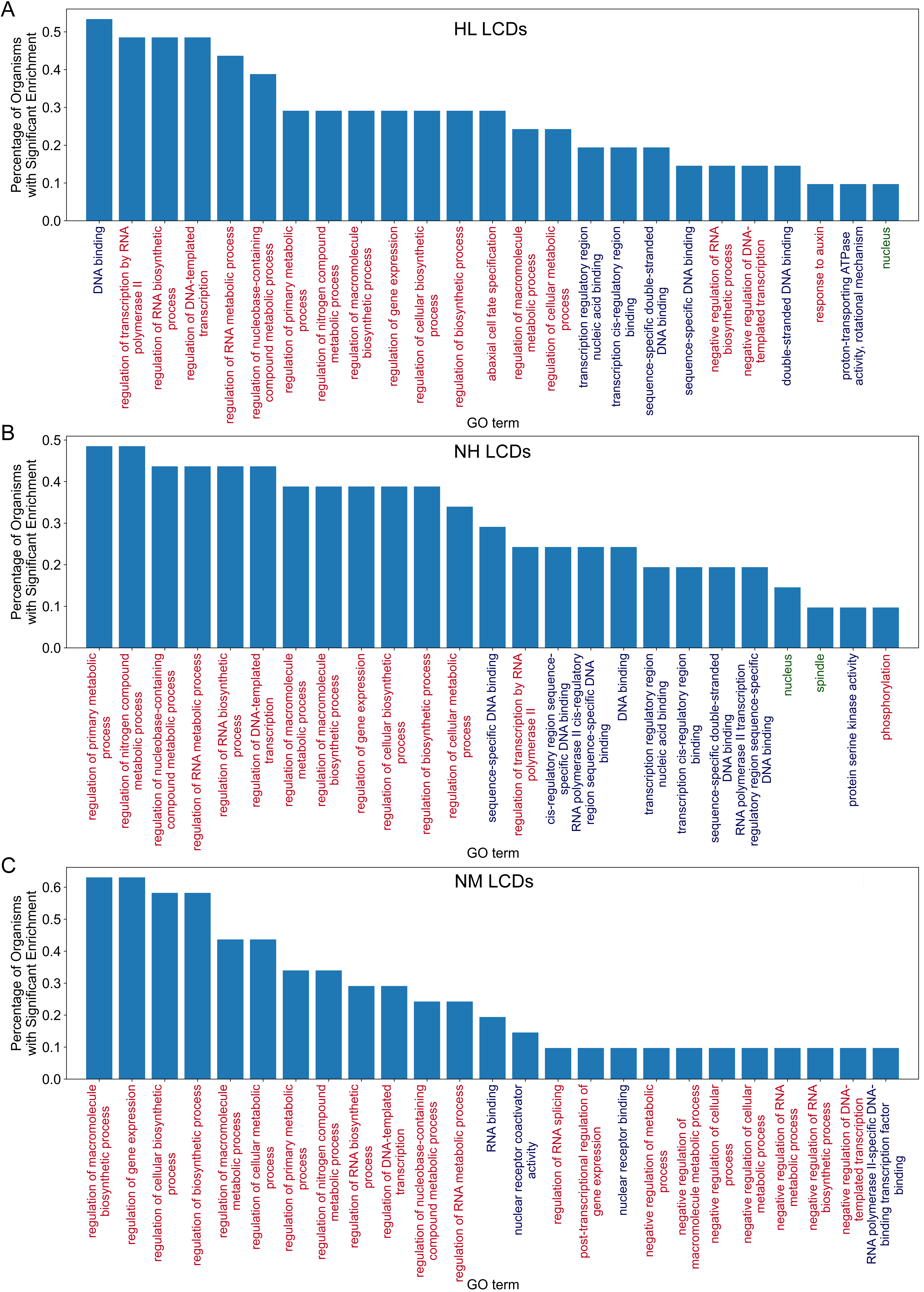

Supplement: S16 Fig — Bar plots indicate the percentage of eukaryotic organisms exhibiting significant enrichment for each LCD class/GO term pair for HL LCDs (A), NH LCDs (B), and NM LCDs (C). LCD class/GO term pairs are sorted by the percentage of eukaryotic organisms sharing significant enrichment, limited to the top 25 LCD class/GO term pairs. In all panels, GO terms on the x-axis are colored according to the GO-term category with Biological Process (BP) in red, Cellular Component (CC) in green, and Molecular Function (MF) in blue. Only GO terms that were significantly enriched (Šidák-corrected p < 0.05) and had a minimum depth of 4 in the gene ontology are shown. (TIF) [file pcbi.1011372.s016.tif]

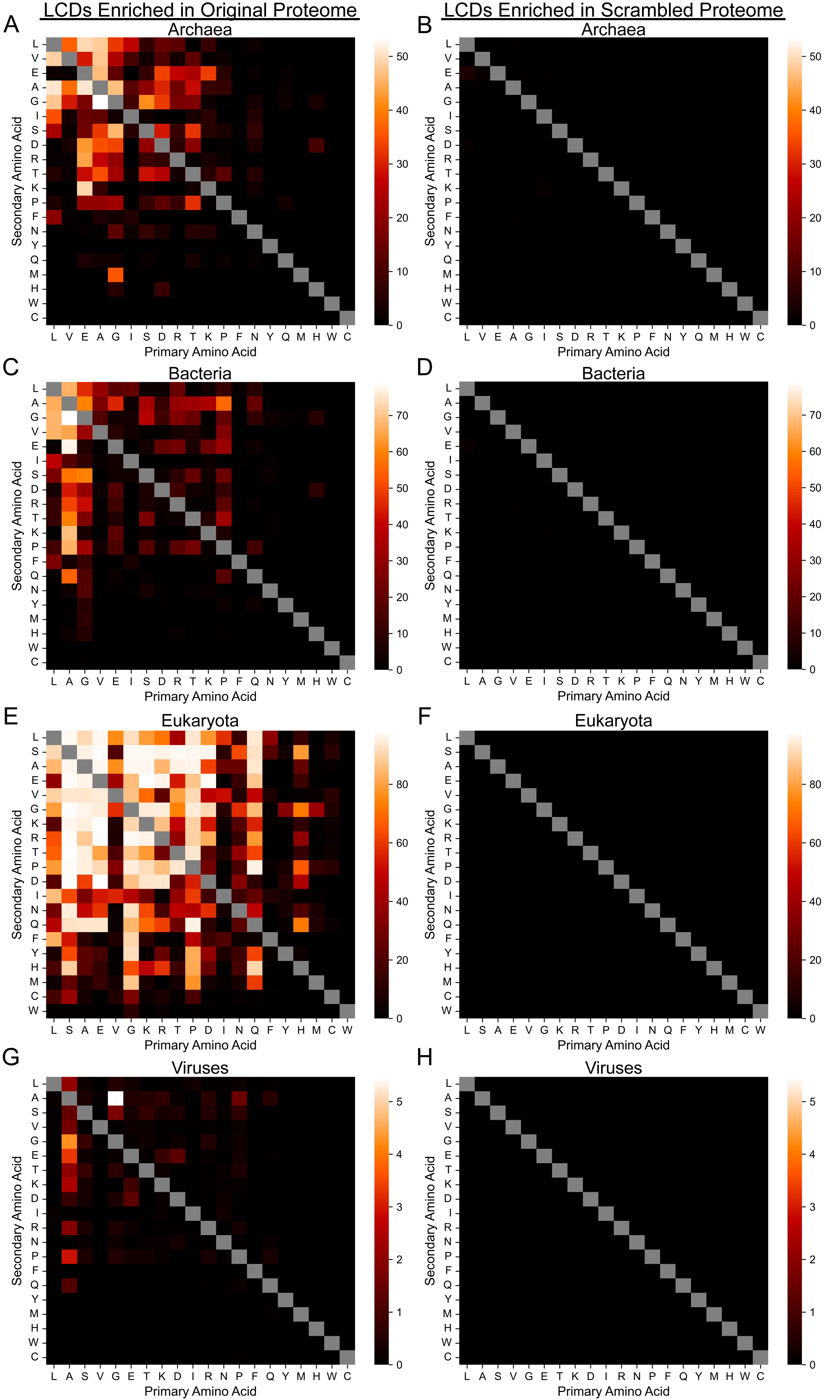

Supplement: S17 Fig — The percentage of organisms with enrichment (positive lnORs) or depletion (negative lnORs) was calculated as described for Fig 6 but without imposing a statistical significance threshold. The panels in the left column (panels A, C, E, and G) indicate the percentage of organisms with LCD enrichment in the original proteome for each LCD class. The panels in the right column (panels B, D, F, and H) indicate the percentage of organisms with LCD depletion in the original proteome for each LCD class. For each pair of heatmaps corresponding to a single domain of life, the heatmap scales are identical to facilitate direct comparison. (TIF) [file pcbi.1011372.s017.tif]

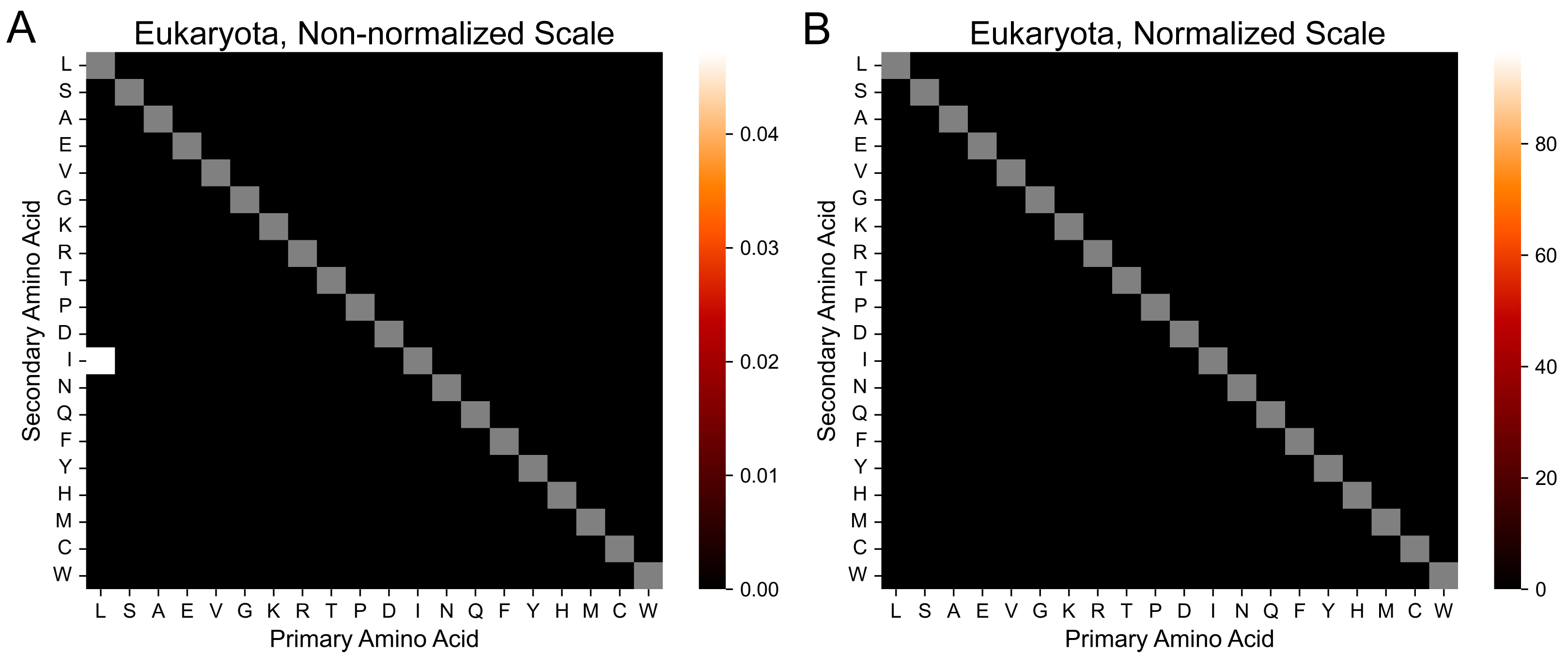

Supplement: S18 Fig — The percentage of organisms with statistically significant depletion (negative lnORs) was calculated as described for Fig 6. Heatmaps depict percentages for eukaryotes only, as all off-diagonal values for archaea, bacteria, and viruses were exactly 0: however, all data underlying these heatmaps, as well as data corresponding to the other domains of life, can be found in the supplementary data available at [33]. Panels in the left column depict heatmaps with scales set by the minimum and maximum values within each heatmap. Panels in the right column depict heatmaps with scales identical to those in Fig 6 to facilitate direct comparison. (TIF) [file pcbi.1011372.s018.tif]

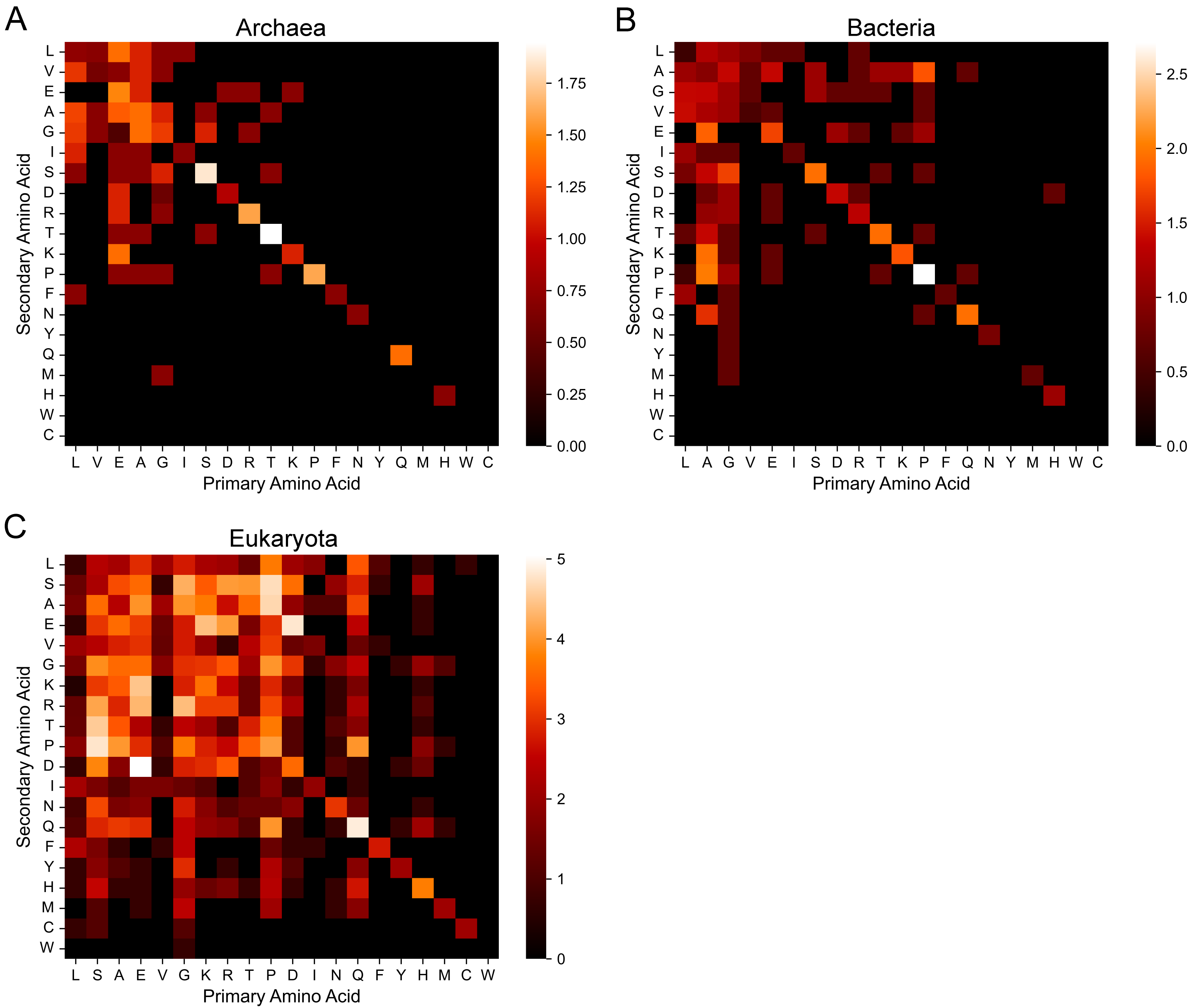

Supplement: S19 Fig — For each organism, the natural logarithm of the odds ratio (lnOR) for each LCD class was used to quantify the degree of LCD enrichment or depletion in the original proteome relative to a scrambled version of that proteome. Heatmaps depict the median lnOR for each LCD class among archaea (A), bacteria (B), and eukaryotes (C). LCD enrichment data are not shown for viruses since all median lnORs are exactly 0: however, all data underlying these heatmaps, as well as data corresponding to viruses, can be found in the supplementary data available at [33]. No negative median lnOR values were observed for any of the domains of life. The diagonals indicate the median lnOR for each primary LCD class. For LCD classes in which the number of LCDs in either the original or scrambled proteomes were 0, a value of 1 was added to all cells in the contingency table to calculate a biased lnOR (see Methods). (TIF) [file pcbi.1011372.s019.tif]

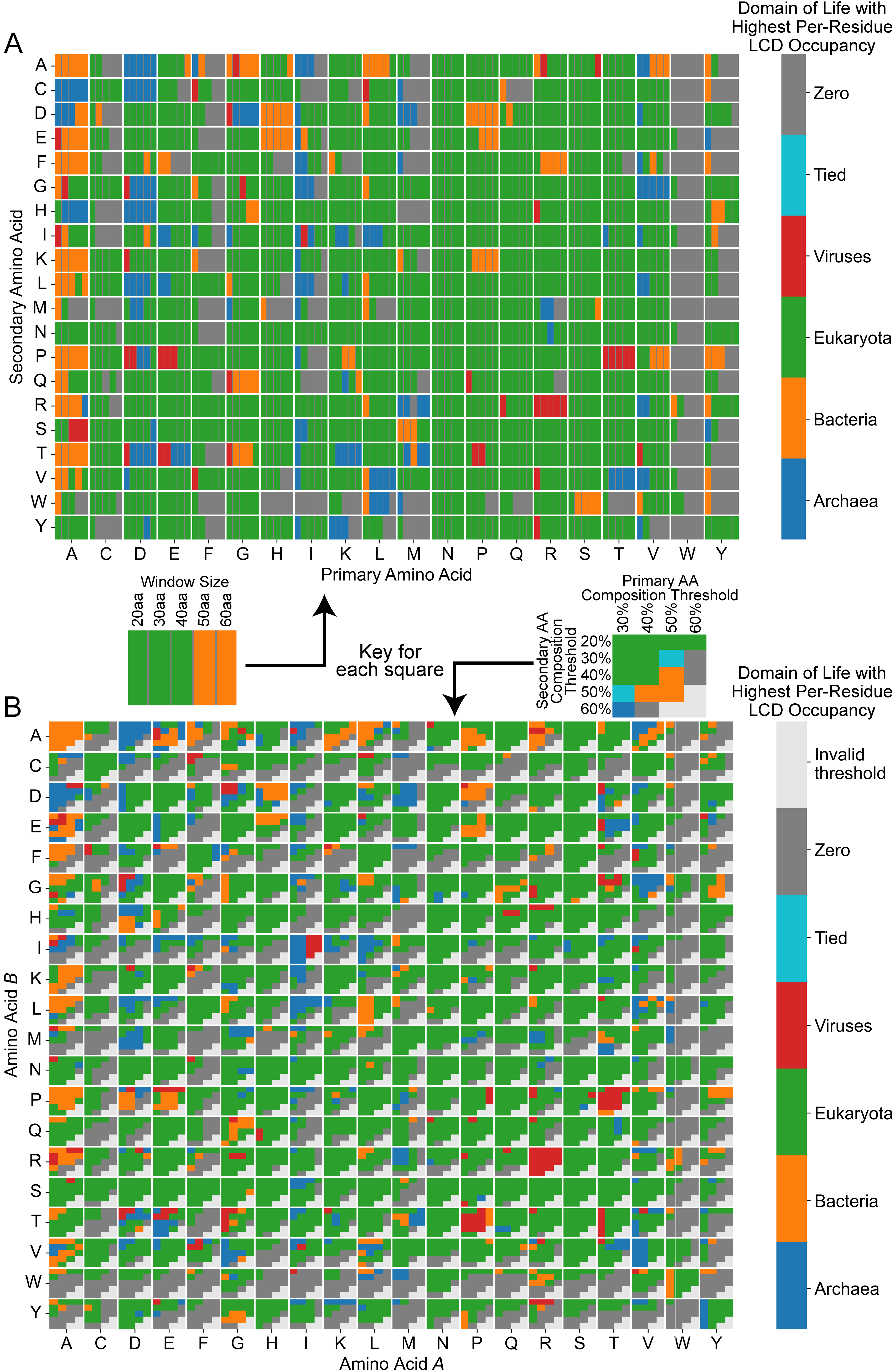

Supplement: S20 Fig — (A) Domain of life with the highest per-residue LCD occupancy for each LCD class while varying the window size parameter during the LCD searches. (B) Domain of life with the highest per-residue LCD occupancy for each LCD class while varying the composition threshold parameters during the LCD searches. In both panels, per-residue LCD occupancies were calculated from the same randomly selected subset of organisms evaluated for S2–S4 Figs. Figure layouts, formatting, and color categories are as described for S2 and S4 Figs, respectively. (TIF) [file pcbi.1011372.s020.tif]

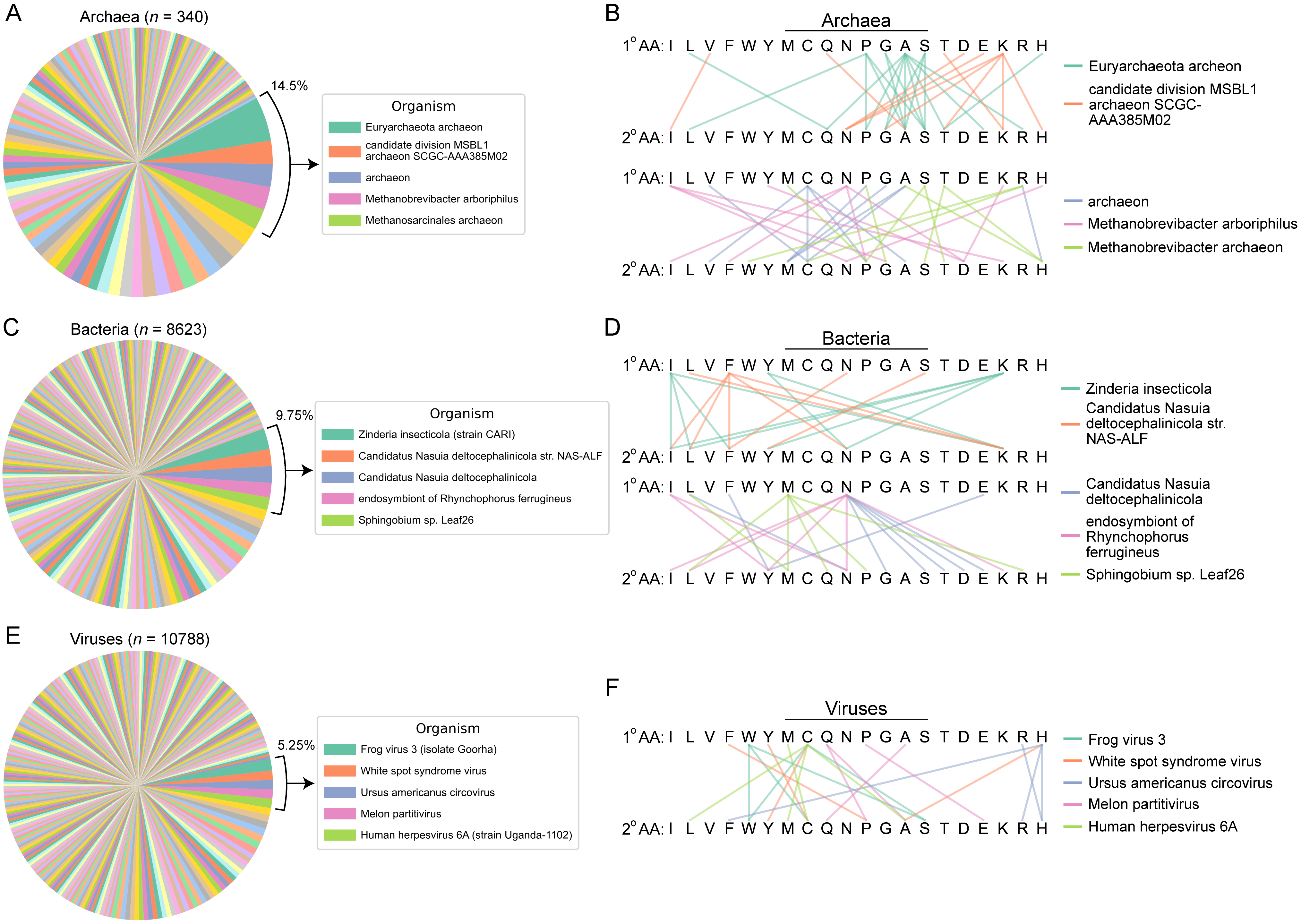

Supplement: S21 Fig — (A) Pie chart indicating the assignment of LCD classes (400 total) to the archaeal organism achieving the highest per-residue LCD occupancy. Each wedge represents a single organism associated with the overall highest per-residue occupancy observed among archaea. Wedge size indicates the number of LCD classes for which the single organism corresponding to that wedge achieved the highest per-residue occupancy. (B) Linkage maps indicating the types of LCD classes for which the organism contributed the maximum per-residue occupancy value for archaea. The first row of amino acids in each linkage map indicates the primary amino acid in the LCD class, and lines connected to the second row of amino acids indicate the secondary amino acid in the LCD class. Lines connecting identical amino acids indicate that the organism contributed the maximum per-residue occupancy value for the primary LCD class as a whole (e.g., the W-rich primary LCD class). LCD classes without connecting lines are those for which the organism did not contribute the maximum per-residue occupancy value. Identical analyses were performed for bacteria (C,D) and viruses (E,F). For all pie charts, the top five organisms are indicated in the legend. Out of necessity, the color palette was repeated in each pie chart, though each color cycle represents a different set of organisms. (TIF) [file pcbi.1011372.s021.tif]

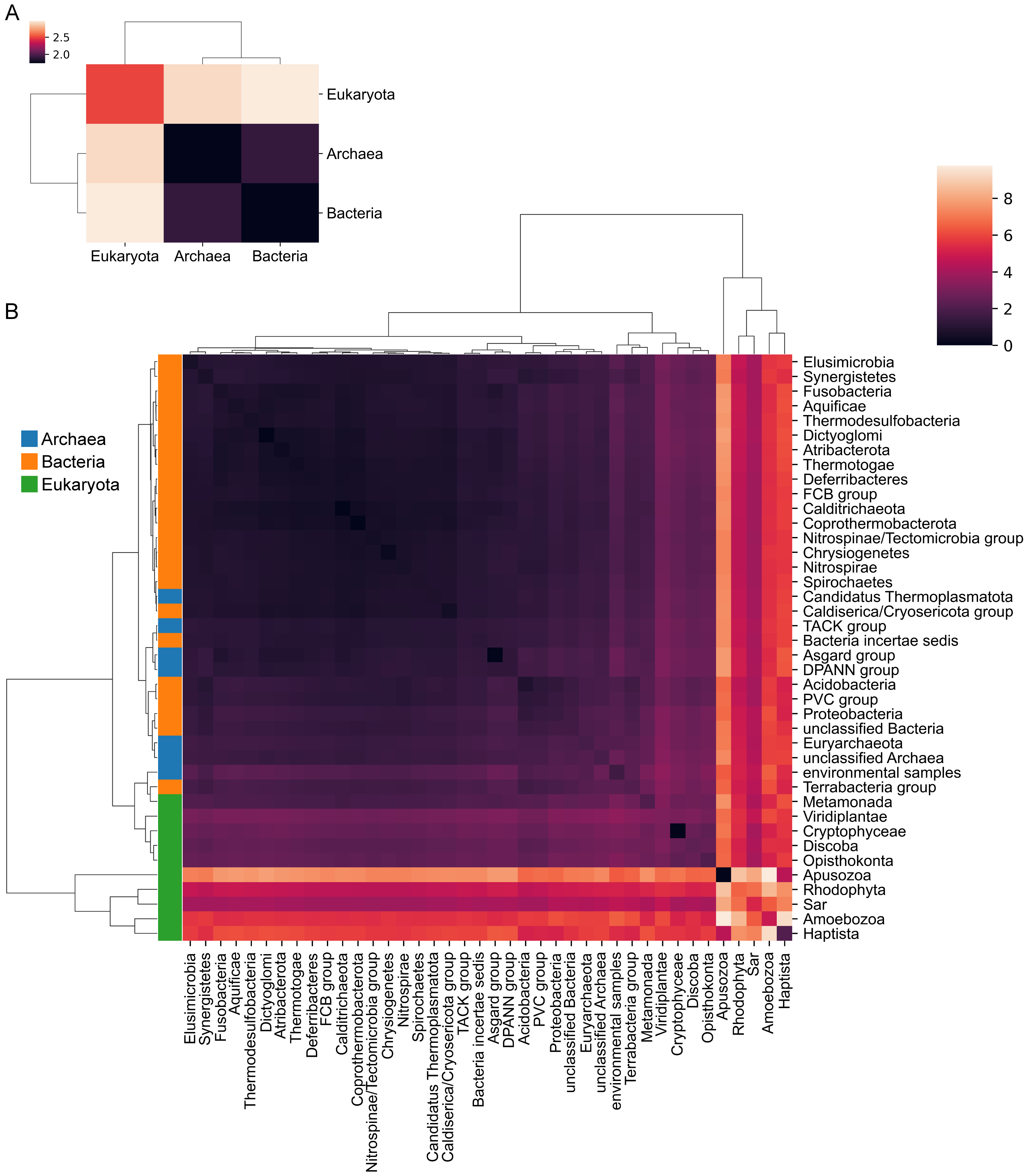

Supplement: S22 Fig — Per-residue occupancy values for all 380 secondary classes were calculated from each proteome and compared in a pairwise fashion by calculating the Manhattan distance between per-residue occupancy arrays for all possible pairs of organisms, excluding self-comparisons which evaluate to 0. (A) Heatmap indicating the average pairwise Manhattan distance within and between domains of life. (B) Heatmap indicating the average pairwise Manhattan distance within and between basic clades (i.e., the first term following the domain of life in the taxonomic lineage). Colors to the left of the heatmap indicate the domain of life corresponding to each clade on the y-axis. In both panels, clustering was calculated using complete linkage and Manhattan distance. Viruses were excluded from comparisons due to their exceptionally small proteomes, leading both to sparse arrays (the complete absence of LCDs for most classes) and volatile individual values (existing LCDs occupying relatively large percentages of their small proteomes). Note that the dendrogram does not represent evolutionary relationship per se: it simply depicts the degree of clustering based on the distances between whole-proteome LCD content. (TIF) [file pcbi.1011372.s022.tif]
